# The Global Burden of Migraine: A 30-Year Trend Review and Future Projections by Age, Sex, Country, and Region

**DOI:** 10.1007/s40122-024-00690-7

**Published:** 2024-12-11

**Authors:** Lingkang Dong, Wenqi Dong, Yuchen Jin, Yumeng Jiang, Zhuangzhuang Li, Dongzhen Yu

**Affiliations:** 1 Department of Otolaryngology Head and Neck Surgery, Shanghai Sixth People’s Hospital Affiliated to Shanghai Jiao Tong University School of Medicine, Shanghai, China; 2 Department of Otolaryngology, Sun Yat-Sen Memorial Hospital, Sun Yat-Sen University, Guangzhou, China

**Keywords:** Migraine, DALYs, GBD 2021, Global trends, Predictive analysis

## Abstract

**Introduction:**

Migraine is a prevalent neurological disorder causing significant disability worldwide. Despite extensive research on specific populations, comprehensive analyses of global trends are remains limited.

**Methods:**

We extracted incidence, prevalence, and disability-adjusted life years (DALYs) data for migraine from the Global Burden of Disease 2021 database. Trends were analyzed across regions, age groups, sexes, and sociodemographic index (SDI) using estimated annual percentage changes (EAPC). Predictive models (ARIMA) were used to forecast trends to 2050.

**Results:**

From 1990 to 2021, the global burden of migraine significantly increased: prevalence increased by 58.15%, from 732.56 million to 1.16 billion cases, and incidence increased by 42.06%. The DALYs also increased by 58.27%. There were differences between the sexes: female individuals had higher absolute rates of migraine incidence and prevalence, but male individuals exhibited a four- to five-fold more rapid increase than female individuals in these parameters. Adolescents (< 20 years old) have the fastest growth in prevalence and DALYs. Regionally, high SDI regions having the highest age-standardized rate (ASR) and low SDI regions having the lowest ASR in DALYs. East Asia and Latin America exhibited the most significant increases in migraine burden, whereas Southeast Asia exhibited the most pronounced decrease. Predictive analysis suggests prevalence will continue to rise until 2050, particularly among male individuals and adolescents.

**Conclusions:**

The global burden of migraine has significantly escalated from 1990 to 2021, with female individuals bearing a greater burden but male individuals showing a faster growth rate. Adolescents also face a rapidly rising prevalence. Disparities across SDI regions, countries, age groups, and sexes emphasize the need for targeted public health strategies. Focused interventions are required to mitigate the growing impact of migraines on global health, particularly among male individuals and adolescents.

**Supplementary Information:**

The online version contains supplementary material available at 10.1007/s40122-024-00690-7.

## Key Summary Points

**Table:** 

***Why carry out this study?***
Migraine affected over 1 billion people worldwide in 2021, making it one of the top causes of disability and imposing immense personal, economic, and social burdens
Current research mainly focuses on the female population, with few studies systematically analyzing trends across the entire population
This study aimed to explore long-term trends in migraine burden across gender, age, country, and regional groups to fully understand the changes and distribution of migraine
***What was learned from the study?***
Between 1990 and 2021, the burden of migraine rose significantly worldwide. Although female individuals experienced the highest burden, male individuals showed a faster rate of increase, with younger and working-age individuals most affected
Migraine prevalence and disability-adjusted life years (DALYs) are projected to continue rising through 2050, especially among male individuals and adolescents

## Introduction

Migraine is a common chronic neurological disorder affecting over one billion people worldwide, and generating substantial individual, economic, and socioeconomic burdens [1, 2]. Approximately 14% of the global population experience the condition every year, making migraine the second leading contributor to the global burden of neurological diseases, and it affects many young people [3–5].

Migraine predominantly affects female individuals, and most research focuses on female populations. The prevalence of migraine is significantly higher among female individuals, and they are more likely to report migraine-related disabilities [3, 6]. However, the burden of migraine in male individuals cannot be overlooked. Male individuals are less likely to report high-intensity pain or seek medical treatment, partly due to cultural perceptions of traditional sex/gender roles [7, 8]. Consequently, migraine in male individuals may be underdiagnosed [6, 9], and there have been few male-specific studies, which limits our understanding of the condition in male individuals [10]. These factors may adversely affect quality of life for male individuals and increase the risk of other health issues. For example, untreated migraine may lead to chronic conditions and comorbidities [11, 12]. Therefore, there is a need for more public health efforts to improve the accuracy of migraine diagnoses in male individuals, as well as to provide better interventions and policy support.

While previous studies have explored the burden of migraine in female individuals and specific age groups [13–15], few have systematically analyzed trends across all age groups and sexes within large populations. The most recent global analysis utilized data from 2019 but was limited to incidence, without comprehensive coverage of prevalence and disability-adjusted life years (DALYs) [16]. To address these gaps, we used the latest Global Burden of Disease (GBD) 2021 database to conduct a thorough assessment of migraine burden from 1990 to 2021 [17].

Our study provides a comprehensive analysis of migraine incidence, prevalence, and DALYs on a global, regional, and national scale. We used statistical approaches such as estimated annual percentage change (EAPC), frontier analysis, and predictive modeling to evaluate trends across 204 countries and 21 regions, considering variations by age, sex, and sociodemographic index (SDI). This research aims to provide critical insights into the evolving global burden of migraine and inform future public health policies, emphasizing targeted interventions for both sexes and across different demographic groups.

## Methods

### Data Source

The GBD 2021 results database, accessible through the GBD Collaborative Network website (https://www.healthdata.org/), provides a comprehensive assessment of the global incidence rate, prevalence rate, and DALYs of 371 diseases and injuries in 204 countries and regions from 1990 to 2021; it also integrates the latest epidemiological data with standardized methods. Detailed descriptions of the study design and methods are recorded in GBD publications [18–20]. We extracted data on migraine incidence, prevalence, and DALY rates. Then we calculated age-standardized rate (ASR) and EAPC. This study did not require ethics committee approval, it uses publicly available, de-identified, and aggregated data from the Global Burden of Disease (GBD) 2021 database, which poses no risk to individual privacy.

### Definitions

Migraine, a disabling primary headache disorder, is typically characterized by recurrent, moderate to severe unilateral pulsatile headaches [21]. According to the International Classification of Diseases, 9th and 10th versions (ICD-9 and ICD-10), it is classified under codes 346–346.93 and G43–G43.919 [21–25].

The sociodemographic index (SDI) is used to represent the socioconomic and developmental levels of countries and regions, ranging from 0 (lowest) to 1 (highest) levels of development [26]. For our analysis, countries were grouped into five SDI categories to assess variations in health outcomes, providing valuable insights for researchers and policymakers [19, 20].

### Predictive Analysis

To forecast future trends in migraine incidence, prevalence, and DALYs over the next 29 years, we used the autoregressive integrated moving average (ARIMA) model. ARIMA models provide a sophisticated method for capturing the dynamics of time series data while incorporating additional relevant variables [27, 28]. Data from 1990 to 2021 served as the training sample for fitting the ARIMA model, with the optimal model determined using the *auto.arima()* function based on the Akaike Information Criterion and Bayesian Information Criterion values.

### Statistical Analysis

We conducted a comprehensive analysis of global and regional trends in migraine incidence, prevalence, and DALYs from 1990 to 2021 to evaluate the overall burden of the disease. These metrics were expressed per 100,000 individuals in the population, with their 95% uncertainty interval (UI). Additionally, we calculated two key indicators from the GBD study: the ASR and the EAPC, as well as age-standardized incidence rate (ASIR), age-standardized prevalence rate (ASPR), and age-standardized DALY rate (ASDR). The ASR is used to adjust for variations in age structure, allowing for more accurate comparisons of disease prevalence or mortality across different regions or time periods [29]. The EAPC measures the average annual percentage change of an indicator over time, offering insights into the rate and direction of trends [13]. The EAPC was calculated using the following formulas:

$$ \ln \, \left( {{\text{ASR}}} \right) \, = \, \alpha \, + \, \beta X \, + \, \varepsilon , $$

$$ {\text{EAPC }} = \, 100 \, \times \, \left( {e^{\beta } {-} \, 1} \right), $$

where *X* represents the calendar year, *α* is the intercept, *β* denotes the annual change in ln (ASR), and *ε* is an error term. The 95% confidence intervals (CI) for EAPC were also calculated on the basis of this linear model [14, 30]. A negative EAPC value and an upper limit for the 95% CI that is below zero indicate a declining ASR trend, whereas a positive EAPC value and a lower limit for the 95% CI that is above zero indicate an increasing trend. If the 95% CI includes zero, this implies a stable ASR trend. Data cleaning, computations, and visualizations were performed using R software (ver. 4.2.3; R Development Core Team, Vienna, Austria), with visualizations created using the ggplot2 package, and final edits completed using Microsoft PowerPoint software (ver. 365; Microsoft Corporation, Redmond, WA, USA).

## Results

### Global Trends in Migraine Burden (1990–2021)

From 1990 to 2021, the global burden of migraine significantly increased (Supplementary Material Table S1, Fig. S1). The number of prevalence cases increased from 732.56 million in 1990 to 1.16 billion in 2021, representing an approximate increase of 58.15%. Concurrently, the ASPR increased from 14,027.65 to 14,246.55 per 100,000 population, with an EAPC of 0.06. During the same period, incidence rose from 63.50 million to 90.18 million cases, an increase of 42.06%. Moreover, the DALYs associated with migraine significantly increased from 27.41 million to 43.38 million, an increase of 58.27%. Additionally, the ASDR increased from 526.76 to 532.70 per 100,000 population, with an EAPC of 0.05.

### Differences by Sex

In 1990, the incidence of migraine in female individuals was approximately 39.50 million, with an ASIR of 1,435.6 per 100,000. By 2021, this figure had increased to 55.43 million cases with an ASIR of 1,438.9 per 100,000 and an EAPC of 0.03. For male individuals, the incidence of migraine in 1990 was 23.99 million, with an ASIR of 846.91 per 100,000. By 2021, this had increased to 34.75 million cases with an ASIR of 876.55 per 100,000 and an EAPC of 0.13, which was fourfold greater than in female individuals (Supplementary Material Table S1).

**Fig. 1:**
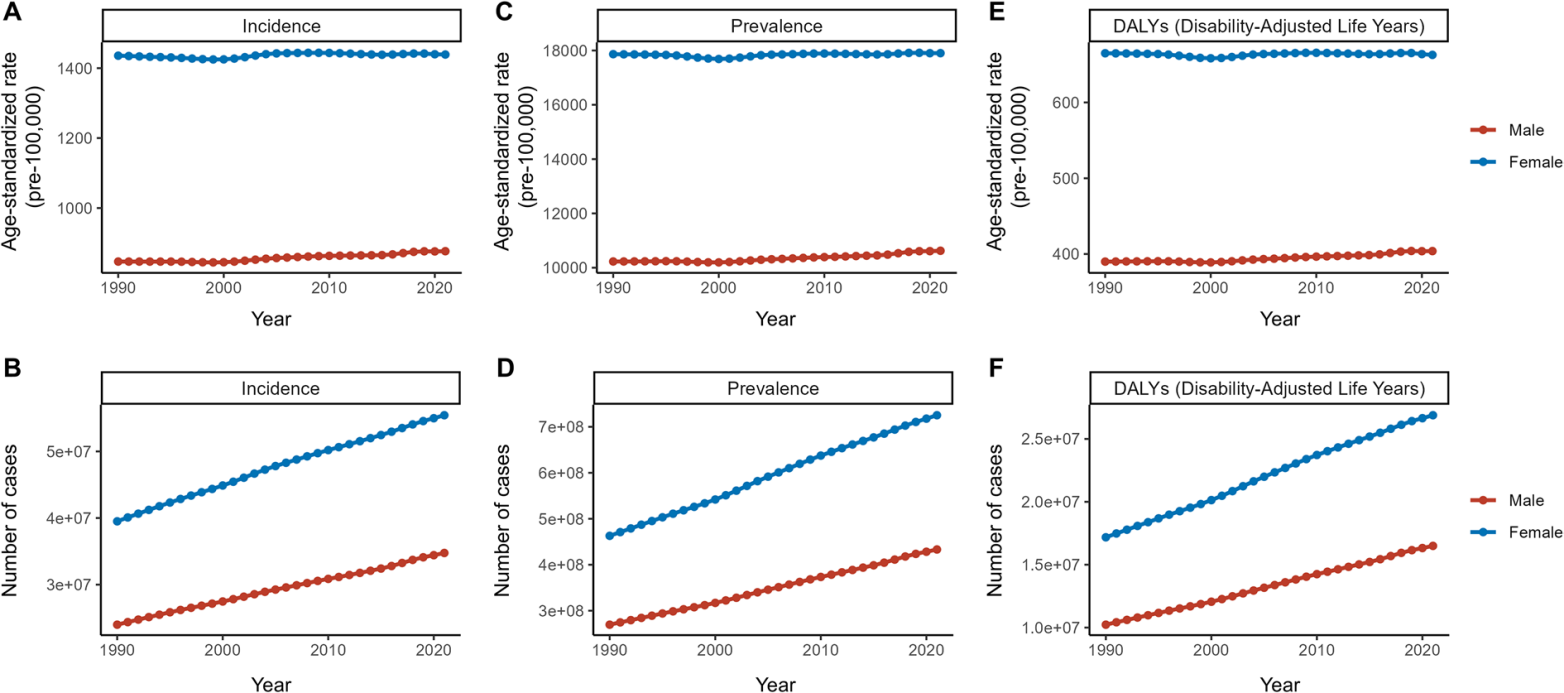
Trends in migraine burden by sex from 1990 to 2021. ASIR (**A**) and incidence cases (**B**), ASPR (**C**) and prevalence cases (**D**), and ASDR (**E**) and DALYs cases (**F**) for migraine between the sexes from 2019 to 2021. *ASDR* age-standardized DALY rate, *ASIR* age-standardized incidence rate, *ASPR* age-standardized prevalence rate, *DALYs* disability-adjusted life years

In 1990, the prevalence of migraine in female individuals was 462.90 million, with an ASPR of 17,864.62 per 100,000. By 2021, this had increased to 725.24 million, with an ASPR of 17,902.6 per 100,000 and an EAPC of 0.02. In 1990, the prevalence of migraine in male individuals was 269.67 million, with an ASPR of 10,229.25 per 100,000. By 2021, this had increased to 433.19 million, with an ASPR of 10,624.2 per 100,000 and an EAPC of 0.13. These data demonstrate that although female individuals have exhibited higher absolute rates of migraine incidence and prevalence in the past, the rate of increase in these parameters have been more rapid in male individuals (Supplementary Material Table S1, Fig. 1, Fig. S2).

### Age-Specific Trends

**Fig. 2:**
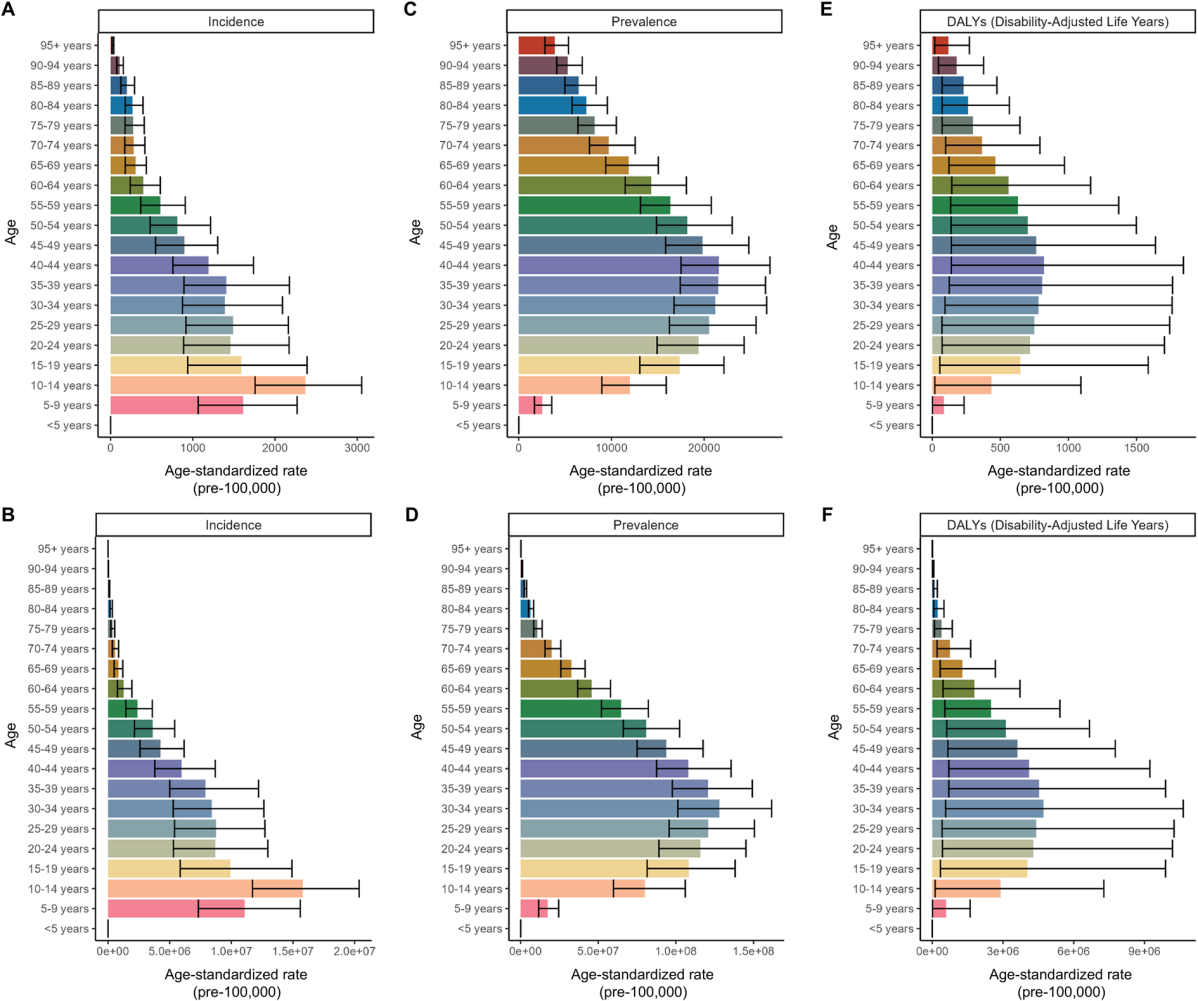
Cross-section  (2021) of incidence rate, prevalence rate, and DALY rate for migraine across age groups. ASIR (**A**) and incidence cases (**B**), ASPR (**C**) and prevalence cases (**D**), and ASDR (**E**) and DALYs cases (**F**) for migraine. *ASDR* age-standardized DALY rate, *ASPR* age-standardized prevalence rate, *DALYs* disability-adjusted life years

For both male and female individuals, the ASIR for migraine peaked at 10–14 years old, whereas the ASPR and ASDR increased from the 10–14 years age group to the 40–44 years age group (Supplementary Material Table S2). The ASIR for the 10–14 years age group was 2368.93; the ASIR for the 40–44 years age group was 21,579.93, and the ASDR was 820.79. Prevalence and DALY cases were highest in the 30–34 years age group because this had the greatest population, with 128.09 million and 4.71 million, respectively (Fig. 2).

**Fig. 3:**
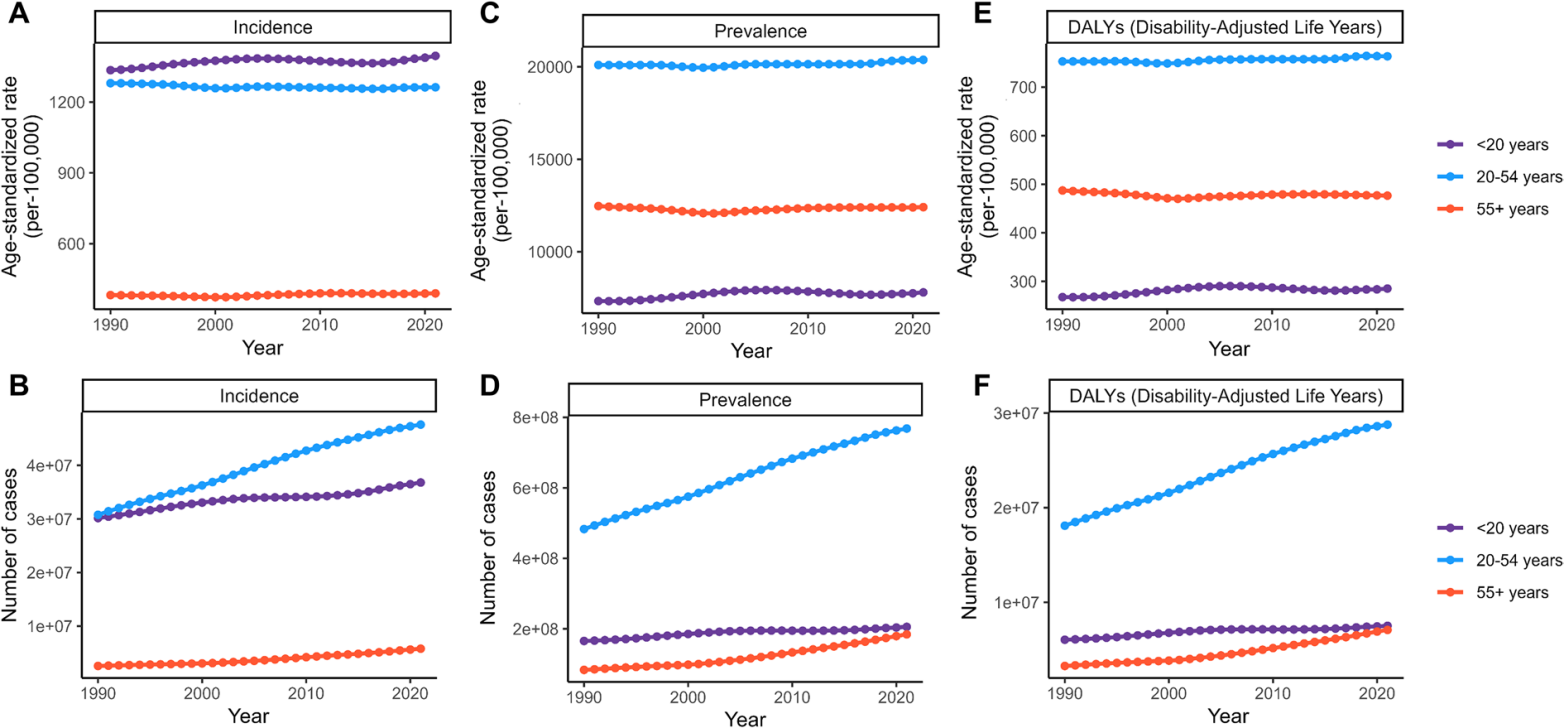
Trend in globally burden of migraine by age groups (< 20, 20–54, > 55) from 1990 to 2021. Trend in the ASIR (**A**) and incidence cases (**B**), ASPR (**C**) and prevalence cases (**D**), and ASDR (**E**) and DALYs cases (**F**) for migraine from 1990 to 2021. *ASDR* age-standardized DALY rate, *ASIR* age-standardized incidence rate, *ASPR* age-standardized prevalence rate, *DALYs* disability-adjusted life years

Since the long-term trends in ASR within the 5-year age groups are not pronounced (Supplementary Material Table S2, Fig. S3), we conducted further analysis using broader age groups: “< 20 years,” “20–54 years,” and “> 55 years.” ASIR for adolescents (under 20) was higher than that of the other two groups, with their ASPR and ASDR showing significantly faster EAPC of 0.17 and 0.18, respectively. In contrast, the other two groups exhibited relatively stable trends, with EAPC ranging from −0.03 to 0.05. In 2010, the young to middle-aged group (20–54) had the highest ASPR at 20,380.33, along with the highest ASDR at 763.55 (Supplementary Material Table S2, Fig. 3).

### Differences by SDI Region

**Fig. 4:**
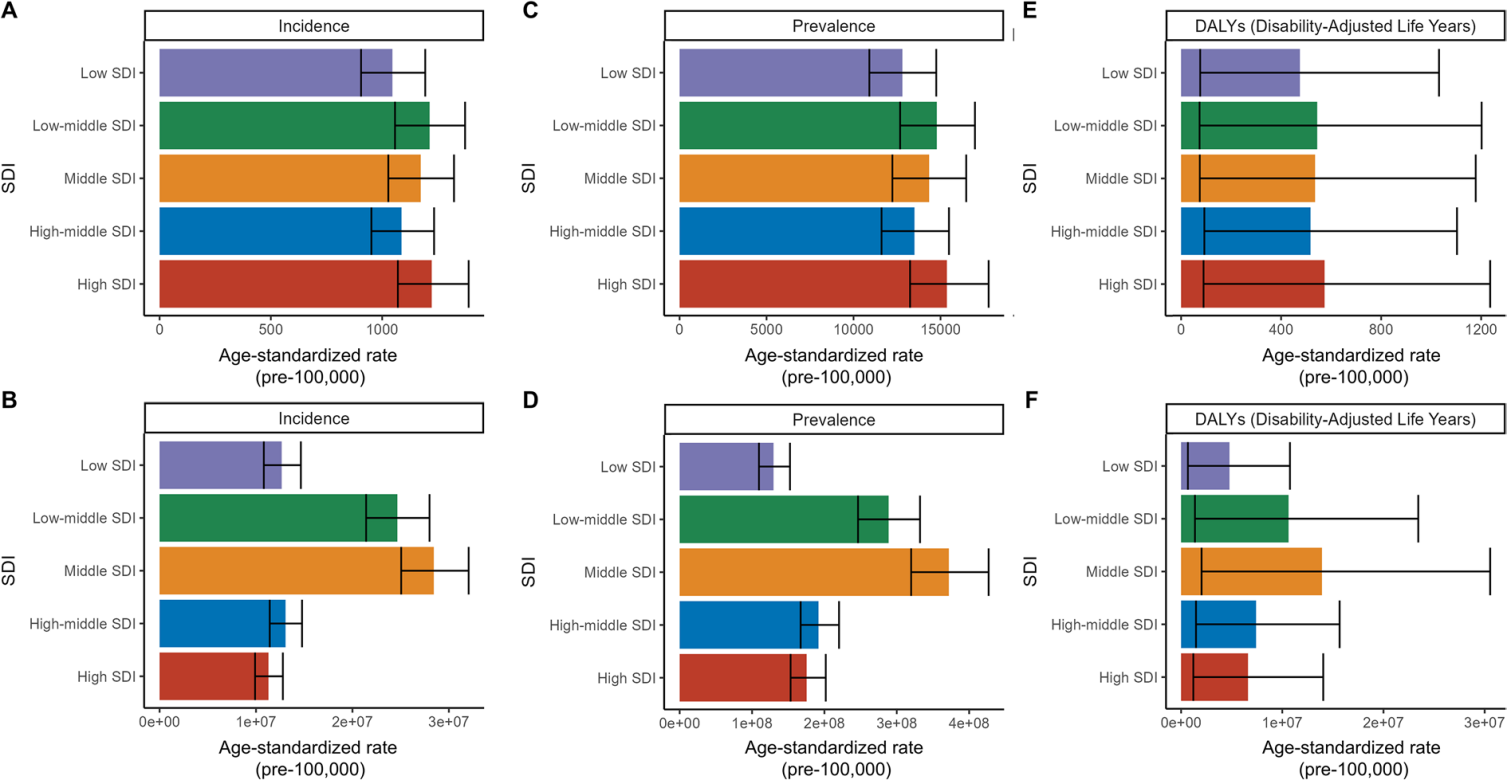
Cross-section (2021) of incidence rate, prevalence rate, and DALY rate for migraine across SDI regions. ASIR (**A**) and incidence cases (**B**), ASPR (**C**) and prevalence cases (**D**), and ASDR (**E**) and DALYs cases (**F**) for migraine. *ASDR* age-standardized DALY rate, ASIR, age-standardized incidence rate, *ASPR* age-standardized prevalence rate, *DALYs* disability-adjusted life years, *SDI* sociodemographic index

From 1990 to 2021, the burden of migraine varied significantly across different SDI regions (Supplementary Material Table S1, Fig. 4, Fig. S4). The ASIR, ASPR, and ASDR in high SDI areas were highest: 1,222.54, 15,365.14, and 573.62, respectively. The ASIR, ASPR, and ASDR in low SDI areas were lowest: 1,045.99, 12,808.97, and 475.21, respectively. However, the medium SDI and low to medium SDI populations are huge, the number of people who experience migraine in these groups includes more than half of the global population, and the resulting disease burden is considerable.

In the medium to high SDI region, both ASIR and ASPR increased, with EAPC of 0.15 and 0.10, respectively, whereas the increase in ASDR was only 0.08. The high SDI region exhibited relatively stable trends, with minimal increases in ASIR and ASPR, EAPC of 0.08 and 0.02, and a negligible change in ASDR, EAPC of 0.02. Conversely, the low to medium SDI region showed slight declines in both ASIR and ASPR, with EAPCs of −0.03 and −0.02. In the low SDI region, incidence and prevalence were almost unchanged, whereas ASDR exhibited a slight increase, with an EAPC of 0.03. The medium SDI region showed the most significant increases, with ASIR and ASPR exhibiting EAPCs of 0.16 and 0.19, respectively, highlighting a marked increase in migraine burden (Supplementary Material Table S1, Fig. 4).

### Differences by Region and Country

The global burden of migraine exhibited considerable variation across different regions (Supplementary Material Table S3). Increases were most significant in East Asia and Latin America, with an EAPC of 0.28 for prevalence and 0.26 for DALYs. In Andean Latin America, the EAPCs for prevalence and DALYs were 0.23 and 0.21, respectively; Southern and Tropical Latin America also demonstrated notable upward trends. The most significant decrease in EAPC was observed in Southeast Asia, with a prevalence rate of −0.07, and the EAPC for DALYs was −0.06. However, despite increases in absolute migraine cases in other regions, the incidence and prevalence rate EAPC remained relatively stable here.

**Fig. 5:**
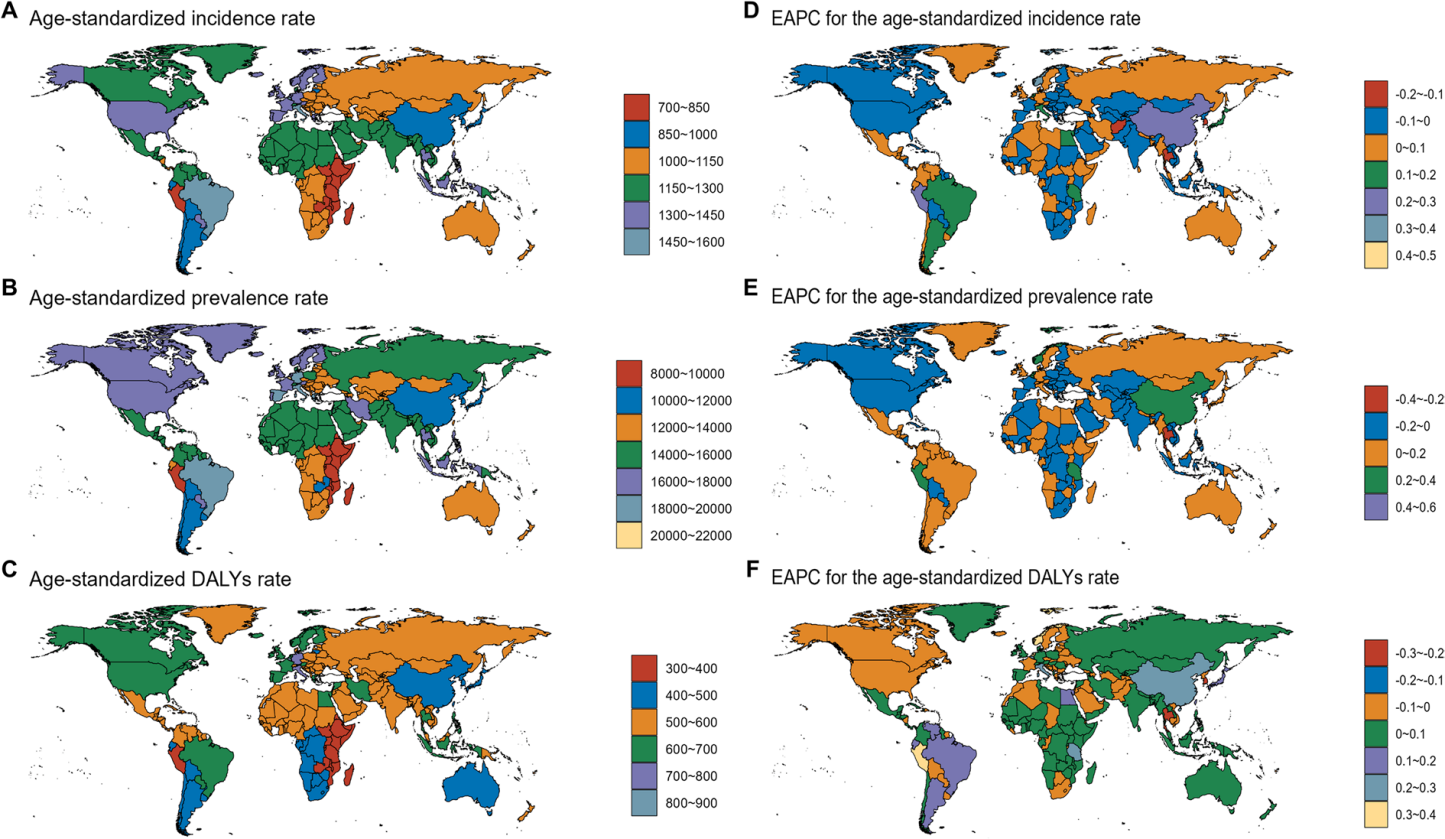
National age-standardized incidence, prevalence, and DALY rates for migraine in 2021, with EAPC from 1990 to 2021. ASIR (**A**), ASPR (**B**), and ASDR (**C**). EAPC of ASIR (**D**), ASPR (**E**), and ASDR (**F**) from 1990 to 2021. *ASDR* age-standardized DALY rate, *ASIR* age-standardized incidence rate, *ASPR* age-standardized prevalence rate, *DALYs* disability-adjusted life years, *EAPC* estimated annual percentage changes

In terms of countries (Supplementary Material Table S4, Fig. 5), incidence rate, prevalence, and DALYs increased most in Singapore, with EAPCs of 0.41, 0.48, and 0.4, respectively. Incidence decreased most in South Korea, with an EAPC of −0.18, whereas prevalence and DALYs decreased most in Thailand, with EAPCs of −0.31 and −0.29, respectively. Brazil had the highest ASIR (1510.77) but Belgium had the highest ASPR (21,751.47) and ASDR (800.36). Detailed data are provided in Supplementary Material Table S4.

### Correlation Analysis

**Fig. 6:**
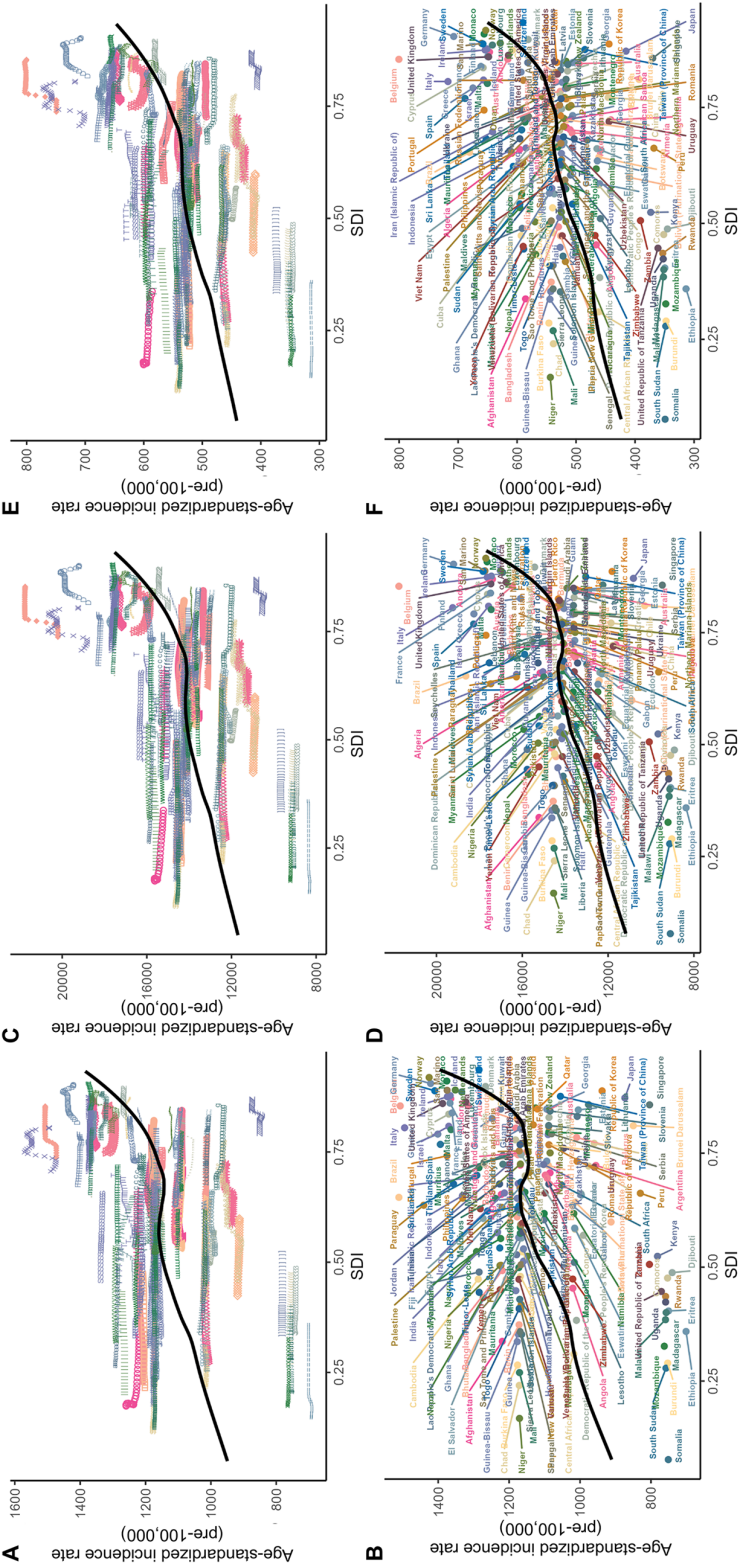
The correlation between SDI and ASR of migraine in 2021. ASIR (**A**), ASPR (**B**), and ASDR (**C**). *ASDR* age-standardized DALY rate, *ASIR* age-standardized incidence rate, *ASPR* age-standardized prevalence rate, *ASR* age-standardized rate, *SDI* sociodemographic index

As the SDI increased, there was a general trend for increasing migraine ASIR, ASPR, and ASDR (Fig. 6). This upward trend is relatively stable for SDI values of 0.5–0.7 but increases considerably above a value of 0.7, suggesting that countries with high SDI tend to report higher migraine burdens. The ASIR, ASPR, and ASDR of migraine is not significantly related to its EAPC (*P* > 0.05, Supplementary Material Fig. S5A–5C). Fig. S5D–5E shows that the human development index (HDI) is positively correlated with the EAPC of ASIR and ASPR of migraine (*P* < 0.01, *ρ* = 0.20 and *P* = 0.02, *ρ* = 0.19), but not significantly correlated with ASDR (*P* = 0.14, Fig. S5F).

### Frontier Analysis

**Fig. 7:**
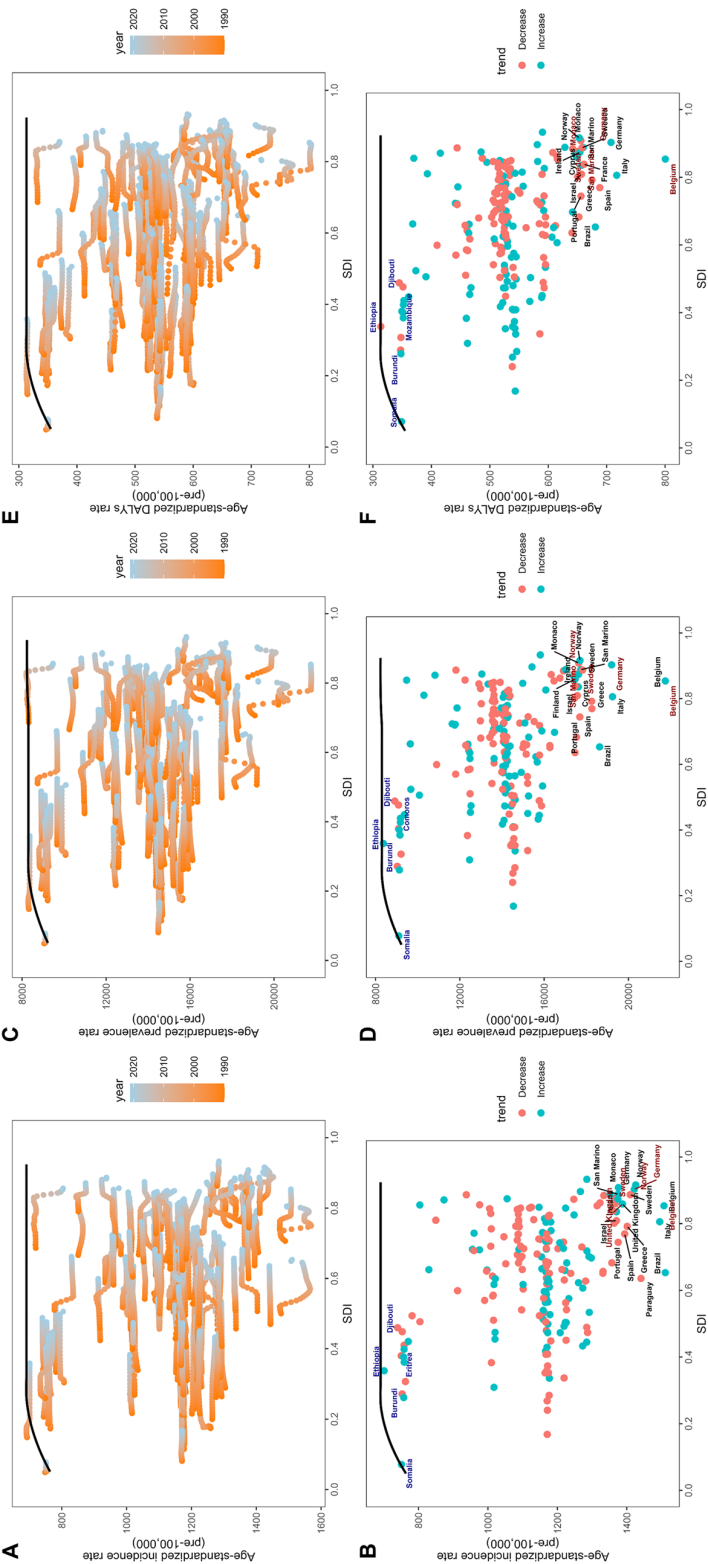
Frontier analysis of migraine in 204 countries and regions in 2021. The gap in ASIR (**A**, **B**), ASPR (**C**, **D**), and ASDR (**E**, **F**) between different countries and the frontier. The frontier represents the ideal level of control. The top 15 countries furthest from the frontier are marked in black; countries with low SDI (< 0.5) and nearest to the frontier are marked in blue; countries with high SDI (> 0.85) and furthest from the frontier are marked in red. Red dots indicate an increase in ASR from 1990 to 2021; blue dots indicate a decrease in ASR from 1990 to 2021. *ASDR* age-standardized DALY rate, *ASIR* age-standardized incidence rate, *ASPR* age-standardized prevalence rate, *SDI* sociodemographic index

A comprehensive frontier analysis of different countries and regions of migraine from 1990 to 2021 exhibit marked heterogeneity in the prevention and control. In theory, the burden of migraine should exhibit an downward trend with increasing SDI, and the frontier remained steady after the SDI exceeded 0.25; death rate and DALYs should also followed a similar pattern. The visual representations revealed marked differences among countries. A total of 15 countries (including Brazil, Belgium, Italy, Paraguay, and Norway) had significantly higher prevalence rates and were far from the frontier. Conversely, countries with low SDI (< 0.5), such as Comoros, Burundi, Djibouti, Ethiopia, and Somalia were closer to the frontier, indicating more optimal outcomes relative to their SDI. Countries with high SDI (> 0.85) such as Belgium, Germany, Sweden, San Marino, and Norway exhibited larger deviations from the frontier, indicating a heavier burden. In the DALYs analysis, countries such as Belgium, Italy, and Germany exhibited large deviations from the frontier. Detailed frontier analyses of ASIR, ASPR, and ASDR across countries are presented in Fig. 7 and Supplementary Material Table S5.

### Predictive Analysis for 2050

**Fig. 8:**
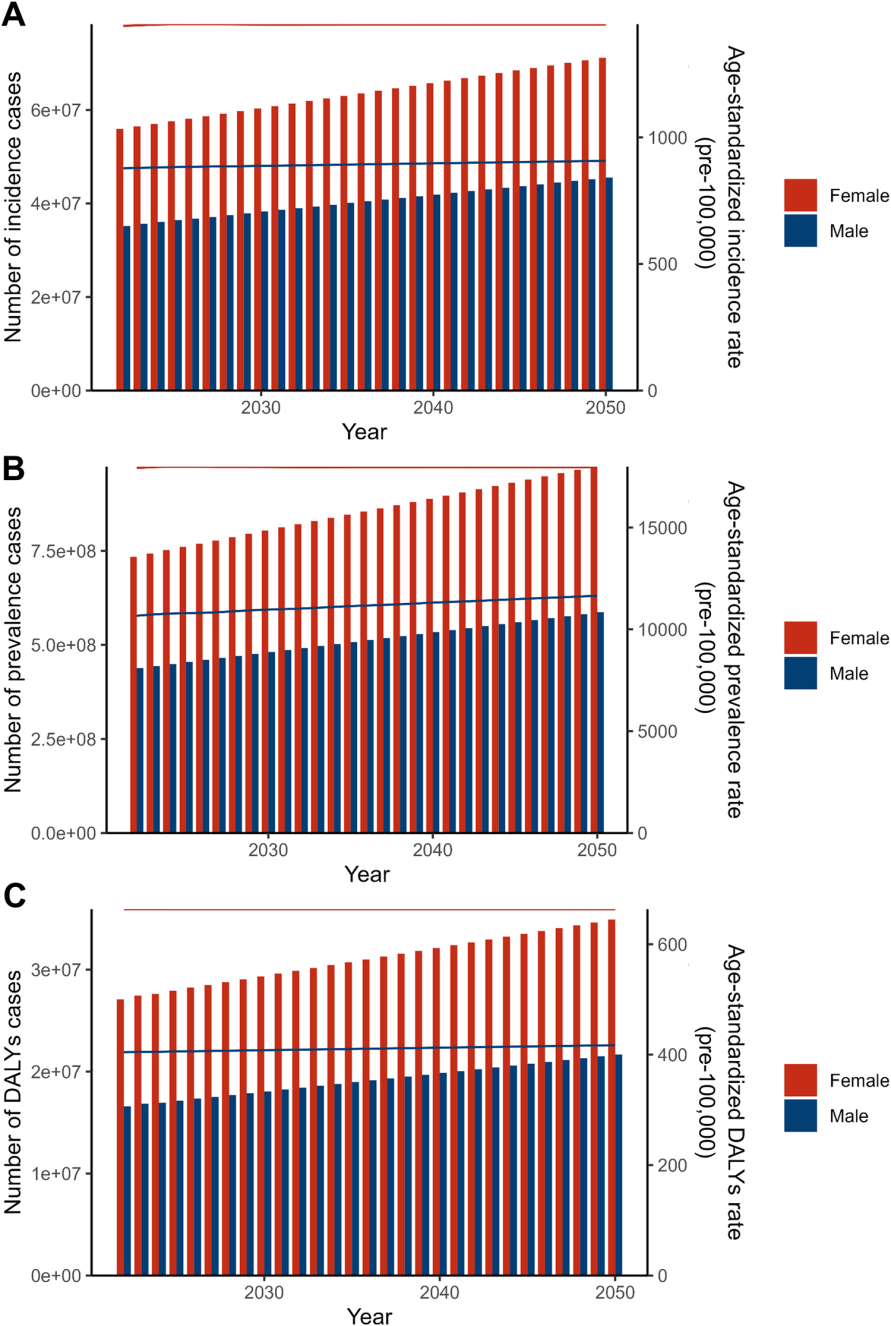
Trends in observed and predicted global burden of migraine by sex from 2021 to 2050. **A** The number of incidence cases and its ASIR from 2021 to 2050; **B** the number of prevalence cases and its ASPR from 1990 to 2050; **C** the number of DALYs cases and its ASDR from 2021 to 2050. *ASDR* age-standardized DALY rate, *ASIR* age-standardized incidence rate, *ASPR* age-standardized prevalence rate, *DALYs* disability-adjusted life years

Projections for migraine burden from 2022 to 2050 reveal a notable disparity in growth rates between the sexes, with male individuals expected to experience a more rapid increase than female individuals (Supplementary Material Table S6, Fig. 8). By 2050, the prevalence of male patients is expected to reach 586.96 million, with the number of female patients expected to reach 973.71 million (Fig. 8B). The ASIR for male individuals is expected to increase from 876.55 in 2021 to 907.55 per 100,000 people in 2050, an increase of 3.53%, whereas the ASIR for female individuals is expected to increase only slightly, from 1438.9 to 1445.75 per 100,000 people, an increase of 0.47% (Fig. 8A). Moreover, the growth patterns in ASPR and ASDR are similar: the ASPR for male individuals is expected to rise from 10,624.2 in 2021 to 11,652.14 in 2050 per 100,000 people, an increase of 9.68%, and the ASDR for male individuals is expected to rise from 403.88 in 2021 to 421.77 in 2050 per 100,000 people, an increase of 4.43% (Fig. 8C). The corresponding increases in ASPR and ASDR for female individuals are expected to be much smaller (Fig. 8A, B), with ASPR increasing by 0.36% from 17,902.6 in 2021 to 17,967.77 in 2050 per 100,000 people and ASDR increasing by 0.11% from 662.76 in 2021 to 663.46 in 2050 per 100,000 people (Fig. 8C). Regardless of the ASIR, ASPR, or ASDR, the trend of migraine has significantly increased more among male individuals than among female individuals.

**Fig. 9:**
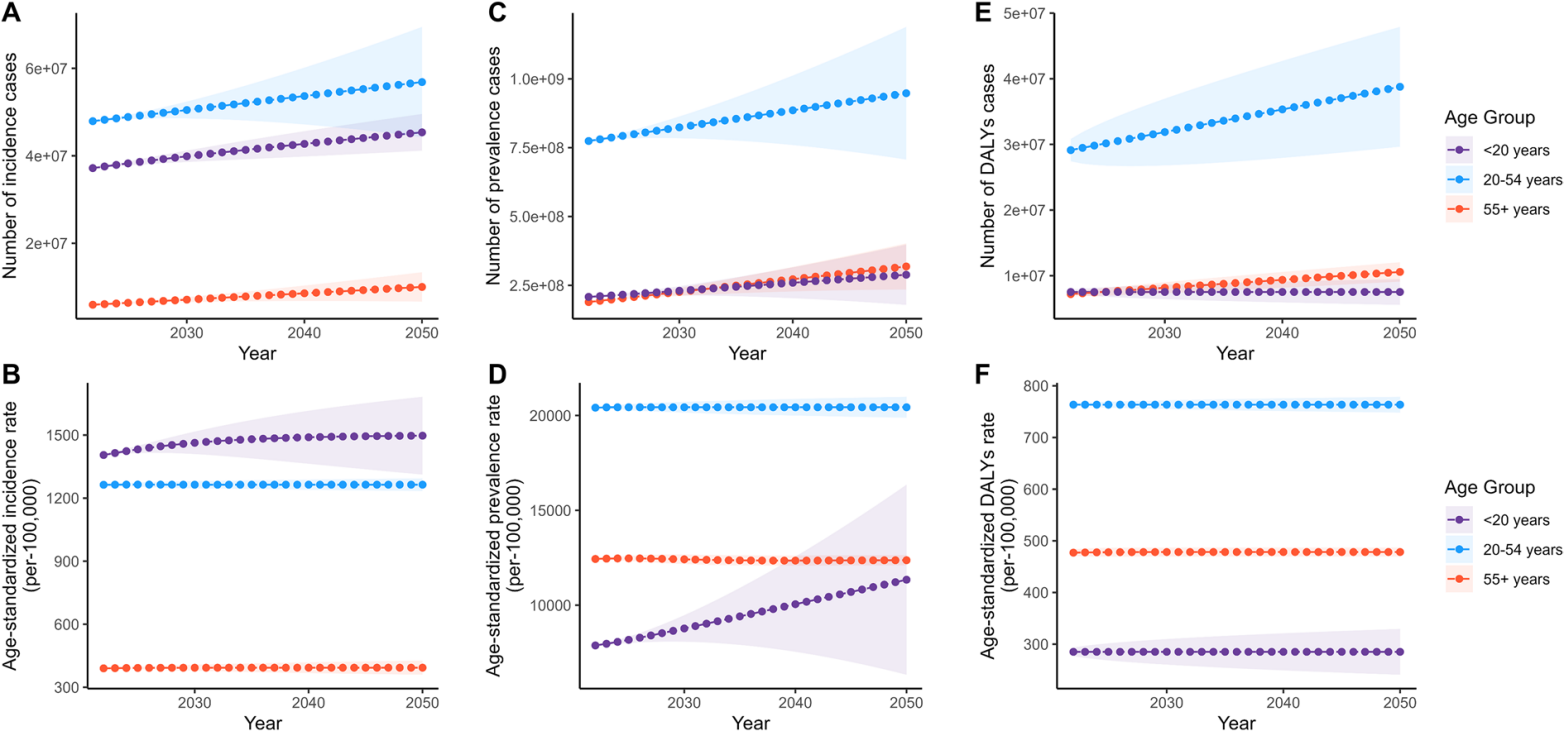
Trends in observed and predicted of global burden of migraine by age groups (< 20, 20–54, > 55) from 2021 to 2050. **A** The number of incidence cases and its ASIR for age groups from 2021 to 2050; **B** the number of prevalence cases and its ASPR for age groups from 1990 to 2050; **C** the number of DALYs cases and its ASDR for age groups from 2021 to 2050. *ASDR* age-standardized DALY rate, *ASIR* age-standardized incidence rate, *ASPR* age-standardized prevalence rate, *DALYs* disability-adjusted life years

The predictive analysis by age group highlights significant changes in ASIR and ASPR for adolescents (< 20 years) with migraine (Supplementary Material Table S6, Fig. 9). The ASIR is expected to increase from 1395.94 per 100,000 people in 2021 to 1497.09 in 2050 (Fig. 9B), representing a growth of 7.25%. During the same period, the ASPR is projected to rise from 7805.07 to 11,340.41 per 100,000 people (Fig. 9D), an increase of 45.30%. The ASDR across all age groups is expected to remain relatively stable.

## Discussion

This study provides a comprehensive analysis of the global burden of migraine from 1990 to 2021, assessing incidence, prevalence, and DALYs across age groups and sexes. Previous research has often focused on female populations or specific age groups, overlooking broader trends. Our findings indicate that while female individuals bear a higher overall migraine burden, male individuals have experienced a more rapid increase in prevalence. The prevalence and DALYs for adolescents (< 20 years) are also expected to rise significantly, highlighting the need for targeted public health attention and interventions.

Over the past 32 years, the global burden of migraine has escalated. There are substantial disparities in the burden of migraine across regions, sexes, and SDI regions. Despite significant advances in medical technology and treatments over the past few decades, the burden of migraine as a global public health issue remains heavy. This may be due to factors such as population growth, aging, accelerated urbanization, and lifestyle changes [31–33]. Consequently, migraine represents a significant challenge to public health, and policymakers must prioritize prevention and management of migraine to mitigate its socioeconomic impact.

The epidemiology of migraine is characterized by a significant disparity between the sexes, with female individuals experiencing a substantially higher burden than male individuals. The mechanisms underlying this discrepancy are complex and may involve various physiological and psychological factors, such as hormonal fluctuations, pain sensitivity, and environmental stressors [34, 35]. Female individuals tend to have greater sensitivity to pain and lower pain tolerance [36]. Estrogen level fluctuations are often considered a significant trigger for migraine [13, 37], with incidence rates rising sharply during puberty, peaking during earlier adulthood, and declining after menopause. Although the absolute burden of migraine remains significantly higher among female individuals, the rates of incidence, prevalence, and DALYs are increasing more rapidly among male individuals. This may reflect escalating health challenges faced by male individuals in modern society, such as intense physical labor, increased work stress, and a poorer awareness of individual health management [38, 39]. Additionally, barriers to reporting and treating migraine among male individuals—such as traditional sex/gender roles and increased pain tolerance—may lead to underdiagnosis and undertreatment [34, 37, 40, 41]. The decreased awareness and fewer migraine consultations may exacerbate the health burden among male individuals [6–8]. Consequently, future public health strategies should equally focus on the male population, particularly regarding disease prevention and early intervention.

Second, the burden of migraine remains considerable among adolescents and the middle-aged [16, 25, 34, 42]. The incidence of migraine peaks in the 10–14 years age group, while prevalence and DALYs start to increase in this age group and peak in the 40–44 years age group. The absolute numbers for migraine prevalence and DALYs are greatest in the very large 30–34 years age group. This period coincides with the peak of many individuals’ career and family responsibilities. Broader age-group analysis reveals that adolescents (< 20 years) exhibit a notably higher ASIR than other groups, along with faster growth rates in ASPR and ASDR. Predictive models further suggest that ASIR and ASPR on adolescents are expected to increase significantly. These trends underscore the need for targeted preventive measures for adolescents, including early intervention and education on lifestyle factors such as screen time, sleep hygiene, and stress management, which may help curb the rising incidence of migraine in this group [21]. For adolescents, prevention and early identification of migraine onset should be focal points. For adults, more effective management strategies are needed, such as optimizing work environments, providing psychological support, and promoting healthy lifestyles to mitigate the negative impact of migraine on work and life. In middle age, factors such as occupational stress, family responsibilities, and lifestyle choices may increase migraine prevalence and DALYs to their highest levels [43]. Therefore, prevention and treatment strategies need to be targeted to different age groups.

The burden of migraine has significantly increased in medium SDI and medium to high SDI countries. This trend may be closely related to changes in lifestyle, accelerated industrialization, and increased mental stress in these nations. Although countries with high SDI have remained relatively stable, the burden of migraine in these countries remains considerable. In contrast, the increase, or even decrease, in migraine burden in regions with low and low to medium SDI is less pronounced; this may be due to poorer healthcare resources, lower health awareness, and reduced diagnostic rates. It is crucial to develop public health strategies tailored to address the needs of regions with different SDI to effectively manage the global rise in migraine burden. Additionally, our analysis of the correlation between SDI and migraine burden suggests that although socioeconomic development brings numerous health benefits, it may also induce stress and lifestyle changes that exacerbate migraine. The study found that as a country progresses socioeconomically, the burden of migraine tends to increase, particularly after the SDI surpasses 0.7. Although countries with high SDI have better medical resources and living conditions, they also have a greater migraine burden; this may be due to higher stress levels, longer working hours, and/or more comprehensive disease diagnostic systems.

A frontier analysis revealed further disparities among countries in addressing the burden of migraine. In theory, As SDI increased from 0.0 to 1.0, the burden of migraine should decrease, with the frontier stabilizing after the SDI exceeded 0.25. Countries with high SDI, such as Belgium, Italy, and Germany exhibited a migraine burden that was significantly above the frontier level, indicating that despite ample resources, these countries have not yet achieved optimal migraine management and there is still considerable room for improvement. These countries need to optimize their public health strategies, particularly regarding disease prevention, early interventions, and personalized treatment. In contrast, countries such as Comoros, Burundi, Djibouti, Ethiopia, and Somalia, which have relatively low SDI, achieve better outcomes in managing migraine. Countries far from the frontier line should carefully investigate the underlying causes and implement targeted interventions to alleviate the burden of migraine.

Overall, these findings emphasize the importance of tailoring public health strategies to the sociodemographic contexts of different regions. Where countries with high SDI need to focus on improving the effectiveness of migraine management and addressing the psychosocial factors that exacerbate disease burden, countries with low SDI should prioritize increasing healthcare access, diagnostic capabilities, and awareness about migraine. By adopting a targeted approach that considers the unique challenges and resources of each SDI region, we can reduce the global burden of migraine and improve the quality of life for those affected by the condition.

Globally, the burden of migraine exhibits significant fluctuations across different regions, reflecting vast disparities in socioeconomic status, healthcare resources, and lifestyle. The burden of migraine has considerably increased in East Asia and the Andean regions of Latin America. The southern and tropical regions of South America also show significant upward trends, possibly due to rapid urbanization, lifestyle changes, inadequate migraine management, and prevention measures [31, 44, 45]. By contrast, Southeast Asia apparently exhibits a significant downward trend in migraine burden, which may be due to poorer healthcare standards and lower diagnostic rates rather than more effective prevention and treatment measures. At the national level, Singapore has significantly greater prevalence of migraine and EAPC of DALYs than other countries, indicating that it has the fastest growing migraine burden. Despite its advanced healthcare resources, the city-state of Singapore faces a rapidly increasing migraine burden, perhaps due to its competitive culture and high levels of stress. Research suggests that primary care physicians in Singapore face major challenges in continuously diagnosing migraine, and preventive treatments remain underutilized [46], with more individuals seeking treatment for headaches [47]. Conversely, South Korea has the lowest EAPC in migraine incidence, perhaps reflecting significant progress in migraine management. The EAPCs in migraine prevalence and DALYs are lowest in Thailand, and the migraine burden in Thailand has significantly decreased, although it may be currently underestimated. Improving healthcare services and diagnostic capabilities should be prioritized here. Brazil and Belgium currently have the highest recorded standardized incidence and prevalence rates of migraine globally.

With the introduction of calcitonin gene-related peptide (CGRP) therapy, migraine management has entered a new era of opportunity. These inhibitors significantly reduce the frequency and severity of attacks by targeting the underlying mechanisms of migraine [48], offering new hope for patients who have long suffered from this condition, especially among women, where they effectively alleviate migraines triggered by hormonal fluctuations [49]. However, the burden of migraines in male patients is on the rise, with more rapid increases in incidence, prevalence, and DALYs among male individuals than among female individuals until 2050. The potential impact of increasing migraine burden among male individuals may be exacerbated by increasing socioeconomic status, lifestyle changes, and job-related stress. The slower growth in migraine burden among female individuals suggests that current interventions may be more effective. The more rapid increase in migraine burden among male individuals indicates that the traditional view of migraine as a “female disease” may need to be reconsidered. Additionally, adolescents represent another high-risk group, with their ASPR expected to increase significantly by 45.30%. Exploring the reasons behind the rising prevalence of migraines in this group, as well as the safety and efficacy of CGRP therapy in adolescents, is crucial. As CGRP therapy becomes more widespread, the global burden of migraines is expected to change, profoundly impacting management strategies across different age groups. Therefore, future research should focus more on comparative trends between different age groups and genders, as well as the development of effective intervention measures and policies tailored to diverse populations.

## Limitations

This study has several limitations. The GBD utilizes a wide range of data sources, including those from the World Health Organization, disease registries, and surveys, and employs rigorous data validation and cross-validation techniques to address discrepancies. Nevertheless, despite its comprehensive nature, GBD data may still introduce bias, particularly in low SDI regions where healthcare infrastructure is limited, which may result in under-reporting. For migraines, although the GBD integrates data from 506 sources, in regions with limited direct data availability, it relies on statistical modeling approaches, such as DisMod MR 2.1 and spatiotemporal Gaussian process regression (ST-GPR), and applies 95% uncertainty intervals (UI) to quantify uncertainty in the estimates [19]. While these models help address data gaps, estimates from these regions may still encounter challenges related to accuracy. Furthermore, cultural differences in the perception and reporting of pain could introduce additional biases in the data. Future research should focus on improving data collection methods, particularly in underrepresented regions.

## Conclusions

From 1990 to 2021, the global burden of migraine increased significantly. Although the overall burden of women will be higher, the prevalence and incidence rate of men increased faster—possibly driven by lifestyle changes or improved diagnosis. Historically, these male trends have been overlooked. The growing burden among male individuals requires prioritized intervention strategies, including early diagnosis and targeted health education. In addition, the significant increase in the prevalence of adolescents (< 20 years) also deserves our attention. Countries should develop interventions and allocate resources based on regional needs to mitigate the increasing migraine burden effectively. Future research and policy should continue to prioritize this global health challenge to ensure effective resource allocation and mitigation of the migraine burden through appropriate interventions.

## Supplementary Information

Below is the link to the electronic supplementary material.

Supplementary file 1 (PDF 2275 KB)

## Data Availability

All original data are accessible through the Global Burden of Disease Study 2021 at: https://www.healthdata.org/
