# Supplementary material for: The Global Burden of Migraine: A 30-Year Trend Review and Future Projections by Age, Sex, Country, and Region
Source: Pain Ther. 2024 Dec 11;14(1):297–315. doi: 10.1007/s40122-024-00690-7 (PMC11751287; doi:10.1007/s40122-024-00690-7)
Supplement: Supplementary file 1 — (PDF 2275 KB) [file 40122_2024_690_MOESM1_ESM.pdf]

Supplementary Material

Title: The Global Burden of Migraine: A 30-Year Trend Review and Future Projections by Age, Sex, Country, and Region

Authors and affiliations:

Lingkang Dong[1†], Wenqi Dong[1†], Yuchen Jin[1†], Yumeng Jiang[1*], Zhuangzhuang Li[2*], Dongzhen Yu[1*]

[1] Department of Otolaryngology Head & Neck Surgery, Shanghai Sixth People's Hospital Affiliated to Shanghai Jiao Tong University School of Medicine, Shanghai, China

[2]Department of Otolaryngology, Sun Yat-sen Memorial Hospital, Sun Yat-sen University, Guangzhou, China

[†]Lingkang Dong, Wenqi Dong and Yuchen Jin contributed equally.

*Correspondence to Dongzhen Yu, Zhuangzhuang Li and Yumeng Jiang.

Dongzhen Yu

Email: 7250012023@shsmu.edu.cn

Zhuangzhuang Li

Email: drzhuangzhuang@163.com

Yumeng Jiang

Email: jianglem@126.com.

Supplementary Material data

Supplementary Fig. S1 Trend in global burden of migraine from 1990 to 2021

Supplementary Fig. S2 Global burden of migraine by sex, 2021.

Supplementary Fig. S3 Trend in global burden of migraine by 5-year age groups from 1990 to 2021.

Supplementary Fig. S4 Trend in global burden of migraine by SDI regions from 1990 to 2021.

Supplementary Fig. S5 Correlation between HDI, ASR and EAPC of migraine, 2021.

Supplementary Table S1 Global burden of incidence, prevalence, and DALYs of migraine trends from 1990 to 2021 by sex, 5 SDI regions

Supplementary Table S2 Global burden of incidence, prevalence, and DALYs of migraine trends from 1990 to 2021 by age groups.

Supplementary Table S3 Global burden of incidence, prevalence, and DALYs of migraine trends from 1990 to 2021 by 21 GBD regions.

Supplementary Table S4 Global burden of incidence, prevalence, and DALYs of migraine trends from 1990 to 2021 by countries and regions**.**

Supplementary Table S5 Frontier incidence, prevalence, DALYs, and effective differences by countries and regions, 2021.

Supplementary Table S6 Predictive analysis of incidence, prevalence, DALYs of migraine in males and females from 2022 to 2050

Supplementary Table S7 Predictive analysis of incidence, prevalence, and DALYs of migraine across age groups '<20,' '20-54,' and '>55' from 2022 to 2050

Supplementary Fig. S1 Trend in global burden of migraine from 1990 to 2021. (**A**) Trend in the incidence cases and ASIR; (**B**) Trend in the prevalence cases and ASPR; (**C**) Trend in the DALYs cases and ASDR.

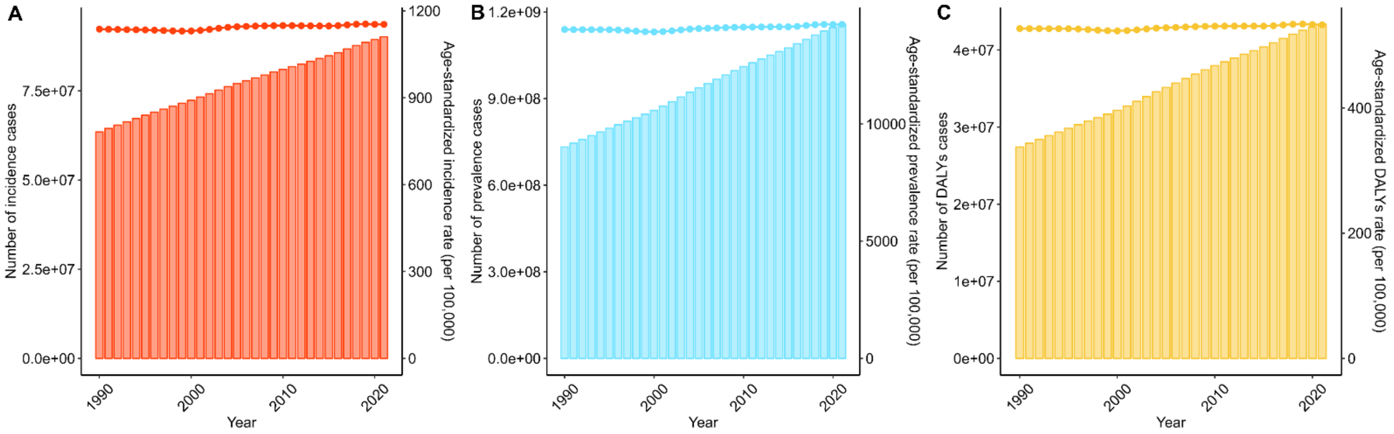

Supplementary Fig. S2 Global burden of migraine by sex, 2021. ASIR (**A**) and incidence cases (**B**), ASPR (**C**) and prevalence cases (**D**), ASDR (**E**) and DALYs cases (**F**) for migraine in 2021.

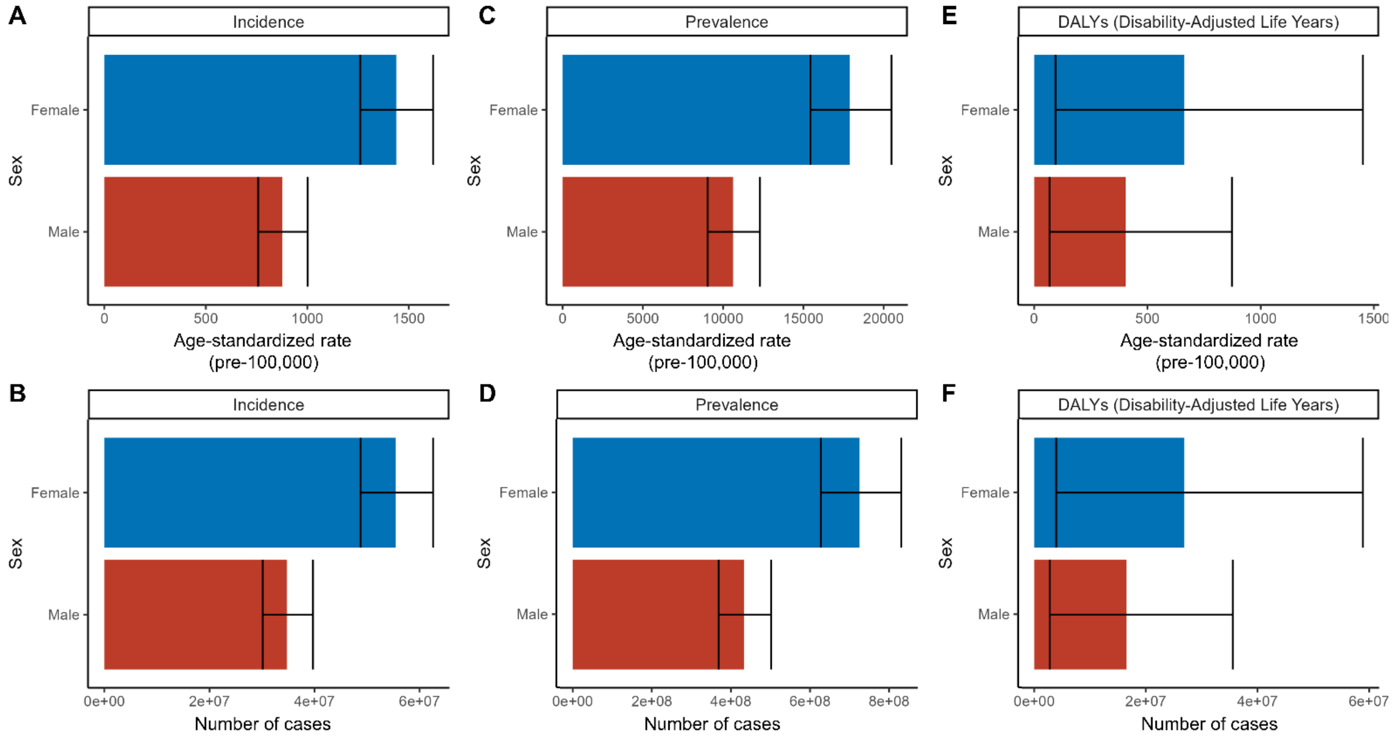

Supplementary Fig. S3 Trend in global burden of migraine by 5-year age groups from 1990 to 2021. Trend in the ASIR (**A**) and incidence cases (**B**), ASPR (**C**) and prevalence cases (**D**), ASDR (**E**) and DALYs cases

(**F**) for migraine from 1990 to 2021.

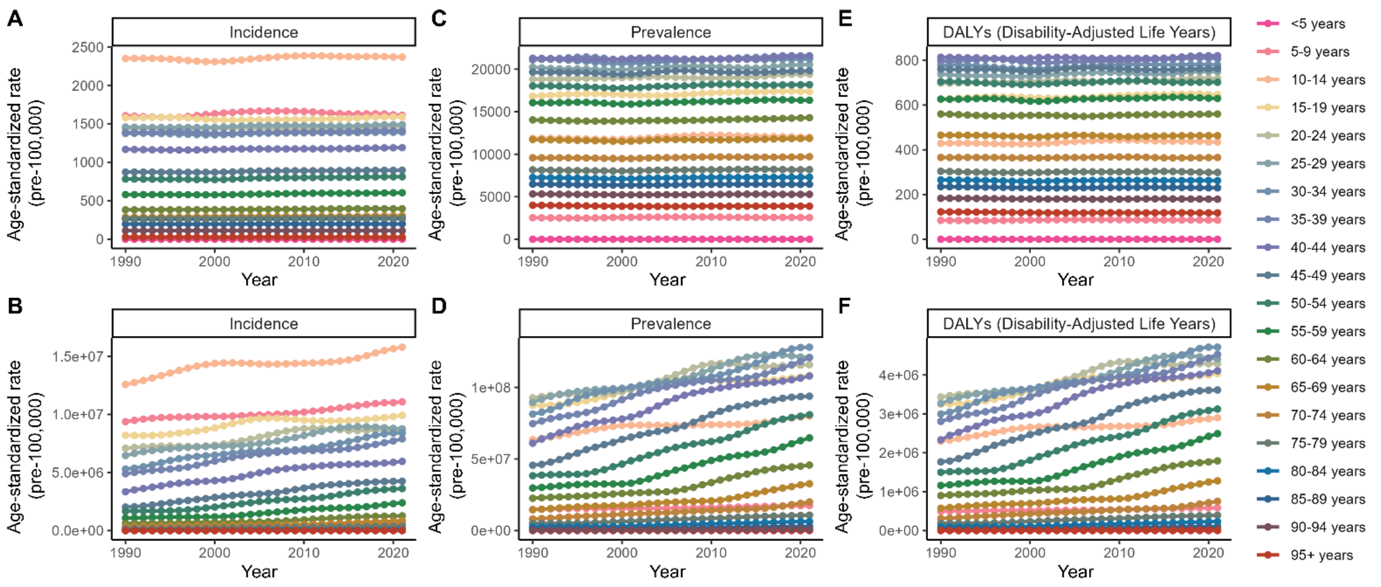

Supplementary Fig. S4 Trend in global burden of migraine by SDI regions from 1990 to 2021. Trend in the ASIR (**A**) and incidence cases (**B**), ASPR (**C**) and prevalence cases (**D**), ASDR (**E**) and DALYs cases (**F**) for migraine from 1990 to 2021.

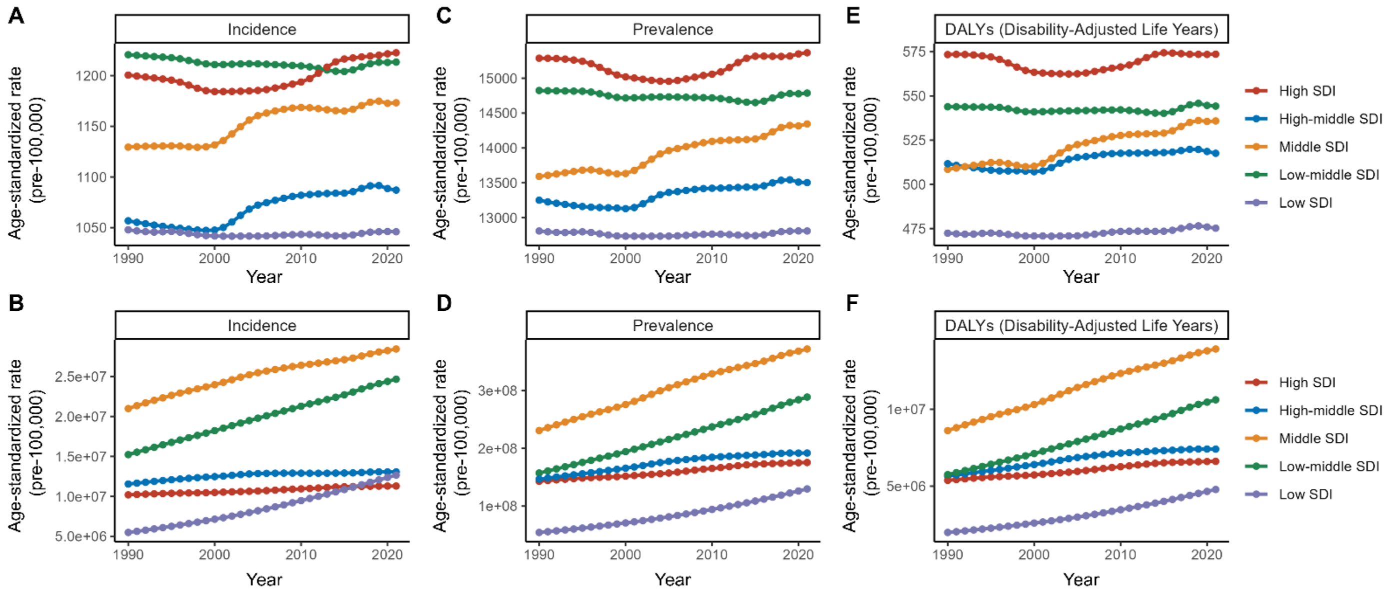

Supplementary Fig. S5 Correlation between HDI, ASR and EAPC of migraine. (**A-C**) Correlation between ASR and EAPC; (**D-F**) Correlation between HDI and EAPC.

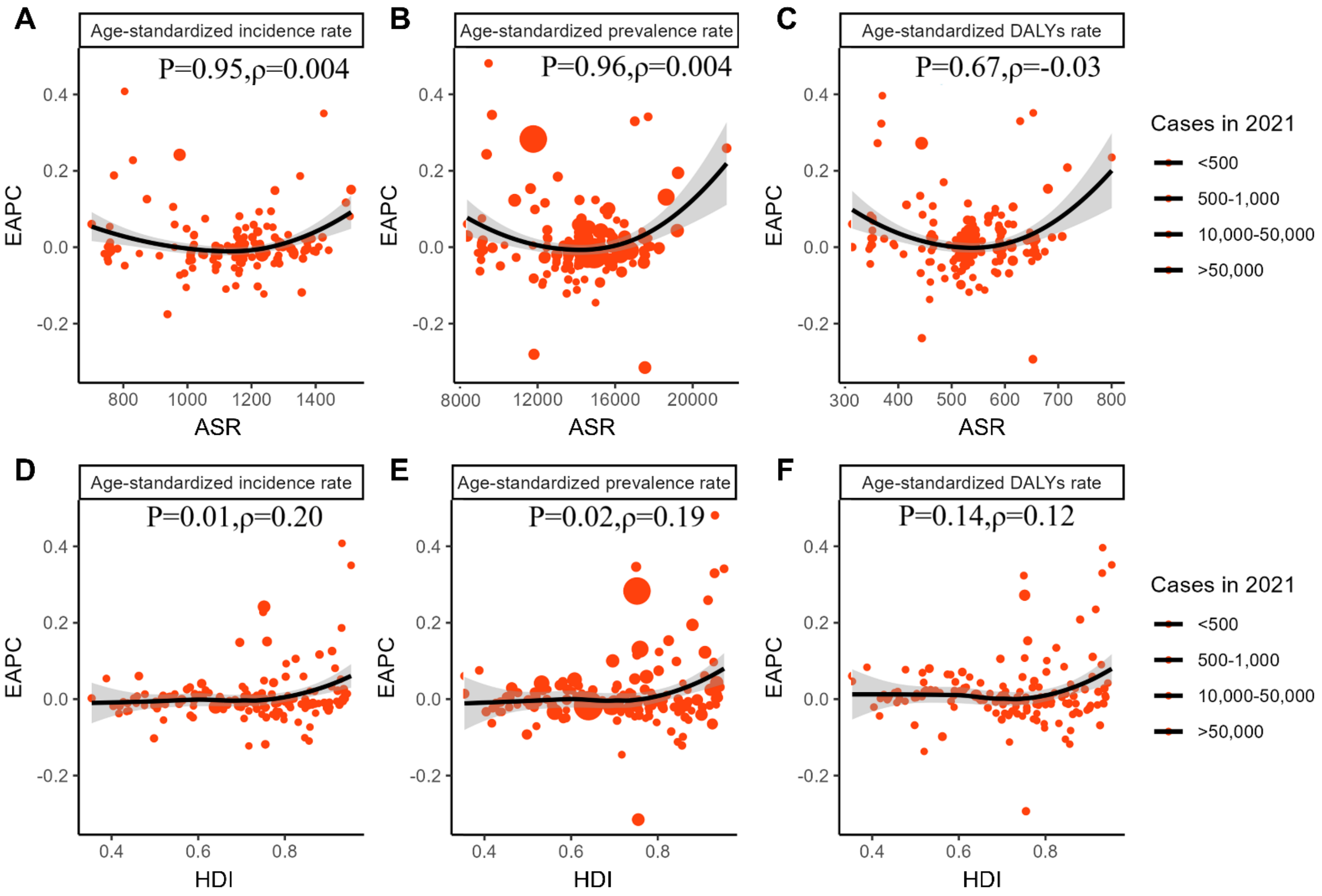

A
Age-standardized incidence rate
P=0.95,ρ=0.004
EAPC
ASR
B
Age-standardized prevalence rate
P=0.96,ρ=0.004
EAPC
ASR
C
Age-standardized DALYs rate
P=0.67,ρ=-0.03
EAPC
ASR
Cases in 2021
<500
500-1,000
10,000-50,000
>50,000
D
Age-standardized incidence rate
P=0.01,ρ=0.20
EAPC
HDI
E
Age-standardized prevalence rate
P=0.02,ρ=0.19
EAPC
HDI
F
Age-standardized DALYs rate
P=0.14,ρ=0.12
EAPC
HDI
Cases in 2021
<500
500-1,000
10,000-50,000
>50,000

Supplementary Table S1 Global burden of incidence, prevalence, and DALYs of migraine trends from 1990 to 2021 by sex, 5 SDI regions. ASIR, age-standardized incidence rate; ASPR, age-standardized prevalence rate; DALYs, disability-adjusted life years; ASDR, age-standardized DALY rate; EAPC, estimated annual percentage changes; SDI, sociodemographic index.

	Incidence					Prevalence					DALYs				
	1990		2021			1990		2021			1990		2021		
	Cases	ASIR	Cases	ASIR	EAPC	Cases	ASPR	Cases	ASPR	EAPC	Cases	ASDR	Cases	ASDR	EAPC
Characteristics	(95%UI)	pre-100,000 (95%UI)	(95%UI)	pre-100,000 (95%UI)	(95%CI)	(95%UI)	pre-100,000 (95%UI)	(95%UI)	pre-100,000 (95%UI)	(95%CI)	(95%UI)	pre 100,000 (95%UI)	(95%UI)	pre-100,000 (95%UI)	(95%CI)
Global	63496591 (55194751-72208003)	1136.9 (995.14-1287.76)	90183387 (78857600-101838162)	1153.2 (1006.07-1304.49)	0.07 (0.06-0.08)	732564463 (624559244-847058436)	14027.65 (12063.37-16078.07)	1158432824 (995861966-1331312506)	14246.55 (12194.12-16378.7)	0.06 (0.05-0.07)	27412196 (4076605-60325806)	526.76 (83.36-1145.92)	43378890 (6732642-95079454)	532.7 (80.57-1167.71)	0.05 (0.04-0.07)
Sex															
Female	39504167 (34479511-44792382)	1435.6 (1257.48-1617.92)	55429677 (48751163-62552034)	1438.9 (1261.41-1620.05)	0.03 (0.02-0.04)	462896609 (396950116-532406330)	17864.62 (15368.19-20418.65)	725242192 (627617458-830850947)	17902.6 (15445.99-20487.01)	0.02 (0.01-0.03)	17179628 (2400080-37859718)	664.92 (98.1-1453.28)	26883943 (3930571-58848029)	662.76 (93.66-1450.83)	0.01 (0-0.02)
Male	23992424 (20793827-27423189)	846.91 (735.94-963.38)	34753710 (30126608-39711731)	876.55 (758.19-1002.54)	0.13 (0.11-0.14)	269667853 (227787523-312882001)	10229.25 (8743.81-11822.93)	433190632 (368788829-501602102)	10624.2 (9039.46-12297.27)	0.13 (0.11-0.15)	10232569 (1675318-22688369)	389.98 (68.44-844.94)	16494946 (2802072-35551602)	403.88 (67.39-872.77)	0.13 (0.11-0.14)
SDI region															
High-middle SDI	11546734 (10130462-13148278)	1056.79 (920.56-1201.66)	13060791 (11423423-14774640)	1087.06 (951.7-1233.34)	0.15 (0.13-0.17)	146640058 (125805581-168593873)	13250.6 (11372.44-15230.64)	191802656 (167163077-220197711)	13502.73 (11610.06-15484.75)	0.1 (0.08-0.12)	5657198 (1090025-12056696)	511.68 (99.74-1085.79)	7409676 (1474227-15665837)	517.57 (92.95-1103.35)	0.08 (0.07-0.1)
High SDI	10193796 (8908219-11542805)	1200.52 (1044.7-1359.69)	11294916 (9902279-12786995)	1222.54 (1070.57-1388.63)	0.08 (0.05-0.11)	142911565 (124064905-163809089)	15286.91 (13196.35-17504.23)	175424404 (153279199-201895737)	15365.14 (13250.29-17765.44)	0.02 (-0.01-0.06)	5376701 (896806-11550291)	573.39 (91.37-1243.24)	6613112 (1212857-14047722)	573.62 (89.78-1236.04)	0.02 (-0.01-0.05)
Low-middle SDI	15222631 (13175203-17497048)	1220.61 (1066.8-1377.82)	24656880 (21420024-27993902)	1213.32 (1057.78-1372.53)	-0.03 (-0.04--0.02)	157234784 (133322760-182843388)	14823.76 (12712.93-17056.16)	288676927 (246439633-332108195)	14786.99 (12677.14-16979.01)	-0.02 (-0.03--0.01)	5750275 (691692-12804149)	543.89 (73.75-1198.25)	10617257 (1368933-23440100)	544.25 (73.88-1201.85)	0 (-0.01-0.01)
Low SDI	5490327 (4696368-6408793)	1047.87 (909.02-1192.81)	12662944 (10811145-14650313)	1045.99 (905.16-1193.48)	0 (-0.01-0)	54342303 (45978744-63691837)	12809.07 (10955.37-14788.52)	129654870 (109563874-152432456)	12808.97 (10909.26-14754.4)	0 (-0.01-0.01)	1989611 (284626-4460947)	472.39 (76.19-1018.8)	4782246 (672593-10758550)	475.21 (76.39-1031.51)	0.03 (0.02-0.04)
Middle SDI	20983717 (18289663-23872475)	1129.43 (987.42-1276.02)	28437798 (25055735-32051776)	1173.23 (1027.7-1322.77)	0.16 (0.14-0.18)	230715535 (195772574-267365243)	13590.78 (11682.69-15580.87)	371941648 (319831620-426895954)	14344.23 (12233.37-16477.29)	0.19 (0.17-0.21)	8611314 (1117748-19168776)	508.49 (72.07-1119.5)	13921317 (2023383-30544094)	535.85 (74.81-1178.27)	0.19 (0.17-0.21)

Supplementary Table S2 Global burden of incidence, prevalence, and DALYs of migraine trends from 1990 to 2021 by age groups.

	Incidence					Prevalence					DALYs				
	1990		2021			1990		2021			1990		2021		
	Cases	ASIR	Cases	ASIR	EAPC	Cases	ASPR	Cases	ASPR	EAPC	Cases	ASDR	Cases	ASDR	EAPC
Characteristics	(95%UI)	pre-100,000 (95%UI)	(95%UI)	pre-100,000 (95%UI)	(95%CI)	(95%UI)	pre-100,000 (95%UI)	(95%UI)	pre-100,000 (95%UI)	(95%CI)	(95%UI)	pre 100,000 (95%UI)	(95%UI)	pre-100,000 (95%UI)	(95%CI)
Age group 1															
<20 years	30159793 (23657105-38243585)	1335.35 (1047.44-1693.26)	36794858 (28682815-46611156)	1395.94 (1088.18-1768.36)	0.08 (0.05-0.11)	165687028 (128625015-212331225)	7335.91 (5694.96-9401.12)	205729235 (158825540-265306014)	7805.07 (6025.61-10065.33)	0.17 (0.1-0.25)	6042776 (397075-15013093)	267.55 (17.58-664.72)	7515775 (486575-18715548)	285.14 (18.46-710.04)	0.18 (0.11-0.26)
20-54 years	30771194 (25277274-37478182)	1280.2 (1051.63-1559.24)	47602532 (39244650-58155627)	1262.83 (1041.1-1542.78)	-0.05 (-0.06--0.03)	483135608 (411973854-567625464)	20100.31 (17139.7-23615.41)	768241848 (659941485-902630065)	20380.33 (17507.28-23945.45)	0.04 (0.03-0.05)	18098499 (2670991-39473830)	752.97 (111.12-1642.26)	28782139 (4270131-62548240)	763.55 (113.28-1659.31)	0.05 (0.04-0.06)
55+ years	2565604 (1978528-3253164)	382.11 (294.68-484.52)	5785997 (4508514-7314923)	389.37 (303.4-492.26)	0.12 (0.08-0.16)	83741827 (68347038-102858446)	12472.24 (10179.39-15319.41)	184461740 (150090773-228051421)	12413.42 (10100.42-15346.81)	0.03 (-0.01-0.06)	3270922 (878905-6888204)	487.16 (130.9-1025.91)	7080975 (1771745-15090148)	476.52 (119.23-1015.5)	-0.03 (-0.06-0)
Age group 2															
<5 years	0 (0-0)	0 (0-0)	0 (0-0)	0 (0-0)	0 (0-0)	0 (0-0)	0 (0-0)	0 (0-0)	0 (0-0)	0 (0-0)	0 (0-0)	0 (0-0)	0 (0-0)	0 (0-0)	0 (0-0)
5-9 years	9375144 (6194247-13204226)	1606.62 (1061.51-2262.82)	11080972 (7322452-15592670)	1612.83 (1065.78-2269.5)	0 (0-0)	14767041 (9823784-20585697)	2530.64 (1683.51-3527.78)	17455196 (11640912-24478807)	2540.59 (1694.32-3562.87)	0 (0-0)	492448 (10460-1374371)	84.39 (1.79-235.53)	584845 (13181-1607283)	85.12 (1.92-233.94)	0 (0-0)
10-14 years	12579737 (9292348-16268003)	2348.36 (1734.67-3036.87)	15792114 (11721390-20366948)	2368.93 (1758.29-3055.18)	0 (0-0)	63492598 (47413937-84121631)	11852.65 (8851.12-15703.63)	79974615 (59777743-106050718)	11996.74 (8967.07-15908.34)	0 (0-0)	2297212 (100959-5782523)	428.84 (18.85-1079.47)	2897557 (125711-7274036)	434.65 (18.86-1091.16)	0 (0-0)
15-19 years	8204911 (4901600-12300017)	1579.62 (943.66-2368.01)	9921772 (5850625-14916214)	1590.08 (937.63-2390.5)	0 (0-0)	87427389 (65664063-111053195)	16831.62 (12641.72-21380.09)	108299425 (81527056-138151358)	17356.24 (13065.65-22140.35)	0 (0-0)	3253115 (283587-7894741)	626.29 (54.6-1519.9)	4033373 (345474-9888374)	646.39 (55.37-1584.73)	0 (0-0)
20-24 years	7087206 (4327134-10508951)	1440.23 (879.34-2135.58)	8706303 (5305143-12962997)	1457.96 (888.4-2170.78)	0 (0-0)	92702395 (71784723-116204849)	18838.58 (14587.78-23614.65)	115809740 (89103285-145179520)	19393.48 (14921.22-24311.74)	0 (0-0)	3433910 (345339-8175474)	697.82 (70.18-1661.38)	4289114 (432044-10181153)	718.25 (72.35-1704.93)	0 (0-0)
25-29 years	6458397 (3965321-9381093)	1459.13 (895.88-2119.45)	8766749 (5391602-12720268)	1490.07 (916.4-2162.05)	0 (0-0)	89517695 (71159768-111182826)	20224.52 (16076.96-25119.27)	120861267 (95650515-150629445)	20542.65 (16257.6-25602.31)	0 (0-0)	3266501 (319415-7626788)	737.99 (72.16-1723.1)	4408485 (417385-10254465)	749.3 (70.94-1742.94)	0 (0-0)
30-34 years	5300556 (3301029-7898584)	1375.26 (856.47-2049.33)	8410353 (5282407-12640359)	1391.34 (873.88-2091.11)	0 (0-0)	81321482 (64338433-102185149)	21099.31 (16692.96-26512.5)	128086794 (101265585-161691640)	21189.58 (16752.51-26748.87)	0 (0-0)	2996220 (359715-6712564)	777.39 (93.33-1741.61)	4714572 (567722-10643665)	779.94 (93.92-1760.8)	0 (0-0)

35-39 years	4888494 (3137984-7491117)	1387.81 (890.85-2126.68)	7894653 (5002754-12201303)	1407.58 (891.97-2175.44)	0 (0-0)	74634932 (60625524-92096761)	21188.39 (17211.21-26145.69)	120786757 (97701173-149277444)	21535.76 (17419.7-26615.52)	0 (0-0)	2805888 (446636-6073871)	796.57 (126.8-1724.33)	4527647 (701860-9891421)	807.26 (125.14-1763.6)	0 (0-0)
40-44 years	3345610 (2141152-4816931)	1167.83 (747.4-1681.41)	5956902 (3787423-8693099)	1190.78 (757.1-1737.75)	0 (0-0)	60993744 (49415167-76120239)	21290.63 (17248.98-26570.72)	107953781 (87558300-135549148)	21579.93 (17502.88-27096.24)	0 (0-0)	2331743 (417838-5165838)	813.92 (145.85-1803.2)	4106009 (702527-9226434)	820.79 (140.44-1844.36)	0 (0-0)
45-49 years	2027689 (1225149-2965040)	873.27 (527.64-1276.96)	4251089 (2591439-6168624)	897.79 (547.29-1302.76)	0 (0-0)	45640333 (36872623-56966355)	19655.99 (15879.99-24533.79)	93847930 (74995724-117496655)	19819.85 (15838.44-24814.26)	0 (0-0)	1766226 (333935-3752713)	760.66 (143.82-1616.19)	3613338 (665254-7760721)	763.11 (140.5-1639)	0 (0-0)
50-54 years	1663241 (997237-2521983)	782.44 (469.13-1186.42)	3616483 (2140856-5410063)	812.83 (481.17-1215.95)	0 (0-0)	38325026 (31354633-48015761)	18029.24 (14750.16-22588.06)	80895579 (66115228-102422980)	18181.93 (14859.93-23020.38)	0 (0-0)	1498010 (318812-3167560)	704.71 (149.98-1490.12)	3122974 (614242-6666750)	701.91 (138.06-1498.41)	0 (0-0)
55-59 years	1075897 (649620-1638424)	580.94 (350.77-884.68)	2393612 (1445944-3592747)	604.86 (365.39-907.88)	0 (0-0)	29740350 (24132044-37540787)	16058.51 (13030.26-20270.41)	64703877 (51991280-82222467)	16350.59 (13138.14-20777.52)	0 (0-0)	1160739 (272014-2475445)	626.75 (146.88-1336.63)	2491134 (534565-5415757)	629.51 (135.08-1368.56)	0 (0-0)
60-64 years	614505 (364205-931519)	382.61 (226.76-579.99)	1270128 (760336-1932642)	396.86 (237.57-603.86)	0 (0-0)	22546233 (18149408-28370132)	14037.94 (11300.35-17664.07)	45753173 (36759042-57868829)	14295.73 (11485.48-18081.31)	0 (0-0)	899526 (248412-1863255)	560.07 (154.67-1160.12)	1792018 (457897-3719823)	559.92 (143.07-1162.27)	0 (0-0)
65-69 years	354312 (205085-508238)	286.64 (165.91-411.16)	838970 (488807-1197912)	304.15 (177.21-434.27)	0 (0-0)	14495292 (11511079-18271466)	11726.69 (9312.46-14781.61)	32724361 (25885728-41521359)	11863.44 (9384.25-15052.58)	0 (0-0)	575548 (163585-1215780)	465.62 (132.34-983.57)	1278141 (338167-2677153)	463.36 (122.59-970.54)	0 (0-0)
70-74 years	228411 (142323-342748)	269.79 (168.11-404.85)	574505 (355448-857122)	279.1 (172.68-416.4)	0 (0-0)	8117863 (6416175-10433902)	9588.65 (7578.65-12324.31)	19987936 (15713240-25850253)	9710.46 (7633.74-12558.47)	0 (0-0)	309481 (86906-661441)	365.55 (102.65-781.28)	752495 (202119-1627116)	365.57 (98.19-790.48)	0 (0-0)
75-79 years	164622 (106154-246398)	267.44 (172.45-400.29)	362926 (231015-539302)	275.18 (175.16-408.92)	0 (0-0)	5025137 (3953200-6459583)	8163.59 (6422.17-10493.91)	10794541 (8429390-13881771)	8184.85 (6391.5-10525.71)	0 (0-0)	187132 (49266-395236)	304.01 (80.03-642.08)	393916 (95783-848808)	298.68 (72.63-643.6)	0 (0-0)
80-84 years	92776 (60627-138225)	262.26 (171.38-390.73)	233764 (154960-345650)	266.91 (176.93-394.65)	0 (0-0)	2569487 (2028308-3359548)	7263.38 (5733.58-9496.71)	6385715 (5036567-8369713)	7291.04 (5750.62-9556.32)	0 (0-0)	93868 (27337-200319)	265.34 (77.28-566.26)	229649 (63419-495371)	262.21 (72.41-565.6)	0 (0-0)
85-89 years	29988 (18700-44522)	198.45 (123.75-294.63)	90910 (56899-133440)	198.83 (124.45-291.85)	0 (0-0)	978880 (760019-1249751)	6477.9 (5029.55-8270.43)	2954857 (2277253-3814786)	6462.69 (4980.67-8343.47)	0 (0-0)	35535 (11701-71870)	235.16 (77.43-475.61)	105140 (32895-217228)	229.96 (71.95-475.11)	0 (0-0)
90-94 years	4806 (3256-6733)	112.15 (75.99-157.12)	19687 (13228-27656)	110.05 (73.95-154.6)	0 (0-0)	227901 (176616-292865)	5318.33 (4121.54-6834.37)	945477 (733660-1226626)	5285.13 (4101.09-6856.73)	0 (0-0)	7844 (2087-16252)	183.05 (48.69-379.27)	32051 (8082-67494)	179.16 (45.18-377.29)	0 (0-0)

95+ years	287 (195-401)	28.17 (19.11-39.39)	1495 (1008-2102)	27.44 (18.5-38.56)	0 (0-0)	40684 (30042-55702)	3996.14 (2950.85-5471.28)	211803 (153698-292506)	3886.07 (2819.99-5366.77)	0 (0-0)	1249 (204-2859)	122.66 (20.01-280.8)	6433 (1005-14852)	118.03 (18.43-272.49)	0 (0-0)

Supplementary Table S3 Global burden of incidence, prevalence, and DALYs of migraine trends from 1990 to 2021 by 21 GBD regions.

	Incidence					Prevalence					DALYs				
	1990		2021			1990		2021			2019		2021		
	Cases	ASIR	Cases	ASIR	EAPC	Cases	ASPR	Cases	ASPR	EAPC	Cases	ASDR	Cases	ASDR	EAPC
Characteristics	(95%UI)	pre-100,000 (95%UI)	(95%UI)	pre-100,000 (95%UI)	(95%CI)	(95%UI)	pre-100,000 (95%UI)	(95%UI)	pre-100,000 (95%UI)	(95%CI)	(95%UI)	pre-100,000 (95%UI)	(95%UI)	pre-100,000 (95%UI)	(95%CI)
Central Asia	789499 (666279-929077)	1103.56 (937.96-1284.07)	1061285 (899936-1224759)	1098.44 (933.29-1278.26)	-0.02 (-0.02--0.02)	8840056 (7473807-10470453)	13652.82 (11597.77-15992.77)	13240030 (11217466-15557584)	13584.23 (11538.76-15918.11)	-0.02 (-0.02--0.02)	328067 (54274-757795)	509.92 (90.41-1154.76)	494679 (87858-1116618)	507.35 (90.34-1144.53)	-0.01 (-0.01-0)
East Asia	11956379 (10465337-13649232)	921.02 (810.93-1041.57)	13524743 (12000700-15250401)	976.85 (863.27-1104)	0.23 (0.2-0.27)	138666855 (118605236-159459079)	10993.18 (9469.04-12636.81)	191704024 (166846938-221713279)	11798.36 (10162.49-13566.96)	0.28 (0.23-0.32)	5223003 (792491-11671556)	414.51 (65.94-912.61)	7248392 (1171603-15744802)	444.29 (66.76-971.75)	0.26 (0.22-0.31)
South Asia	14390076 (12570152-16431074)	1228.87 (1083.39-1384.22)	24072217 (21074567-27098809)	1231.08 (1079.82-1384.65)	-0.01 (-0.03-0.01)	149366878 (126775559-173676130)	14802.76 (12738.78-16920.44)	286372320 (243859319-328165148)	14859.95 (12704.02-16953.54)	-0.01 (-0.04-0.01)	5376355 (613082-12091289)	534.33 (68.74-1176.97)	10362369 (1252047-23272971)	537.87 (68.02-1198.33)	0 (-0.02-0.03)
High-income Asia Pacific	1523263 (1330122-1739801)	883.2 (768.76-1003.72)	1286379 (1129143-1464113)	889.82 (774.85-1020.15)	0.02 (0.01-0.04)	20690013 (17886452-23688798)	10994.8 (9430.47-12641.95)	21172927 (18371952-24296846)	11072.69 (9484.03-12800.27)	0.01 (0-0.03)	795621 (173438-1733758)	419.82 (87.76-926.2)	827640 (204646-1773190)	422.48 (85.63-937.71)	0.01 (0-0.02)
Southeast Asia	6726454 (5799933-7753562)	1324.8 (1154.03-1506.82)	9325683 (8147718-10607424)	1313.08 (1144.83-1493.55)	-0.03 (-0.03--0.02)	73238315 (62188747-85859177)	16511.86 (14258.73-19090.42)	119860813 (103266386-139236440)	16180.66 (13890.15-18730.39)	-0.07 (-0.08--0.06)	2735426 (274369-6180371)	617.23 (69.42-1393.25)	4504043 (522076-10047421)	607.13 (69.7-1362.67)	-0.05 (-0.06--0.04)
Western Europe	4903597 (4262651-5589664)	1395.9 (1203.53-1592.51)	4913868 (4280234-5568359)	1399.05 (1207.74-1596.87)	0.03 (0.01-0.04)	73109318 (63425674-84648487)	18024.44 (15507.01-20879.71)	80359608 (70005542-93109621)	18170.7 (15724.92-21091.55)	0.05 (0.02-0.07)	2741839 (436308-5957728)	672.65 (97.78-1467.33)	3026513 (527630-6473769)	676.64 (96.68-1471.91)	0.05 (0.02-0.08)
Central Europe	1367968 (1183676-1574578)	1104.64 (950.96-1264.75)	1109874 (965027-1271787)	1101.88 (949.54-1260.69)	-0.01 (-0.01--0.01)	18291301 (15793104-20986126)	13875.12 (11890.58-15942.23)	17302364 (15177932-19780848)	13822.04 (11854.14-15865.31)	-0.02 (-0.02--0.01)	696245 (150324-1518640)	525.7 (108.88-1162.65)	666285 (159796-1436227)	525.24 (109.75-1158.95)	0 (0-0.01)
Eastern Europe	2360125 (2076600-2662978)	1072.98 (943.59-1209.82)	2009050 (1775940-2265372)	1072.44 (944.27-1208.65)	0 (0-0.01)	33928597 (29522013-38764398)	14104.71 (12243.32-16148.4)	32050566 (27899660-36442161)	14077.09 (12210.58-16112.04)	0.01 (-0.01-0.03)	1397301 (420534-2853438)	575.35 (163.47-1195.27)	1336530 (424523-2695927)	574.43 (164.13-1193.9)	0.04 (0.01-0.08)
Central Sub-Saharan Africa	579115 (481962-681980)	1015.16 (859.54-1178.4)	1504410 (1249765-1767533)	1012.86 (857.59-1175.82)	-0.01 (-0.01--0.01)	5733652 (4770578-6806302)	12430.14 (10518.04-14549.86)	15114474 (12560504-17911094)	12396.15 (10491.24-14511.21)	-0.01 (-0.01--0.01)	209345 (31319-480213)	458.37 (80.73-1002.44)	557713 (84070-1267911)	461.37 (80.99-1008.48)	0.03 (0.03-0.04)
Eastern Sub-Saharan Africa	1454196 (1233422-1708145)	743.73 (639.85-859.59)	3430178 (2910103-4018337)	750.03 (643.13-866.75)	0.05 (0.04-0.05)	13945177 (11701420-16332229)	8991.43 (7699.25-10349.49)	34295581 (28749230-40314574)	9084.26 (7732.97-10507.26)	0.05 (0.04-0.06)	523409 (104560-1192625)	343.01 (78.94-745.23)	1292756 (253046-2954613)	347.19 (77.86-757.55)	0.07 (0.06-0.08)
North Africa and Middle East	4557395 (3834263-5318522)	1225.48 (1049.74-1409.2)	8062426 (6902519-9276914)	1233.46 (1059.84-1414.98)	0.02 (0.01-0.03)	47293467 (39752846-55981426)	15229.55 (12887.62-17791.59)	98207712 (83689909-114281976)	15304.13 (13071.93-17749.48)	0.01 (0-0.02)	1847327 (304873-4143240)	597.9 (110-1308.5)	3843165 (678200-8368174)	598.94 (108.94-1305.73)	0.01 (0-0.02)
Southern Sub-Saharan Africa	588247 (509798-	1046.86 (922.03-	884850 (774238-	1042.48 (917.99-	-0.02 (-0.02--	6168980 (5215971-	12907.41 (11120.67-	10526174 (9015593-	12876.73 (11102.68-	-0.01 (-0.02--	229187 (36658-	483.87 (87.08-	389159 (65893-	477.47 (85.49-	-0.04 (-0.04--

	676656)	1186.74)	1005870)	1182.35)	0.01)	7187176)	14884.46)	12175770)	14843.51)	0.01)	508858)	1037.35)	848009)	1025.39)	0.04)
Western Sub-Saharan Africa	2436165 (2079512-2812051)	1196.41 (1037.34-1363.54)	6386770 (5468054-7431561)	1196.41 (1037.48-1367.14)	0 (-0.01-0)	24421215 (20649703-28941317)	14798.36 (12615.24-17173.37)	64362037 (54002945-76098769)	14870.35 (12715.58-17284.92)	0.02 (0.01-0.02)	896465 (118975-2017423)	547.94 (83.59-1196.86)	2380161 (312311-5415290)	554.49 (84.75-1214.04)	0.05 (0.04-0.05)
Caribbean	437592 (369830-506992)	1178.52 (1004.97-1351.91)	538364 (459614-618106)	1173.66 (1000.85-1346.88)	-0.02 (-0.02--0.01)	5033062 (4213385-5904401)	14280.42 (12042.61-16582.87)	6949002 (5879712-8046252)	14237.12 (12004.33-16529.9)	-0.01 (-0.01--0.01)	186857 (23244-410329)	532.28 (71.93-1156.04)	258591 (36828-563192)	528.99 (72.3-1148.15)	-0.02 (-0.02--0.01)
High-income North America	3634544 (3202287-4078746)	1337.58 (1173.51-1504.31)	4243346 (3781552-4818661)	1315.37 (1163.87-1495.98)	0 (-0.08-0.07)	50690766 (44103609-57797617)	17052.08 (14768.04-19443.91)	63986396 (55643985-73406886)	16709.11 (14462.52-19276.29)	-0.01 (-0.1-0.07)	1886839 (272278-4126944)	633.85 (89.5-1390.8)	2366730 (390229-5145721)	614 (89.37-1352.42)	-0.04 (-0.12-0.04)
Andean Latin America	358337 (303134-417637)	856.38 (734.82-989.6)	600721 (510030-698309)	892.05 (755.93-1037.2)	0.15 (0.11-0.19)	3619412 (3096182-4199549)	9979.03 (8656.54-11428.59)	7215331 (6090862-8396291)	10614.71 (8993.46-12309.15)	0.23 (0.17-0.28)	135991 (22428-311607)	380.13 (72.55-845.92)	272814 (48480-611280)	401.89 (72.75-893.96)	0.21 (0.16-0.27)
Central Latin America	2095876 (1794261-2425559)	1149.09 (996.99-1306.02)	2950118 (2559379-3343268)	1157.99 (1005.9-1315.14)	0.03 (0.03-0.04)	21922078 (18583566-25762686)	13978.54 (12095.52-16164.56)	37489338 (32024849-43508286)	14178.67 (12097.7-16441.61)	0.06 (0.05-0.07)	818750 (102461-1811604)	525.25 (76.27-1142.18)	1406733 (203912-3062757)	531.67 (76.53-1159.87)	0.05 (0.05-0.06)
Southern Latin America	474171 (398721-551230)	940.57 (792.77-1094.74)	632668 (538296-735076)	956.77 (809.71-1115.09)	0.09 (0.07-0.1)	5590142 (4735658-6482211)	11393.96 (9682.23-13161.22)	8402383 (7195356-9789775)	11706.98 (9960.37-13667.23)	0.13 (0.11-0.15)	211967 (41409-466281)	432.96 (85.95-950.23)	318959 (64871-694704)	442.43 (85.81-974.19)	0.11 (0.09-0.13)
Tropical Latin America	2561528 (2225950-2929068)	1476.13 (1293.29-1666.31)	3173630 (2813108-3563783)	1508.33 (1325.98-1721.96)	0.14 (0.08-0.21)	28304439 (24432746-32558721)	18169.84 (15789.78-20875.59)	43632768 (37739023-50335352)	18595.34 (16084.24-21444.51)	0.13 (0.08-0.17)	1034784 (96974-2348850)	664.84 (72.78-1496.14)	1594752 (187100-3590843)	679.39 (73.91-1542.87)	0.15 (0.11-0.18)
Oceania	81710 (68708-96049)	1165.61 (996.34-1349.62)	170809 (144885-198569)	1166.8 (997.06-1350.79)	0 (0-0)	833531 (697006-995581)	14023.1 (11914.15-16402.7)	1864400 (1571972-2212470)	14043.19 (11934.61-16427.13)	0 (0-0)	30685 (3354-70498)	517.39 (64.64-1149.48)	68892 (7962-154920)	519.02 (64.65-1151.04)	0.01 (0.01-0.01)
Australasia	220354 (189852-254734)	1094.95 (943.28-1263.76)	301998 (260776-349469)	1094.94 (943.48-1263.28)	0 (0-0)	2877210 (2461783-3324698)	13422.27 (11422.92-15611.53)	4324577 (3732120-4964956)	13433.71 (11438.78-15621.49)	0 (0-0.01)	106734 (18905-234856)	496.96 (86.59-1095.2)	162013 (31164-351254)	498.64 (86.96-1100.43)	0.01 (0.01-0.01)
Central Asia	789499 (666279-929077)	1103.56 (937.96-1284.07)	1061285 (899936-1224759)	1098.44 (933.29-1278.26)	-0.02 (-0.02--0.02)	8840056 (7473807-10470453)	13652.82 (11597.77-15992.77)	13240030 (11217466-15557584)	13584.23 (11538.76-15918.11)	-0.02 (-0.02--0.02)	328067 (54274-757795)	509.92 (90.41-1154.76)	494679 (87858-1116618)	507.35 (90.34-1144.53)	-0.01 (-0.01-0)

Supplementary Table S4 Global burden of incidence, prevalence, and DALYs of migraine trends from 1990 to 2021 by countries and regions.

	Incidence					Prevalence					DALYs				
	1990		2021			1990		2021			2019		2021		
	Cases	ASIR	Cases	ASIR	EAPC	Cases	ASPR	Cases	ASPR	EAPC	Cases	ASDR	Cases	ASDR	EAPC
Characteristics	(95%UI)	pre-100,000 (95%UI)	(95%UI)	pre-100,000 (95%UI)	(95%CI)	(95%UI)	pre-100,000 (95%UI)	(95%UI)	pre-100,000 (95%UI)	(95%CI)	(95%UI)	pre-100,000 (95%UI)	(95%UI)	pre-100,000 (95%UI)	(95%CI)
Afghanistan	126541 (105219-149777)	1259.36 (1074.4-1453.99)	423386 (352317-502283)	1219.25 (1037.68-1408.34)	-0.1 (-0.11--0.09)	1323904 (1105654-1572234)	15624.3 (13231.58-18367.97)	4189017 (3477676-5015574)	15219.55 (12883.74-17868.2)	-0.09 (-0.1--0.09)	50870 (8417-115602)	600.21 (108.16-1316.74)	161054 (24435-362078)	585.63 (106.64-1275.39)	-0.07 (-0.07--0.06)
Albania	37926 (32110-43926)	1084.87 (921.45-1256.37)	26299 (22549-30544)	1084.7 (921.54-1253.1)	-0.01 (-0.02-0.01)	432667 (363836-510931)	13506.04 (11419.58-15637.9)	392413 (337141-452887)	13598.05 (11517.73-15739.28)	0.02 (0-0.03)	16311 (3072-37468)	513.49 (105.1-1142.87)	15072 (3582-32682)	516.38 (111.31-1154.53)	0.03 (0.02-0.05)

Algeria	344960 (287711-408786)	1221.87 (1039.79-1411.47)	552445 (471211-641705)	1221.98 (1040.13-1411.94)	0 (0-0)	3483367 (2894156-4153317)	15189.94 (12861.67-17836.76)	6774420 (5721042-7992299)	15170.66 (12846.21-17816.36)	0 (0-0)	136109 (21552-310432)	596.68 (108.8-1312.04)	265858 (47493-576873)	595.45 (107.32-1299.93)	0 (-0.01-0)
American Samoa	597 (505-698)	1166.54 (997.5-1350.52)	606 (512-704)	1168.44 (998.78-1353.23)	0.01 (0.01-0.01)	6273 (5258-7496)	14037.07 (11923.7-16420.89)	7144 (6083-8331)	14058.93 (11945.16-16446.43)	0.01 (0.01-0.02)	233 (26-534)	521.64 (64.9-1160.66)	264 (33-589)	518.16 (64.93-1156.39)	-0.01 (-0.02-0)
Andorra	736 (625-869)	1353.41 (1148.07-1560.21)	955 (814-1109)	1364.67 (1158.41-1572.01)	0.02 (0.01-0.03)	10629 (9073-12567)	17192.05 (14618.8-20245.45)	16183 (13862-18949)	17391.72 (14783.07-20499.22)	0.04 (0.03-0.05)	397 (57-870)	641.05 (91.5-1407.23)	611 (105-1324)	646.76 (91.47-1428.97)	0.04 (0.03-0.05)
Angola	108300 (90065-127309)	1010.54 (855.38-1173.45)	358047 (298034-422486)	1020.29 (864.05-1184.37)	0.03 (0.03-0.03)	1074380 (892773-1274696)	12331.64 (10435.32-14438.77)	3462969 (2878204-4111642)	12500.79 (10578.55-14633.25)	0.05 (0.04-0.05)	39598 (5936-90103)	459.34 (81.39-996.46)	127821 (18995-293839)	465.75 (81.18-1019.39)	0.06 (0.06-0.07)
Antigua and Barbuda	746 (632-863)	1182.35 (1008.46-1355.75)	985 (841-1128)	1176.32 (1003.22-1349.47)	-0.03 (-0.04--0.02)	8703 (7288-10179)	14364.77 (12114.85-16677.48)	13745 (11658-15936)	14278.95 (12038.71-16576.64)	-0.03 (-0.05--0.02)	324 (42-711)	537.41 (73.56-1175.37)	514 (77-1124)	532.29 (73.17-1162.64)	-0.04 (-0.05--0.02)
Argentina	312121 (262187-363873)	934.21 (784.91-1088.82)	430368 (365787-502252)	955.45 (809.5-1115.13)	0.11 (0.09-0.13)	3656451 (3094491-4231484)	11254.89 (9539.46-13051.15)	5586220 (4760124-6541042)	11636.39 (9880.11-13555.46)	0.15 (0.13-0.18)	139157 (27734-305278)	428.69 (85.92-940.04)	212471 (42666-467065)	441.01 (85.37-971.63)	0.13 (0.11-0.16)
Armenia	38566 (32706-45027)	1103.65 (938.54-1283.7)	30861 (26299-35815)	1097.98 (933.65-1277.04)	-0.02 (-0.03--0.02)	464665 (393098-549415)	13655.67 (11600.92-15997.17)	437577 (374798-511583)	13628.9 (11574.73-15971.61)	-0.01 (-0.02-0)	17337 (2974-39495)	510.89 (90.33-1156.21)	16485 (3166-36641)	510.08 (89.82-1158.33)	0.01 (0-0.02)
Australia	181891 (155325-211381)	1087.9 (928.53-1260.57)	249307 (213859-288834)	1088.74 (929.52-1261.42)	0 (0-0)	2377221 (2023733-2762820)	13314.52 (11265.19-15542.09)	3571618 (3065934-4120714)	13338.06 (11292.05-15572.72)	0 (0-0.01)	88248 (15628-194719)	493.21 (85.42-1088.46)	133811 (25591-291010)	494.98 (85.55-1091.17)	0.01 (0-0.01)
Austria	93302 (80558-107998)	1318.31 (1128.98-1520.18)	95368 (81877-109645)	1314.38 (1125.54-1514.2)	0.03 (0.01-0.06)	1369266 (1178905-1587668)	16561.81 (14139.3-19295.19)	1522683 (1315123-1761092)	16466.12 (14043.9-19214.85)	0.06 (0.01-0.12)	51507 (8850-111178)	619.64 (98.01-1361.5)	57720 (10769-124520)	615.73 (95.3-1358.32)	0.06 (0.01-0.11)
Azerbaijan	84341 (71507-98602)	1105.32 (940.45-1285.31)	116123 (98935-134573)	1092.43 (928.54-1270.91)	-0.05 (-0.05--0.04)	971509 (822253-1152652)	13692.3 (11634.56-16038.34)	1551485 (1317970-1820180)	13532.87 (11495.4-15857.29)	-0.04 (-0.05--0.04)	36194 (6069-82918)	513.41 (93.2-1158.02)	58295 (10896-130062)	506.66 (91.7-1145.83)	-0.03 (-0.04--0.02)
Bahamas	3336 (2820-3868)	1178.46 (1005.06-1351.64)	4568 (3891-5240)	1181.01 (1007.15-1354.57)	0.01 (0.01-0.01)	38019 (31689-44774)	14326.26 (12081.39-16635)	60687 (51343-70212)	14343.18 (12095.49-16655.34)	0 (0-0.01)	1415 (172-3104)	536.09 (73.28-1165.96)	2264 (322-4955)	533.91 (73.23-1163.89)	0 (-0.01-0)
Bahrain	6566 (5554-7736)	1173.24 (995.73-1354.8)	17935 (15163-21099)	1150.76 (975.65-1332.19)	-0.1 (-0.12--0.09)	75077 (62744-89825)	14352.45 (12118.38-16865.73)	244147 (205558-287764)	14027.17 (11846.08-16450.67)	-0.11 (-0.13--0.09)	2949 (481-6485)	565.49 (102.58-1232.28)	9642 (1787-20423)	551.51 (100.93-1208.25)	-0.1 (-0.12--0.09)
Bangladesh	1419252 (1186790-1668500)	1208.43 (1029.1-1385.25)	2106738 (1788769-2421878)	1219.24 (1039.6-1397.43)	0.04 (0.04-0.05)	13735312 (11473049-16531211)	14431.95 (12188.29-16876.45)	24942554 (21005793-29258181)	14622.91 (12354.85-17096.06)	0.05 (0.05-0.06)	494384 (50923-1130534)	521.53 (64.26-1164.16)	903347 (108412-2037192)	529.83 (65.93-1188.38)	0.07 (0.06-0.08)
Barbados	2987 (2540-3430)	1178.52 (1005.32-1351.86)	3005 (2583-3437)	1175.27 (1002.5-1348.09)	-0.01 (-0.01--0.01)	37573 (31687-43569)	14347.96 (12099.25-16651.76)	44624 (37935-51620)	14292.5 (12051-16591.2)	-0.01 (-0.01--0.01)	1406 (188-3079)	537.63 (73.22-1181.27)	1677 (266-3646)	533.58 (71.47-1161.07)	-0.01 (-0.02--0.01)
Belarus	102571 (87418-119150)	1014.63 (859.51-1169.4)	84229 (71471-97497)	1009.66 (855.42-1163.87)	-0.01 (-0.02--0.01)	1439861 (1244332-1668071)	13092.57 (11253.84-15178.18)	1331465 (1154466-1524260)	13015.65 (11182.02-15082.62)	-0.01 (-0.02--0.01)	58944 (16935-122535)	530.86 (143.75-1127.25)	55176 (17072-112397)	526.93 (143.58-1111.25)	0 (-0.01-0.01)
Belgium	130764 (111805-150773)	1472.84 (1270.43-1694.54)	139419 (119852-158091)	1506.95 (1288.44-1714.3)	0.08 (0.06-0.1)	2125321 (1806845-2475835)	20204.62 (17185.49-23488.71)	2544608 (2192802-2985665)	21751.47 (18730.05-25705.5)	0.26 (0.19-0.33)	79080 (11026-173778)	749.28 (94.12-1655.37)	94015 (12767-205440)	800.36 (91.99-1772.02)	0.24 (0.17-0.3)
Belize	2480 (2039-2953)	1168.92 (996.36-1342.48)	5429 (4592-6256)	1177.15 (1003.73-1350.57)	0.03 (0.03-0.03)	24170 (19903-28896)	14117.25 (11903.84-16393.51)	64438 (54007-75422)	14249.4 (12014.16-16547.3)	0.03 (0.03-0.03)	897 (99-2024)	529.2 (72.18-1151.43)	2393 (304-5249)	530.3 (72.98-1166.57)	0.02 (0.02-0.02)

Benin	59863 (49757-70188)	1176.67 (1001.52-1354.45)	171600 (143773-201602)	1171.89 (997.19-1349.97)	-0.01 (-0.01--0.01)	570357 (475964-689739)	14655.22 (12435.91-17302.99)	1711350 (1430769-2065089)	14559.28 (12342.52-17194.87)	-0.02 (-0.02--0.02)	20840 (2618-47459)	540.72 (82.43-1193.17)	63209 (8047-144774)	542.17 (83.22-1210.82)	0.01 (0-0.01)
Bermuda	667 (568-772)	1176.69 (1003.61-1350.11)	583 (503-667)	1174.28 (1001.37-1347.36)	-0.01 (-0.01-0)	9126 (7719-10608)	14286.15 (12048.02-16586.93)	9176 (7864-10636)	14239.41 (12009.02-16531.5)	-0.01 (-0.02-0)	343 (48-746)	536.6 (72.1-1166.26)	348 (61-754)	534.4 (73.47-1167.12)	-0.01 (-0.02-0)
Bhutan	8394 (7038-9823)	1201.85 (1023.53-1377.96)	9636 (8166-11181)	1208.09 (1029.06-1385.08)	0.01 (0.01-0.01)	82944 (68852-99669)	14374.3 (12145.85-16792.63)	117267 (98756-138491)	14430.27 (12190.24-16863.35)	0.01 (0-0.01)	3000 (304-6982)	521.78 (64.55-1168.67)	4274 (500-9727)	526.35 (64.36-1188.32)	0.03 (0.03-0.03)
Bolivia (Plurinational State of)	64258 (53280-75733)	918.4 (775.38-1066.72)	112129 (94425-130296)	912.62 (770.19-1059.51)	-0.02 (-0.02--0.02)	643653 (537386-757699)	10974.99 (9309.54-12750.14)	1310700 (1104327-1522133)	10894.77 (9242.26-12658.15)	-0.03 (-0.03--0.02)	23942 (3751-54256)	412.89 (75.4-919.92)	49148 (8220-109554)	409.78 (71.85-901.91)	-0.02 (-0.02--0.01)
Bosnia and Herzegovina	50422 (42807-58452)	1088.18 (924.7-1258.36)	31326 (26868-36257)	1089.27 (925.71-1259.42)	-0.01 (-0.02-0)	653962 (554788-757463)	13644.37 (11553.67-15793.01)	491056 (425225-566029)	13632.57 (11539.12-15778.58)	-0.02 (-0.03--0.01)	24825 (5089-55507)	517.38 (105-1152.54)	18815 (4515-40505)	515.27 (107.38-1145.56)	-0.01 (-0.02-0)
Botswana	14615 (12140-17294)	1023.12 (866.56-1187.79)	26060 (22004-30518)	1013.25 (858.02-1176.2)	-0.03 (-0.03--0.03)	142951 (118799-170248)	12533.86 (10608.43-14673.53)	309365 (259829-364189)	12415.02 (10510.31-14529.56)	-0.03 (-0.03--0.03)	5298 (778-12126)	469.88 (81.48-1024.78)	11452 (1873-25553)	461.18 (81.25-1002.53)	-0.04 (-0.05--0.03)
Brazil	2493987 (2167467-2850790)	1476.78 (1295.1-1666.7)	3066528 (2722906-3445811)	1510.77 (1329.81-1724.03)	0.15 (0.08-0.22)	27622061 (23832289-31763338)	18185.3 (15800.95-20898.84)	42324293 (36640635-48830055)	18631.33 (16120.05-21484.83)	0.13 (0.09-0.18)	1009664 (94934-2291156)	665.28 (72.99-1498.46)	1546789 (182557-3486213)	680.61 (74.08-1546.26)	0.15 (0.11-0.19)
Brunei Darussalam	2455 (2051-2904)	850.77 (717.07-996.05)	4013 (3357-4739)	849.9 (717.22-994.48)	-0.02 (-0.04-0.01)	26801 (22381-31979)	10246.73 (8710.14-12016.22)	52654 (44637-62276)	10292.5 (8762.26-12052.11)	0.01 (-0.01-0.03)	1007 (179-2342)	391.91 (81.87-871.93)	2015 (406-4508)	393.16 (80.04-879.2)	0 (-0.02-0.03)
Bulgaria	89628 (76465-103643)	1092.64 (928.88-1263.33)	62165 (53191-72188)	1086.68 (923.3-1256.69)	-0.02 (-0.02--0.02)	1274410 (1098570-1466371)	13674.51 (11575.02-15825.23)	994945 (861409-1144457)	13595.28 (11506.72-15736.87)	-0.03 (-0.03--0.02)	48749 (10759-105055)	518.05 (104.48-1154.05)	38217 (9301-82360)	514.42 (106.41-1139.61)	-0.01 (-0.02--0.01)
Burkina Faso	118084 (97900-138966)	1178.87 (1003.74-1357.77)	287006 (240314-337516)	1176.22 (1000.93-1354.16)	-0.01 (-0.01--0.01)	1129199 (938917-1359090)	14672.17 (12455.48-17324.84)	2863363 (2391127-3457629)	14610.82 (12392.46-17250.26)	-0.02 (-0.02--0.02)	41360 (5363-94410)	542.24 (81.92-1200.48)	106089 (13365-243014)	546.1 (82.84-1210.87)	0.03 (0.03-0.04)
Burundi	42762 (35839-50610)	759.74 (646.33-890.18)	106099 (88547-125825)	752.93 (641.16-882.57)	-0.04 (-0.04--0.03)	413998 (346751-489471)	9174.81 (7818.51-10698.78)	1035657 (866198-1224076)	9028.71 (7684.63-10533.8)	-0.06 (-0.07--0.06)	15625 (3084-35566)	352.14 (79.8-764.63)	39199 (7847-89649)	347.39 (81.37-756.47)	-0.04 (-0.05--0.03)
Cabo Verde	4383 (3667-5176)	1183.06 (1007.37-1362.88)	6868 (5830-7962)	1162.3 (988.83-1339.15)	-0.06 (-0.06--0.06)	43894 (36500-52991)	14780.35 (12550.64-17456.91)	85966 (72989-101829)	14422.31 (12216.44-17028.65)	-0.08 (-0.09--0.08)	1632 (210-3730)	554.04 (83.65-1228.44)	3212 (466-7149)	539.81 (81.66-1188.79)	-0.08 (-0.08--0.08)
Cambodia	141250 (118545-166560)	1304.71 (1110.97-1505.45)	231235 (195873-268384)	1287.01 (1095.97-1486.11)	-0.04 (-0.05--0.04)	1405962 (1179591-1688114)	16119.94 (13691.95-19020.49)	2753303 (2328219-3245974)	15875.26 (13492.89-18692.53)	-0.05 (-0.05--0.05)	51886 (4804-119957)	596.06 (64.38-1351.63)	102892 (10642-238208)	592.98 (64.56-1360.5)	-0.01 (-0.01-0)
Cameroon	128550 (107444-151069)	1172.06 (997.45-1349.12)	410002 (344346-479769)	1168.14 (993.99-1345.34)	-0.01 (-0.01--0.01)	1276870 (1066955-1541480)	14556.98 (12340.49-17192.49)	4184985 (3502856-5051871)	14498.08 (12285.63-17124.85)	-0.02 (-0.02--0.01)	46875 (6014-107326)	538.63 (82-1194.49)	154722 (19198-354563)	539.8 (82.07-1196.09)	0.02 (0.01-0.02)
Canada	332319 (285815-382865)	1255.77 (1081.27-1443.61)	390646 (336897-447807)	1247.29 (1065.13-1444.02)	-0.01 (-0.02--0.01)	4883455 (4286091-5550485)	16635.52 (14551.27-18910.94)	6323301 (5470147-7383159)	16333.55 (13952.32-19172.28)	-0.06 (-0.08--0.05)	182296 (26211-397710)	619.94 (86.85-1349.97)	237313 (39337-514041)	607.71 (85.91-1322.9)	-0.07 (-0.09--0.05)
Central African Republic	28766 (24001-33789)	1016.74 (861.17-1180.37)	60295 (49967-70637)	1017.46 (861.77-1181.26)	0 (0-0.01)	290420 (241789-344954)	12455.93 (10542.95-14581.95)	618176 (514266-733541)	12462.33 (10548.39-14592.32)	0 (-0.01-0.01)	10589 (1623-23990)	458.23 (80.58-995.18)	22754 (3456-51401)	461.89 (82.16-1002.24)	0.04 (0.03-0.04)
Chad	73147 (60909-86039)	1177.26 (1001.71-1356.36)	223226 (184231-265094)	1171.29 (996.61-1349.66)	-0.02 (-0.02--0.02)	714105 (595067-861960)	14631.33 (12411.8-17280.6)	2076159 (1720186-2510071)	14502.42 (12293.33-17142.09)	-0.03 (-0.03--0.03)	26216 (3357-59980)	541.89 (82.51-1197.55)	76369 (9317-176526)	538.42 (82.21-1196.21)	-0.02 (-0.03--0.02)
Chile	132778	951.27	171944	960.08	0.06	1563658	11656.79	2397438	11862.87	0.1	58718	440.42	90556	445.42	0.09

	(111773-154565)	(804.15-1107.35)	(146130-200362)	(813.83-1115.96)	(0.04-0.08)	(1318112-1832567)	(9934.13-13568.3)	(2048235-2801326)	(10105.06-13878.05)	(0.08-0.12)	(10919-130283)	(86.64-970.19)	(19078-195348)	(87.91-971.45)	(0.06-0.11)
China	11518098 (10091942-13156842)	917.35 (808.35-1036.95)	13047221 (11597731-14698852)	975.61 (862.32-1102.06)	0.24 (0.21-0.28)	133474536 (114199444-153482598)	10948.52 (9428.76-12586.13)	184752280 (160836525-213633958)	11777.51 (10137.56-13538.56)	0.28 (0.24-0.33)	5028787 (767668-11262271)	412.97 (66.16-911.02)	6988199 (1133319-15186289)	443.65 (66.93-971.68)	0.27 (0.23-0.32)
Colombia	414627 (354411-481266)	1164.48 (999.21-1336.58)	553153 (471693-634435)	1166.75 (989.78-1342.9)	0.02 (0.01-0.02)	4547775 (3812767-5417849)	14220.03 (12087.97-16549.38)	7469770 (6249064-8788758)	14382.09 (12019.26-16887.54)	0.05 (0.04-0.06)	169941 (20142-375849)	534.06 (72.38-1169.56)	280616 (39186-614034)	539.64 (72.85-1185.67)	0.06 (0.05-0.07)
Comoros	3625 (3030-4325)	757.08 (644.07-887.7)	5940 (5031-6964)	753.2 (640.74-882.51)	-0.02 (-0.02--0.02)	35071 (29359-41462)	9126.09 (7771.44-10640.8)	66936 (56467-78295)	9086.74 (7742.83-10596.45)	-0.02 (-0.02--0.02)	1332 (262-3080)	351.66 (82.11-774.93)	2573 (557-5738)	351.44 (81.41-766.28)	0.01 (0-0.01)
Congo	26163 (21721-30844)	1017.05 (861.1-1180.6)	59792 (50041-69798)	1013.98 (858.4-1177.41)	-0.01 (-0.01--0.01)	259967 (216108-309204)	12461.88 (10546.3-14585.13)	651923 (545310-773289)	12387.53 (10482.02-14504.96)	-0.02 (-0.02--0.02)	9594 (1449-21744)	464.34 (81.68-1015.89)	24191 (3795-54393)	462.17 (80.37-1007.98)	-0.01 (-0.01-0)
Cook Islands	235 (198-275)	1165.38 (995.92-1349.76)	198 (170-227)	1181.86 (1010.46-1365.98)	0.05 (0.05-0.06)	2551 (2157-3031)	14001.57 (11895.23-16376.32)	2631 (2261-3059)	14255.07 (12115.39-16681.68)	0.06 (0.06-0.07)	95 (11-215)	521.53 (63.52-1165.7)	97 (13-215)	527.28 (65.45-1175.07)	0.04 (0.04-0.05)
Costa Rica	38189 (31914-44924)	1147.19 (972.32-1328.57)	53276 (45275-61537)	1154.78 (979.05-1336.7)	0.03 (0.03-0.03)	410666 (345357-489478)	13953.34 (11844.33-16363.61)	709916 (601857-832963)	14094.56 (11963.91-16527.66)	0.04 (0.04-0.04)	15357 (1887-34390)	524.86 (73.6-1151.54)	26629 (3831-57775)	527.91 (72.74-1147.49)	0.03 (0.03-0.04)
Croatia	51566 (43766-59938)	1090.73 (927.14-1261.35)	38937 (33672-45359)	1087.9 (919.35-1261.92)	-0.06 (-0.11--0.01)	724241 (619612-836750)	13686.48 (11588.51-15841.66)	615855 (536630-712211)	13584.21 (11643.92-15873.25)	-0.08 (-0.17-0)	27677 (6091-60964)	519.13 (106.63-1163.9)	23744 (5718-50876)	516.5 (107.38-1142.33)	-0.06 (-0.14-0.01)
Cuba	125447 (107206-144674)	1170.36 (998.02-1343.28)	110436 (94125-127574)	1166.08 (994.34-1339.1)	-0.01 (-0.01--0.01)	1635636 (1370554-1903808)	14183.48 (11958.04-16464.35)	1650955 (1411247-1920508)	14147.38 (11926.37-16422.57)	-0.01 (-0.01--0.01)	61077 (8158-133348)	530.34 (72.17-1154.01)	62238 (10331-135151)	529.47 (73.57-1156.15)	0 (0-0)
Cyprus	10620 (9059-12255)	1365.41 (1159.69-1572.75)	16331 (13796-19224)	1370.05 (1163.83-1578.47)	0.02 (0.02-0.02)	141129 (120240-166076)	17528.44 (14917.36-20657.07)	258628 (222725-302840)	17622.86 (14998.63-20771.18)	0.02 (0.02-0.03)	5272 (760-11564)	654.54 (93.44-1436.31)	9732 (1569-21129)	657.5 (94.31-1443.1)	0.03 (0.02-0.03)
Czechia	110160 (94025-127662)	1091.71 (927.97-1262.07)	98768 (84436-114611)	1087.51 (924.05-1257.72)	-0.01 (-0.02--0.01)	1507779 (1292391-1741031)	13692.29 (11594.08-15846.55)	1539807 (1332073-1769906)	13581.76 (11491.97-15721.88)	-0.03 (-0.03--0.03)	57383 (12385-125344)	518.13 (105.43-1155.65)	59097 (14004-127917)	514.44 (105.14-1155.09)	-0.02 (-0.03--0.02)
Côte d'Ivoire	152340 (127171-179381)	1161.46 (988.35-1337.39)	351632 (296614-409939)	1159.74 (986.81-1335.31)	-0.01 (-0.01--0.01)	1495297 (1248218-1809138)	14352.74 (12159.8-16949.75)	3662069 (3064787-4430446)	14352.21 (12164.87-16955.2)	-0.01 (-0.01-0)	54509 (6543-123649)	527.57 (78.45-1160.56)	135561 (17094-309402)	534.85 (80.4-1183.22)	0.04 (0.04-0.05)
Democratic People's Republic of Korea	211224 (177962-246595)	1010.39 (854.86-1178.57)	256683 (218693-300265)	990.52 (837.77-1156.7)	-0.07 (-0.07--0.07)	2513796 (2120814-2943252)	12076.54 (10187.21-14064.86)	3466450 (2946693-4050958)	11798.27 (9970.25-13731.96)	-0.08 (-0.08--0.08)	94105 (12675-209362)	451.6 (60.81-996.91)	130253 (18577-286442)	441.94 (58.91-975.29)	-0.07 (-0.07--0.07)
Democratic Republic of the Congo	401087 (333909-472573)	1016.18 (860.45-1179.37)	989146 (821014-1160241)	1010.12 (855.22-1172.65)	-0.02 (-0.02--0.02)	3957527 (3293568-4699867)	12452.58 (10536.03-14575.01)	9986213 (8297767-11847756)	12358.98 (10460.44-14466.19)	-0.03 (-0.03--0.03)	143994 (21316-331810)	457.56 (80.4-1003.91)	368337 (55579-835164)	459.87 (80.89-1005.63)	0.03 (0.02-0.03)
Denmark	58825 (50532-68144)	1248.31 (1061.97-1442.2)	61195 (52311-70308)	1266.41 (1076.02-1458.59)	0.05 (0.04-0.07)	820531 (705315-943790)	15003.7 (12808.35-17442.9)	906327 (783205-1046930)	15422.39 (13002.12-17919.21)	0.1 (0.07-0.13)	31220 (5404-67007)	566.78 (89.2-1228.91)	34567 (6198-72993)	581.19 (88.91-1273.05)	0.09 (0.07-0.11)
Djibouti	3337 (2792-3986)	744.25 (632.85-872.09)	9975 (8444-11752)	740.2 (628.89-867.21)	-0.02 (-0.02--0.01)	33084 (27627-39316)	8958.55 (7633.23-10449.48)	112429 (95034-131863)	8909.93 (7593.69-10390.05)	-0.01 (-0.02--0.01)	1260 (250-2888)	346.84 (79.85-758.53)	4318 (909-9685)	345.08 (79.29-752.63)	-0.01 (-0.01-0)
Dominica	895 (750-1048)	1165.01 (994.01-1338.09)	755 (645-868)	1166.34 (994.38-1339.57)	0 (0-0.01)	10021 (8365-11790)	14238.37 (12001.41-16527.26)	10063 (8509-11620)	14091.22 (11881.63-16357.54)	-0.03 (-0.04--0.03)	373 (47-816)	532.45 (73.67-1160.1)	376 (55-822)	524.43 (72.08-1148.05)	-0.04 (-0.04--0.04)
Dominican	93110	1182.41	129540	1169.62	-0.04 (-	998446	14322.8	1610281	14169.24	-0.05 (-	37067	535.63	59960	527.84	-0.05 (-

Republic	(77584-109182)	(1008.12-1355.91)	(110139-148561)	(997.25-1342.9)	0.05--0.04)	(824973-1186274)	(12078.42-16634.5)	(1356667-1869167)	(11947.02-16452.26)	0.05--0.04)	(4309-82913)	(72.33-1178.9)	(8091-130570)	(72.39-1147.47)	0.05--0.04)
Ecuador	109859 (93104-128000)	987.88 (845.68-1133.72)	184833 (156752-214825)	997.34 (844-1160.59)	0.05 (0.04-0.06)	1155515 (976992-1359991)	12076.41 (10330.52-13891.77)	2287823 (1915710-2701524)	12383.18 (10404.85-14597.05)	0.12 (0.1-0.14)	43095 (5928-95035)	454.82 (72.64-994.69)	85731 (13022-188128)	464.61 (72.25-1015.45)	0.11 (0.09-0.13)
Egypt	735591 (615773-859939)	1231.5 (1045.04-1432.87)	1448920 (1248614-1654365)	1273.1 (1102.23-1441.71)	0.15 (0.12-0.18)	7897165 (6580672-9382512)	15336.86 (12987.49-17980.81)	16377355 (14190621-18542240)	15670.29 (13673.87-17638.48)	0.1 (0.08-0.12)	309108 (50654-703810)	602.7 (108.47-1334.17)	641568 (106041-1401927)	615.17 (108.62-1331.54)	0.11 (0.08-0.13)
El Salvador	68121 (56322-80711)	1158.45 (982.46-1339.73)	76674 (65157-88377)	1165.35 (989.74-1347.1)	0.02 (0.01-0.03)	702533 (587545-837578)	14140.24 (12004.13-16583.65)	935408 (795016-1100747)	14345.72 (12175.94-16838.83)	0.05 (0.04-0.06)	25997 (3121-58066)	527 (72.76-1146.41)	34978 (4784-76320)	537.03 (74.74-1170.22)	0.07 (0.06-0.08)
Equatorial Guinea	4407 (3678-5175)	1024.76 (868.28-1189.48)	17118 (14233-20062)	996.13 (844.19-1156.08)	-0.11 (-0.11--0.1)	43939 (36601-52027)	12583.41 (10647.21-14733.35)	175468 (145549-209982)	12281.69 (10393.52-14346.4)	-0.09 (-0.1--0.08)	1604 (244-3592)	463.2 (80.68-999.49)	6476 (937-14750)	457.66 (79.48-992.37)	-0.04 (-0.06--0.02)
Eritrea	26818 (22389-31959)	756.79 (643.59-887.26)	53073 (44679-62388)	750.43 (638.04-878.67)	-0.03 (-0.03--0.03)	258685 (216673-306413)	9168.31 (7813.49-10686.2)	557908 (468792-657811)	9085.39 (7747.05-10593.4)	-0.03 (-0.04--0.03)	9679 (1918-21881)	349.29 (81.13-759.21)	21234 (4353-48468)	349.48 (79.94-765.07)	0.01 (0.01-0.01)
Estonia	15165 (12969-17569)	1013.43 (858.65-1168.55)	11362 (9695-13122)	1004.87 (851.11-1158)	-0.04 (-0.04--0.03)	216911 (187336-249922)	13084.29 (11243.66-15157.53)	180464 (156481-206210)	12900.48 (11078.06-14961.95)	-0.05 (-0.06--0.05)	8873 (2548-18422)	529.34 (144.3-1113.31)	7477 (2289-15186)	523.11 (142.17-1110.52)	-0.03 (-0.04--0.03)
Eswatini	8874 (7367-10540)	1025.71 (869.16-1190.96)	12866 (10768-15009)	1015.2 (860.07-1178.25)	-0.03 (-0.04--0.03)	84160 (69894-100560)	12571.43 (10640.44-14722.68)	139976 (116937-166126)	12480.77 (10567.53-14600.84)	-0.02 (-0.03--0.02)	3133 (447-7150)	473.72 (82.22-1038.11)	5130 (795-11639)	460.13 (81.17-1006.19)	-0.09 (-0.1--0.08)
Ethiopia	353873 (306198-411482)	692.93 (606-782.3)	818967 (706700-933192)	701.09 (612.31-794.29)	0.06 (0.05-0.07)	3372828 (2862058-3898962)	8324.72 (7169.4-9547.54)	8145628 (6882391-9470613)	8365.36 (7232.87-9566.16)	0.03 (0.02-0.04)	125805 (24848-282933)	314.79 (70.98-682.14)	301710 (53834-693818)	313.55 (63.99-683.83)	0 (-0.01-0.01)
Fiji	9735 (8208-11391)	1170.65 (1000.44-1355.25)	11120 (9489-12896)	1169.52 (999.66-1354.24)	0 (-0.01-0)	102546 (86146-122662)	14097.64 (11980.09-16492.76)	132120 (112105-154785)	14089 (11973.13-16481.84)	0 (-0.01-0)	3795 (422-8600)	522.44 (65.06-1165.84)	4887 (593-10905)	520.45 (63.89-1161.01)	0 (-0.01-0)
Finland	62921 (53348-73346)	1369.14 (1162.83-1577.26)	60345 (51402-69799)	1366.62 (1160.44-1574.69)	-0.01 (-0.01-0)	933312 (801825-1089898)	17569.6 (14955.48-20702.79)	959038 (826907-1120219)	17477.69 (14864.62-20595.43)	-0.02 (-0.02--0.02)	34967 (5599-75814)	654.16 (95.82-1434.69)	36012 (6290-77781)	649.91 (91-1426.19)	-0.02 (-0.02--0.01)
France	737962 (634494-852925)	1368.68 (1167.28-1574.92)	746314 (639835-855267)	1360.59 (1153.37-1566.45)	-0.03 (-0.04--0.02)	10605836 (9060937-12350852)	17613.31 (14955.69-20617.34)	11508602 (9881349-13376868)	17478.9 (14980.5-20463.93)	-0.04 (-0.05--0.03)	403421 (69442-867007)	666.5 (107.12-1443.81)	441401 (84488-934151)	661.31 (107.65-1429.12)	-0.02 (-0.04-0)
Gabon	10392 (8708-12181)	1012.43 (857.15-1175.16)	20010 (16776-23224)	1021.22 (864.72-1185.79)	0.03 (0.03-0.04)	107418 (89487-127530)	12379.35 (10483.12-14488.18)	219725 (184135-260555)	12471.35 (10554.8-14602.13)	0.03 (0.02-0.03)	3965 (619-8939)	460.79 (80.84-1001.32)	8134 (1285-18262)	463.95 (81.23-1012.4)	0.03 (0.03-0.04)
Gambia	12339 (10293-14561)	1167.11 (992.98-1344.13)	31125 (26222-36536)	1171.37 (996.65-1349.59)	0.02 (0.01-0.02)	120438 (100270-145835)	14416.44 (12227.67-17026.05)	319385 (267439-385394)	14530.02 (12317.95-17163.45)	0.03 (0.03-0.04)	4434 (549-10232)	536.61 (82.46-1190.7)	11750 (1467-26671)	538.73 (82.03-1193.37)	0.03 (0.02-0.03)
Georgia	59912 (51146-69366)	1106.2 (940.77-1286.69)	35320 (30330-40699)	1093.89 (929.53-1272.88)	-0.05 (-0.05--0.04)	783412 (668684-920771)	13711.08 (11647.59-16060.34)	508335 (434385-591390)	13553.71 (11509.17-15875.52)	-0.04 (-0.05--0.04)	29495 (5534-65951)	514.71 (92.98-1156.78)	19123 (3852-42048)	505.78 (89.98-1139.87)	-0.06 (-0.06--0.05)
Germany	977694 (836513-1118712)	1409.59 (1209.71-1616.81)	922855 (800531-1048309)	1421.37 (1215.13-1626.46)	0.03 (0-0.05)	16043947 (13833904-18769606)	18764.1 (16107.56-22011.93)	16539322 (14277980-19290792)	19203.72 (16546.88-22548.9)	0.04 (-0.02-0.11)	598931 (97014-1290623)	695.94 (101.9-1522.6)	615738 (104953-1316251)	707.4 (101.69-1567.8)	0.03 (-0.03-0.09)
Ghana	187798 (157451-220270)	1169.8 (995.51-1346.37)	435848 (368405-507261)	1172.96 (998.09-1350.62)	0.01 (0.01-0.02)	1889493 (1581780-2279085)	14525.57 (12313.44-17154.95)	4760614 (3992649-5745530)	14609.79 (12392.71-17245.18)	0.03 (0.02-0.03)	69602 (8918-160064)	539.75 (82.85-1205.24)	176558 (23095-399475)	545.15 (82.53-1210.74)	0.05 (0.04-0.05)
Greece	135691 (116570-	1406.59 (1203.67-	109983 (94131-	1401.01 (1190.61-	-0.01 (-0.02--	1989497 (1719296-	18297.01 (15663.05-	1868501 (1599645-	18254.82 (15571.69-	-0.01 (-0.01-0)	74209 (11176-	679.13 (93.52-	69986 (11835-	674.8 (91.73-	-0.01 (-0.02-0)

	155821)	1616.37)	125924)	1612.85)	0.01)	2318003)	21449.93)	2180108)	21589.54)		161284)	1486.44)	151360)	1475.63)	
Greenland	716 (612-842)	1225.11 (1047.94-1430.96)	643 (553-744)	1248.06 (1066.71-1455.72)	0.05 (0.05-0.06)	9558 (8158-11317)	15687.73 (13416.68-18403.44)	9519 (8159-11111)	16078.52 (13737.05-18845.21)	0.07 (0.07-0.08)	351 (48-773)	579.47 (85.54-1273.73)	353 (55-764)	593.26 (83.78-1292.44)	0.08 (0.08-0.09)
Grenada	1091 (905-1291)	1173.81 (1001.19-1347.18)	1143 (975-1312)	1162.45 (991.17-1335.53)	-0.04 (-0.05--0.03)	11482 (9564-13539)	14277.8 (12038.59-16578.38)	15340 (12959-17855)	14061.13 (11855.15-16317.93)	-0.05 (-0.05--0.05)	425 (52-944)	532.07 (72.8-1162.81)	573 (82-1247)	523.74 (70.66-1144.83)	-0.05 (-0.06--0.05)
Guam	1681 (1436-1971)	1158.46 (989.63-1344.03)	1755 (1507-2017)	1167.14 (997.43-1351.48)	0.02 (0.01-0.02)	19386 (16268-23084)	13901.92 (11812.31-16255.7)	23087 (19813-26899)	14056.48 (11946.69-16441.81)	0.03 (0.02-0.04)	725 (82-1633)	520.74 (63.3-1158.43)	861 (114-1905)	523.53 (66.32-1162.01)	0.02 (0.01-0.03)
Guatemala	109815 (89724-131682)	1157.13 (980.62-1338.88)	197762 (166240-229921)	1159.38 (983.1-1341.34)	0.01 (0-0.01)	1021511 (848201-1230670)	14048.2 (11927.56-16477.14)	2270498 (1917138-2689248)	14154.35 (12015.56-16599.61)	0.02 (0.02-0.03)	37498 (4287-83870)	519.75 (72.58-1132.74)	84011 (10846-186618)	524.95 (74.07-1153.4)	0.04 (0.04-0.05)
Guinea	71630 (60169-83285)	1175.96 (1000.9-1354.22)	172154 (144041-202089)	1177 (1001.7-1356.21)	0.01 (0.01-0.01)	730532 (612458-879830)	14611.41 (12397.95-17258.04)	1712116 (1431286-2067418)	14621.66 (12406.06-17274.28)	0.01 (0-0.01)	26920 (3593-60690)	542.2 (82.12-1189.63)	63179 (8033-144228)	544.31 (83.87-1208.01)	0.02 (0.02-0.02)
Guinea-Bissau	12760 (10615-15038)	1177.3 (1001.77-1356.53)	26750 (22432-31300)	1174.38 (999.22-1353.05)	-0.01 (-0.01-0)	123144 (102503-148770)	14630.83 (12412.02-17282.27)	270550 (226457-326698)	14614.35 (12398.05-17256.89)	0 (0-0)	4515 (563-10434)	541.33 (82.76-1199.23)	9969 (1256-22871)	542.99 (83.43-1208.93)	0.02 (0.01-0.02)
Guyana	10076 (8418-11797)	1178.18 (1004.56-1352.13)	9134 (7769-10473)	1176.93 (1003.63-1350.06)	0 (-0.01-0.01)	108745 (89938-129120)	14260.6 (12026.1-16563.55)	112238 (94272-130467)	14282.58 (12043.99-16581.7)	0.01 (0-0.01)	3994 (457-8868)	526.25 (71.39-1136.7)	4123 (552-9054)	524.86 (71.53-1151.34)	0 (0-0.01)
Haiti	82856 (68559-98141)	1186.26 (1011.59-1359.93)	164352 (138882-190388)	1181.07 (1007.28-1354.34)	-0.02 (-0.02--0.02)	840886 (697240-997077)	14355.29 (12109.86-16668.54)	1854945 (1552409-2174455)	14339.78 (12091.42-16650.64)	-0.01 (-0.01-0)	30695 (3574-68114)	527.3 (71.4-1145.61)	67951 (8311-147485)	527.01 (72.06-1142.83)	0 (-0.01-0.01)
Honduras	61398 (50267-73391)	1152.52 (977.02-1333.51)	127175 (106855-147813)	1159.23 (983.39-1340.72)	0.02 (0.02-0.03)	582364 (483496-701463)	14023.03 (11904.65-16447.16)	1451168 (1224291-1720656)	14156.32 (12016.93-16601.62)	0.04 (0.03-0.04)	21627 (2439-48839)	525.23 (73.13-1140.32)	53960 (6644-119697)	527.94 (72.37-1155.88)	0.03 (0.03-0.04)
Hungary	109686 (93632-127134)	1093.57 (929.72-1264.21)	89188 (76539-103432)	1090.02 (926.44-1260.3)	-0.01 (-0.01--0.01)	1534691 (1320053-1767845)	13733.24 (11632.25-15892.52)	1420159 (1231619-1633085)	13659.57 (11564.9-15808.12)	-0.02 (-0.02--0.02)	58343 (12705-127801)	518.43 (105-1155.34)	54588 (13037-117435)	517.97 (106.21-1147.24)	0.01 (0-0.01)
Iceland	3468 (2964-3998)	1368.43 (1162.25-1576.75)	4209 (3578-4868)	1366.05 (1159.56-1575.06)	-0.01 (-0.01-0)	45879 (39032-54126)	17516.49 (14897.32-20642.56)	62574 (53834-72797)	17439.61 (14827.64-20549.55)	-0.01 (-0.02--0.01)	1712 (237-3764)	654.06 (91.14-1435.76)	2352 (382-5110)	651.01 (92.73-1435.23)	-0.01 (-0.02--0.01)
India	11265220 (9867461-12808613)	1231.68 (1085.77-1382.46)	18411215 (16138211-20773298)	1232.92 (1080.89-1385.31)	-0.02 (-0.04-0)	118839678 (101287743-137816947)	14850.77 (12801.29-16953.83)	223124203 (189835220-254668065)	14909.22 (12770.95-16967.7)	-0.02 (-0.05-0.01)	4278545 (497814-9617566)	536.03 (69.56-1181.73)	8096331 (1015745-18217287)	540.99 (70.28-1209.87)	0.01 (-0.02-0.04)
Indonesia	2714314 (2366108-3098218)	1337.56 (1184.6-1506.87)	3827472 (3387835-4305612)	1331.48 (1179.22-1500.03)	-0.02 (-0.02--0.02)	28997586 (24554579-33630176)	16354.6 (14097.19-18846.47)	48937912 (41949667-56395686)	16269.49 (14018.67-18738.27)	-0.02 (-0.02--0.02)	1087126 (114878-2462407)	613.8 (72.38-1373.02)	1845660 (219017-4117373)	612.37 (72.39-1376.12)	0 (0-0.01)
Iran (Islamic Republic of)	811818 (705127-935084)	1280.77 (1127.97-1441.81)	1129462 (996103-1271389)	1297.77 (1147-1451.26)	0 (-0.05-0.05)	8073142 (6878696-9340092)	16084.72 (13857.54-18345.74)	15229950 (13250789-17521276)	16492.36 (14293.76-18891.95)	0.03 (-0.03-0.08)	313943 (50416-694070)	629.49 (117.09-1356.51)	593559 (109714-1275797)	641.56 (117.17-1405.74)	0.02 (-0.03-0.06)
Iraq	246424 (204990-293005)	1215.03 (1033.95-1403.85)	548611 (464094-635450)	1213.19 (1032.42-1401.82)	0 (-0.01-0)	2449660 (2033841-2928483)	15087.63 (12767.75-17726.2)	6291076 (5269687-7439825)	15038.58 (12718.44-17676.37)	-0.01 (-0.02--0.01)	94866 (14832-211599)	587.5 (109.34-1272.73)	244548 (40649-539194)	584.75 (104.24-1286.05)	-0.01 (-0.01-0)
Ireland	50448 (42507-58229)	1370 (1164.42-1578.01)	60724 (51513-70019)	1374.16 (1167.95-1582.77)	0.01 (0.01-0.01)	636831 (541901-749016)	17571.31 (14947.4-20713.85)	890679 (765138-1040070)	17632.3 (14997.65-20793.88)	0.01 (0.01-0.02)	23663 (3293-52067)	653.1 (91.15-1436.89)	33360 (5526-72825)	654.12 (94.34-1444.88)	0.01 (0-0.02)
Israel	70425 (59509-81491)	1373.47 (1167.47-1581.39)	125322 (106284-144415)	1370.09 (1163.93-1578.32)	-0.01 (-0.01--0.01)	859307 (730945-1011984)	17677.97 (15045.58-20842.07)	1646071 (1408912-1929110)	17572.11 (14949.33-20712.9)	-0.02 (-0.02--0.02)	32116 (4507-71118)	662.17 (95.58-1459.12)	61690 (9154-135395)	656.91 (92.78-1443.76)	-0.02 (-0.02--0.02)

Italy	768601 (685416-865153)	1489.38 (1319.89-1671.79)	673246 (599379-754102)	1494.64 (1329.2-1675.31)	0.12 (0.04-0.19)	11620968 (10095266-13397208)	19087.75 (16500.75-22036.12)	11636930 (10174340-13422450)	19244.27 (16729.3-22008.96)	0.19 (0.09-0.3)	433899 (60325-961472)	709.98 (89.55-1570.49)	438569 (68926-944342)	717.2 (89.24-1568.08)	0.21 (0.1-0.32)
Jamaica	30327 (25268-35582)	1178.83 (1005.27-1352.04)	32226 (27415-37035)	1174.29 (1001.3-1347.48)	-0.01 (-0.01--0.01)	329731 (273832-389683)	14296.59 (12058.02-16600.68)	427856 (361326-497956)	14226.31 (11995.1-16515.05)	-0.02 (-0.02--0.02)	12280 (1465-27319)	535.82 (72.23-1178.35)	16000 (2247-35030)	531.53 (72.93-1164.22)	-0.02 (-0.02--0.02)
Japan	1022690 (900054-1161317)	851.09 (747.16-964.47)	840431 (743168-946923)	873.6 (767.24-994.18)	0.13 (0.1-0.16)	14412657 (12488923-16423840)	10554.9 (9098.19-12108.87)	13831413 (12019194-15876263)	10818.4 (9282.5-12479.88)	0.12 (0.09-0.15)	562530 (134458-1204750)	406.38 (89.47-882.78)	545229 (141780-1160180)	415.34 (88.83-920.23)	0.11 (0.08-0.14)
Jordan	50821 (42326-60433)	1209.48 (1028.95-1396.71)	161821 (137114-186878)	1201.47 (1021.95-1386.51)	-0.03 (-0.04--0.01)	506087 (419614-605109)	15001.57 (12694.48-17629.65)	1920533 (1610797-2269549)	14843.49 (12538.68-17465.26)	-0.04 (-0.05--0.02)	19789 (2994-45057)	589.6 (105.58-1281.85)	75356 (12871-165494)	582.47 (105.03-1268.95)	-0.04 (-0.05--0.02)
Kazakhstan	186793 (157771-218047)	1103.18 (937.34-1283.82)	207339 (176414-239501)	1102.85 (937.2-1283.27)	0 (0-0)	2208492 (1864693-2599022)	13673.33 (11612.26-16009.82)	2626668 (2226459-3080362)	13656.47 (11600.64-15997.55)	0 (-0.01-0)	82031 (13935-187893)	509.53 (89.68-1153.05)	98007 (17958-220736)	508.64 (91.49-1148.41)	0.01 (0-0.01)
Kenya	188190 (162636-217387)	781.88 (682.55-882.79)	430357 (370938-491466)	781.63 (682.35-882.03)	0 (0-0)	1785902 (1498593-2079039)	9664.48 (8296.29-11069.43)	4547282 (3823894-5275131)	9671.53 (8306.01-11078.47)	0 (0-0)	67771 (13319-154084)	373.33 (84.52-801.94)	173533 (35639-387907)	373.46 (84.78-802.36)	0.02 (0.01-0.03)
Kiribati	914 (775-1068)	1176.48 (1005.79-1361.09)	1526 (1292-1774)	1178.73 (1007.64-1363.34)	0 (0-0.01)	9607 (8065-11479)	14209.55 (12081.03-16625.38)	16841 (14230-19906)	14255.59 (12121.54-16676.35)	0.01 (0.01-0.01)	352 (39-805)	521.36 (64.37-1155.94)	621 (72-1400)	525.75 (65.31-1171.11)	0.02 (0.02-0.03)
Kuwait	22742 (19082-26690)	1193.27 (1009.26-1373.49)	58544 (48839-69327)	1223.67 (1044.79-1415.02)	0.09 (0.07-0.12)	259298 (218673-310403)	14447.66 (12205.53-16959.46)	818072 (696347-967626)	14984.55 (12711.6-17585.31)	0.12 (0.09-0.15)	10135 (1537-22555)	565.92 (94.73-1239.15)	31838 (5527-68170)	581.9 (98.41-1263.92)	0.1 (0.07-0.13)
Kyrgyzstan	50755 (42718-59587)	1103.14 (937.39-1283.66)	78428 (66194-91556)	1101.59 (935.9-1281.91)	0 (-0.01-0)	557686 (471084-660228)	13647.07 (11592.47-15986.14)	915873 (772859-1077304)	13622.21 (11570.99-15961.84)	0 (0-0)	20689 (3433-48308)	509.67 (91.71-1161.45)	34132 (5870-77685)	509.39 (91.38-1144.07)	0.01 (0.01-0.02)
Lao People's Democratic Republic	56771 (47634-66967)	1293.3 (1101.23-1493.82)	100746 (85401-117161)	1284.51 (1093.45-1484.32)	-0.02 (-0.02--0.02)	571401 (479151-684675)	15905.93 (13514.69-18750.31)	1177650 (992323-1391695)	15770.99 (13402.65-18575.09)	-0.03 (-0.03--0.02)	21250 (2056-49134)	592.48 (64.97-1345.56)	44160 (4561-101957)	591.4 (65.33-1343.26)	0 (0-0)
Latvia	25387 (21638-29405)	1014.66 (859.73-1169.95)	15968 (13654-18399)	1008.08 (854.07-1161.98)	-0.02 (-0.03--0.02)	369427 (319314-425338)	13105.8 (11263.06-15183.71)	259186 (225126-296312)	12978.48 (11148.32-15044.14)	-0.03 (-0.04--0.03)	15133 (4348-31213)	530.3 (144.19-1123.41)	10761 (3322-21824)	526.11 (142.47-1108.17)	-0.01 (-0.02-0)
Lebanon	38337 (32558-44502)	1235.17 (1052.68-1427.54)	67777 (57667-79065)	1217.65 (1036.06-1406.01)	-0.05 (-0.06--0.04)	439886 (368688-518870)	15368.43 (13014.92-18048.78)	897713 (761686-1055679)	15238.44 (12901.08-17877.65)	-0.03 (-0.04--0.02)	17130 (2916-37548)	599.31 (106.61-1293.13)	34908 (6348-74604)	592.73 (106.06-1287)	-0.02 (-0.03--0.01)
Lesotho	16639 (13953-19596)	1034.65 (877.25-1201.7)	20889 (17580-24276)	1016.08 (860.82-1179.27)	-0.05 (-0.06--0.05)	167108 (139640-197571)	12765.62 (10825.68-14937.81)	230766 (193158-273822)	12483.69 (10571.75-14601.49)	-0.07 (-0.07--0.07)	6218 (956-13788)	478.64 (83.84-1032.84)	8443 (1319-18879)	458.7 (80.35-986.38)	-0.14 (-0.15--0.13)
Liberia	30109 (25219-35204)	1167.6 (993.68-1344.26)	70877 (59475-83007)	1164.51 (990.83-1341.19)	-0.03 (-0.04--0.03)	300557 (251961-362922)	14439.18 (12237.63-17056.28)	741785 (619373-896169)	14413.91 (12217.02-17028.92)	-0.03 (-0.04--0.03)	10824 (1396-24690)	524.45 (78.77-1165.21)	26959 (3441-60801)	526.6 (79.52-1157.7)	0 (-0.01-0)
Libya	57302 (47650-68134)	1204.21 (1024.3-1389.24)	87381 (74188-101703)	1217.46 (1036.25-1407.23)	0.01 (0-0.02)	571919 (474175-684355)	14808.36 (12493.55-17436.79)	1160232 (979112-1366496)	15082.94 (12758.48-17726)	0.04 (0.03-0.05)	22389 (3442-50736)	583.19 (104.09-1272.31)	45394 (8169-98360)	589.1 (107.62-1288.13)	0.02 (0-0.03)
Lithuania	34976 (29767-40748)	982.19 (835.95-1136.08)	22584 (19514-26192)	976.09 (830.75-1129.22)	-0.07 (-0.09--0.05)	478244 (411243-552567)	12350.74 (10556.05-14385.41)	358843 (310510-412286)	12244.96 (10458.5-14260.23)	-0.1 (-0.13--0.07)	19163 (5176-40076)	491.39 (125.94-1032.58)	14585 (4247-29905)	487.22 (121.55-1024.43)	-0.08 (-0.11--0.05)
Luxembourg	4612 (3938-5380)	1336.48 (1137.8-1539.37)	7394 (6284-8584)	1333.63 (1135.03-1536.23)	-0.02 (-0.04-0)	69656 (59801-81314)	16975.54 (14495.24-19890.96)	116292 (99994-136017)	16915.46 (14437.25-19829.86)	-0.04 (-0.08-0)	2660 (487-5695)	643.45 (107.15-1410.87)	4473 (872-9475)	641.87 (107.72-1397.18)	-0.02 (-0.03-0)
Madagascar	92741	755.2	233541	757.12	0.01	900837	9090.65	2351165	9122.41	0.01	33945	348.41	89243	351.38	0.04

	(77409-110318)	(642.51-884.82)	(196154-275910)	(644.2-887.27)	(0.01-0.01)	(755175-1066989)	(7739.17-10602.97)	(1969323-2787781)	(7768.58-10638.1)	(0.01-0.01)	(6716-77257)	(79.42-758.38)	(17765-204905)	(81.01-771.26)	(0.04-0.05)
Malawi	75769 (63362-89932)	757.16 (644.28-887.21)	162258 (135378-193982)	759.04 (646.1-889.84)	0.01 (0.01-0.01)	736834 (615991-873793)	9126.63 (7773.53-10643.49)	1593525 (1331647-1890613)	9155.39 (7800.08-10674.32)	0.02 (0.01-0.02)	27583 (5403-63436)	347.43 (79.14-759.91)	60327 (11598-140068)	351.95 (79.78-772.48)	0.08 (0.07-0.09)
Malaysia	225898 (188748-264486)	1192.91 (1010.27-1386.68)	399351 (337372-469503)	1206.73 (1018.18-1397.31)	0.06 (0.04-0.07)	2353279 (1961091-2759001)	14052.23 (11943.02-16246.76)	4880619 (4131861-5682693)	14260.88 (12084.97-16506.95)	0.07 (0.06-0.09)	88765 (9524-199855)	531.02 (63.67-1184.45)	184140 (21534-412521)	537.85 (63.87-1203.33)	0.07 (0.06-0.09)
Maldives	3002 (2516-3560)	1281.38 (1090.13-1481.61)	6783 (5692-8048)	1238.55 (1054.1-1436.81)	-0.12 (-0.15--0.1)	28855 (24182-34647)	15638.53 (13284.73-18430.24)	89867 (75947-106939)	14991.69 (12756.93-17610.83)	-0.15 (-0.18--0.11)	1072 (100-2476)	582.29 (64.57-1318.97)	3372 (363-7618)	562.08 (63.1-1277.98)	-0.11 (-0.15--0.07)
Mali	105034 (87925-122845)	1173.44 (998.55-1350.97)	303948 (252731-359857)	1170.64 (996.03-1348.55)	-0.01 (-0.01--0.01)	1041291 (869909-1254996)	14567.44 (12354.25-17207.14)	2924073 (2427810-3538302)	14516.45 (12306.67-17152.54)	-0.01 (-0.01--0.01)	38220 (5018-86559)	538.68 (82.79-1187.42)	107594 (13171-245929)	538.85 (81.55-1189.01)	0.01 (0.01-0.02)
Malta	4941 (4176-5723)	1369.33 (1163.11-1577.06)	4681 (3976-5478)	1363.74 (1158.17-1572.08)	-0.01 (-0.01--0.01)	68305 (58425-79801)	17606.89 (14987.3-20749.99)	77439 (66743-90120)	17448.6 (14837.97-20561.45)	-0.03 (-0.03--0.03)	2563 (388-5626)	657.9 (95.39-1450.18)	2921 (518-6308)	650.36 (92.31-1421.91)	-0.04 (-0.04--0.03)
Marshall Islands	584 (484-696)	1167.51 (998.11-1352.41)	709 (602-822)	1168.57 (998.68-1353.31)	0 (0-0)	5325 (4428-6380)	14042.82 (11929.05-16427.56)	8079 (6824-9509)	14055.76 (11941.82-16441.99)	0 (0-0)	197 (20-456)	520.36 (65.43-1158.79)	298 (35-665)	517.35 (65.27-1147.09)	-0.02 (-0.02--0.02)
Mauritania	25361 (21266-29748)	1170.55 (996.05-1348.14)	56332 (47320-66068)	1172.63 (997.86-1350.73)	0.01 (0.01-0.01)	255750 (214127-308561)	14535.6 (12321.07-17168.02)	576441 (481550-694368)	14556.42 (12339.11-17197.32)	0.01 (0.01-0.01)	9468 (1235-21542)	542.81 (82.72-1192.55)	21417 (2789-49077)	545.08 (81.72-1210.69)	0.02 (0.02-0.02)
Mauritius	15367 (13044-17932)	1284.9 (1093.72-1484.73)	15173 (13074-17516)	1284.94 (1093.72-1484.98)	0 (0-0)	178328 (150142-211137)	15792 (13422.1-18594.87)	220839 (187832-260053)	15779.33 (13410.81-18582.43)	0 (-0.01-0)	6643 (684-15153)	588.45 (65.65-1333.33)	8215 (997-18211)	586.53 (65.04-1335.26)	-0.01 (-0.01-0)
Mexico	1086589 (938023-1241864)	1144.96 (1006.07-1289.41)	1512449 (1327981-1700182)	1155.35 (1012.67-1299.96)	0.04 (0.04-0.05)	11354712 (9686129-13168337)	13943.99 (12149.15-16074.28)	19212341 (16538505-22337785)	14127.41 (12171.59-16374.51)	0.06 (0.05-0.07)	425068 (54457-942857)	525.11 (79.7-1131.67)	722310 (109702-1587845)	530.62 (79.92-1169.79)	0.05 (0.04-0.06)
Micronesia (Federated States of)	1317 (1093-1562)	1167.88 (998.83-1352.44)	1283 (1085-1486)	1168.36 (998.86-1352.91)	0 (-0.01-0)	12662 (10559-15091)	14061.59 (11945.64-16448.68)	14755 (12504-17329)	14078.19 (11964.66-16468.38)	0 (-0.01-0)	468 (50-1075)	521.11 (65.55-1163.23)	547 (65-1234)	520.87 (64.39-1167.45)	-0.01 (-0.01-0)
Monaco	319 (273-374)	1370.11 (1164.78-1578.86)	373 (320-428)	1375.74 (1168.8-1584.54)	0.02 (0.02-0.03)	5526 (4763-6462)	17652.23 (15023.48-20812.63)	6438 (5553-7524)	17639.08 (15006.93-20802.69)	0.01 (0-0.02)	210 (38-452)	659.99 (94.87-1441.89)	244 (46-521)	655.88 (96.05-1443.87)	0 (-0.01-0.01)
Mongolia	25298 (21124-29561)	1100.61 (935.19-1280.53)	37615 (31806-43578)	1101.2 (935.69-1281.42)	0 (0-0)	258937 (216885-309976)	13553.39 (11513.96-15890.73)	450789 (381219-530450)	13624.59 (11573.33-15964.34)	0.01 (0.01-0.02)	9543 (1430-22499)	505.32 (88.55-1143.82)	16773 (2852-38127)	507.53 (88.88-1146.58)	0.02 (0.02-0.02)
Montenegro	6922 (5893-7985)	1089.05 (925.49-1259.02)	6144 (5249-7111)	1087.93 (924.59-1257.63)	0 (-0.01-0)	88781 (75303-102729)	13653.84 (11562.22-15803.31)	90944 (78570-104954)	13632.03 (11541.95-15776.95)	0 (-0.01-0)	3381 (696-7609)	519.44 (105.64-1169.47)	3483 (786-7550)	516.27 (105.41-1149.24)	-0.01 (-0.01-0)
Morocco	338979 (285873-396245)	1228.59 (1045.83-1419.1)	460320 (392100-530291)	1223.77 (1041.86-1413.98)	-0.01 (-0.02--0.01)	3642047 (3028384-4324319)	15256.44 (12920.91-17911.01)	5872290 (4966992-6902142)	15201.02 (12873.44-17849.08)	-0.01 (-0.02--0.01)	141953 (23199-316929)	596.86 (107.41-1301.95)	228925 (41249-496937)	592.13 (106.43-1291.34)	-0.02 (-0.03--0.02)
Mozambique	104305 (87239-123862)	764.67 (650.42-896.79)	251716 (210192-299897)	762.48 (648.7-893.94)	-0.01 (-0.01--0.01)	1019646 (853453-1202171)	9216.02 (7848.14-10746.32)	2404741 (2008691-2847747)	9206.13 (7844.76-10734.38)	-0.01 (-0.01-0)	38096 (7770-86236)	348.95 (79.72-762.15)	89499 (17781-203346)	347.92 (82.25-754.65)	0 (-0.01-0.01)
Myanmar	568806 (481131-667103)	1290.45 (1098.66-1490.87)	750889 (639998-868554)	1291.88 (1100.14-1491.78)	0 (0-0.01)	6117704 (5137282-7291565)	15862.42 (13478.83-18691.92)	9253224 (7825988-10929907)	15924.77 (13532.65-18763.28)	0.01 (0.01-0.02)	227472 (23173-523083)	590.53 (65.61-1342.85)	346238 (37501-792651)	595.18 (65.07-1356.83)	0.04 (0.03-0.05)
Namibia	15267 (12658-	1017.4 (861.53-	26802 (22482-	1017.47 (861.63-	0 (0-0)	153738 (127764-	12454.09 (10540.66-	298885 (250472-	12478.7 (10562.03-	0 (0-0.01)	5719 (854-12998)	467.97 (82.48-	11094 (1764-	465.84 (82.6-	0 (0-0)

	17975)	1181.19)	31193)	1181.03)		182722)	14580.2)	354100)	14603.91)			1017.36)	24924)	1017.57)	
Nauru	126 (105-147)	1166.06 (995.78-1350.76)	139 (118-162)	1171.75 (1001.93-1355.84)	0.01 (0.01-0.02)	1287 (1081-1538)	13963.68 (11855.19-16333.14)	1500 (1263-1790)	14158.58 (12037.72-16565.56)	0.04 (0.04-0.05)	48 (5-109)	518.44 (64.27-1155.61)	55 (6-126)	522.68 (65-1168.04)	0.03 (0.02-0.03)
Nepal	262329 (219541-306734)	1264.39 (1073.76-1460.98)	418832 (356194-488554)	1272.83 (1086.72-1469)	0.03 (0.02-0.03)	2682905 (2238419-3199663)	15558.3 (13173.32-18335.58)	4977232 (4192666-5913099)	15717.9 (13261.38-18538.76)	0.04 (0.03-0.04)	96231 (10287-217721)	559.27 (68.67-1240.87)	180025 (20902-408340)	568.37 (70.18-1262.36)	0.07 (0.06-0.07)
Netherlands	183749 (157376-211415)	1305.15 (1118.44-1507.77)	188016 (162113-215993)	1351.72 (1157.08-1557.38)	0.19 (0.14-0.24)	2577262 (2225844-2984033)	16015.92 (13799.68-18510.9)	2944901 (2542077-3405006)	17015.11 (14543.35-19738.31)	0.33 (0.25-0.41)	96125 (13791-210597)	595.52 (80.47-1312.45)	109685 (16353-242319)	628.54 (78.22-1410.88)	0.33 (0.25-0.41)
New Zealand	38464 (33958-43887)	1129.45 (998.03-1278.47)	52692 (46751-59964)	1125.09 (994.52-1273.33)	0 (0-0.01)	499988 (431850-574761)	13965.07 (12043.65-16036.08)	752958 (655401-861494)	13912.65 (11994.41-15968.85)	0 (0-0.01)	18486 (3273-40722)	515.91 (90.89-1138.2)	28202 (5415-61064)	517.01 (90.62-1139.12)	0.02 (0.02-0.03)
Nicaragua	51565 (42183-61605)	1157.86 (981.58-1339.23)	81259 (68833-94047)	1148.34 (973.51-1329.98)	-0.03 (-0.03--0.03)	486708 (403841-586878)	14100.7 (11970.76-16536.04)	961782 (815164-1134045)	14032.73 (11909.89-16453.73)	-0.01 (-0.01--0.01)	18005 (1987-40754)	526.62 (73.31-1152.76)	35919 (4734-78659)	524.9 (73.49-1144.11)	0 (-0.01-0)
Niger	99234 (82117-116874)	1170.99 (996.46-1348.95)	315826 (259822-376236)	1170.77 (996.38-1348.6)	0 (0-0)	936238 (778537-1131724)	14486.96 (12280.1-17113.98)	2919359 (2418795-3530584)	14539.47 (12319.59-17178.44)	0.01 (0.01-0.02)	34411 (4282-78794)	537.87 (82.45-1186.75)	108020 (12824-247685)	543.59 (80.33-1205.56)	0.05 (0.04-0.05)
Nigeria	1161788 (1013463-1329980)	1223.59 (1085.94-1376.83)	3098015 (2705925-3559712)	1225.57 (1082.07-1378.99)	0 (-0.01-0.01)	11887143 (10080778-13825964)	15070.33 (12928.73-17357.67)	31067867 (26146877-36096317)	15259.08 (13066.29-17700.18)	0.04 (0.03-0.05)	436643 (59178-982148)	558.25 (85.32-1223.83)	1149585 (154650-2624356)	569.67 (89.67-1257.77)	0.08 (0.07-0.09)
Niue	27 (23-32)	1168.21 (998.64-1353.7)	19 (16-22)	1168.03 (998.91-1352.89)	0 (0-0)	302 (256-353)	14095.97 (11987.69-16486.29)	244 (210-284)	14087.91 (11977.66-16480.21)	-0.01 (-0.01-0)	11 (1-25)	523.96 (63.04-1158.05)	9 (1-20)	520.7 (65.35-1153.01)	-0.02 (-0.02--0.01)
North Macedonia	22228 (18873-25657)	1090.25 (926.51-1260.34)	21804 (18578-25492)	1084.44 (921.19-1254.1)	-0.02 (-0.02--0.02)	282341 (239021-326757)	13635.23 (11540.95-15781.91)	331181 (284825-383702)	13534.57 (11451.17-15668.78)	-0.03 (-0.03--0.03)	10724 (2191-23630)	517.47 (105.41-1140.29)	12682 (2942-27529)	512.39 (106.6-1141.21)	-0.02 (-0.03--0.02)
Northern Mariana Islands	580 (492-686)	1164.3 (993.87-1350.61)	560 (477-648)	1160.96 (992.39-1344.63)	-0.01 (-0.03-0.01)	6835 (5704-8171)	13845 (11755.57-16187.69)	7251 (6207-8416)	13954.79 (11851.33-16318.41)	0.01 (-0.01-0.03)	255 (28-576)	517.16 (65.73-1153.54)	271 (36-599)	519.07 (65.8-1152.68)	0 (-0.02-0.02)
Norway	52662 (47099-59196)	1336.83 (1191.59-1501.81)	65740 (58406-73788)	1425.49 (1259.66-1603.91)	0.35 (0.27-0.43)	731991 (639670-841578)	16449.95 (14306.96-18921.08)	980507 (852994-1124376)	17700.09 (15229.47-20234.72)	0.34 (0.26-0.42)	27023 (3906-59070)	605.79 (81.9-1338.14)	36469 (5566-79611)	653.12 (84.81-1449.64)	0.35 (0.27-0.43)
Oman	25311 (21160-29639)	1150.65 (975.27-1331.35)	58142 (48930-68501)	1157.15 (980.81-1338.24)	0 (-0.03-0.03)	257358 (213872-308591)	14075.91 (11888.22-16515.77)	723114 (604453-855561)	14115.28 (11919.02-16567.27)	0.01 (-0.02-0.04)	10105 (1605-22297)	555.42 (101.23-1210.33)	28571 (4861-60732)	557.1 (101.26-1218.38)	0.02 (-0.01-0.05)
Pakistan	1434881 (1238380-1637267)	1218.04 (1072.69-1360.86)	3125797 (2729574-3528274)	1223.13 (1077.41-1366.83)	0 (-0.01-0.01)	14026039 (11831211-16136204)	14590.27 (12506.39-16711.86)	33211064 (27967358-38280811)	14570.79 (12494.14-16671.84)	-0.03 (-0.05--0.02)	504195 (53643-1155356)	526.45 (64.99-1181.42)	1178392 (107593-2697294)	517.34 (52.91-1172.44)	-0.1 (-0.12--0.07)
Palau	193 (164-225)	1166.49 (996.81-1351.27)	198 (169-229)	1146.66 (979.24-1329.86)	-0.05 (-0.07--0.04)	2187 (1842-2594)	14038.38 (11931.12-16420.76)	2775 (2378-3228)	13739.06 (11666.04-16056.91)	-0.06 (-0.08--0.04)	81 (9-183)	521.61 (64.23-1162.18)	103 (14-226)	508.56 (64.34-1135.75)	-0.08 (-0.09--0.06)
Palestine	27129 (22468-32304)	1225.69 (1042.87-1414.96)	69981 (59028-81676)	1219.89 (1038.24-1409.58)	0 (-0.01-0)	264017 (219075-315063)	15334.33 (12978.89-17984.54)	763181 (634681-905754)	15147.2 (12823.09-17790.44)	-0.03 (-0.04--0.02)	10278 (1553-23552)	600.71 (107.7-1311.45)	29677 (4857-65792)	590.22 (106.13-1285.05)	-0.04 (-0.04--0.03)
Panama	29845 (24948-35019)	1142.91 (968.64-1323.73)	49189 (41717-56690)	1141.54 (967.47-1322.12)	0 (0-0)	327634 (275883-390571)	13857.73 (11763.35-16260.97)	606863 (514704-712399)	13876.05 (11777.39-16271.48)	0.01 (0.01-0.01)	12262 (1569-27514)	521.36 (74.81-1144.14)	22789 (3207-49539)	520.94 (72.69-1131.98)	0.01 (0-0.01)
Papua New Guinea	50954 (42836-59920)	1164.48 (995.45-1348.27)	128588 (109084-149593)	1165.81 (996.08-1349.71)	0 (0-0)	517998 (432698-618860)	14007.4 (11900.13-16383.85)	1388464 (1168290-1655046)	14021.85 (11915.81-16401.32)	0 (0-0)	19010 (2074-43594)	515.24 (64.04-1140.89)	51278 (5870-115623)	518.1 (64.71-1150.47)	0.02 (0.01-0.02)

Paraguay	67540 (55775-80215)	1443.92 (1223.62-1681.93)	107102 (91056-124513)	1440.78 (1220.55-1678.46)	-0.01 (-0.01--0.01)	682377 (570255-819973)	17508.16 (14663.62-20724.14)	1308475 (1094481-1547870)	17467.73 (14631.75-20671.43)	-0.01 (-0.01--0.01)	25120 (2082-57893)	645.53 (65.84-1456.05)	47963 (4685-109466)	640.61 (66.65-1452.92)	-0.01 (-0.02--0.01)
Peru	184220 (156427-214617)	777.26 (666.6-898.88)	303758 (257133-353056)	829.96 (700.25-963.55)	0.23 (0.15-0.3)	1820243 (1556593-2116088)	8739.19 (7608.1-10076.06)	3616808 (3050028-4201121)	9645.45 (8132.19-11190.36)	0.35 (0.24-0.45)	68953 (12735-161642)	336.69 (71.68-753.5)	137935 (27416-307041)	368.08 (73.78-818.06)	0.32 (0.23-0.42)
Philippines	909034 (792826-1039584)	1335.34 (1182.56-1504.43)	1599831 (1407392-1807966)	1330.93 (1178.86-1499.44)	-0.01 (-0.01--0.01)	9411171 (7940055-10961993)	16315.33 (14061.17-18795.51)	18754473 (16081015-21707058)	16273.79 (14024.28-18738.94)	-0.01 (-0.01--0.01)	350823 (35798-797014)	609.05 (71.8-1361.93)	702201 (79325-1581758)	609.09 (72.16-1363.32)	0.01 (0.01-0.01)
Poland	433406 (383184-489853)	1134.53 (1002.28-1280.63)	382967 (339974-433272)	1131.81 (999.78-1277.76)	-0.01 (-0.01--0.01)	5697346 (4956976-6487128)	14325.36 (12441.12-16385.69)	5973361 (5222976-6772298)	14258.61 (12376.5-16315.58)	-0.02 (-0.02--0.02)	216725 (47743-469198)	543.28 (116.79-1184.53)	230606 (56408-492545)	543.31 (117.09-1189.22)	0.01 (0-0.01)
Portugal	134223 (113983-154662)	1376.31 (1169.65-1585.21)	112245 (95860-129640)	1375.15 (1168.59-1583.65)	0 (0-0)	1878689 (1610478-2196130)	17715.06 (15079.46-20882.66)	1903085 (1643638-2225482)	17692.88 (15059.18-20857.53)	0 (0-0)	69945 (10874-152470)	657.19 (94.38-1440.79)	71559 (12910-153421)	656.01 (93.82-1444.85)	0.01 (0.01-0.02)
Puerto Rico	43442 (36940-49826)	1181.12 (1007.59-1354.09)	31729 (27325-36148)	1177.03 (1003.99-1349.96)	-0.01 (-0.01--0.01)	530326 (446503-615716)	14366.59 (12115.24-16677.17)	482950 (412713-559392)	14317.03 (12072.19-16620.52)	-0.01 (-0.01--0.01)	19843 (2674-43395)	537.73 (73.41-1175.74)	18151 (2996-39207)	535.12 (72.06-1167.51)	-0.01 (-0.01--0.01)
Qatar	5346 (4503-6322)	1121.23 (950.06-1301.19)	33946 (28048-40819)	1120.11 (950.19-1300.24)	-0.11 (-0.15--0.06)	65014 (54445-77256)	13522.66 (11433.39-15868.63)	476079 (396810-565821)	13503.71 (11414.62-15842.51)	-0.12 (-0.17--0.07)	2574 (444-5614)	535.69 (99.1-1173.65)	18893 (3412-40348)	532.8 (97.08-1153.57)	-0.12 (-0.16--0.08)
Republic of Korea	474056 (398314-554386)	966.46 (819.58-1121.32)	401658 (344821-463929)	937.81 (790.34-1097.69)	-0.18 (-0.21--0.15)	5962457 (4992520-7033238)	12363.14 (10419.44-14454.64)	6685316 (5762127-7766628)	11812.12 (9986.5-13862.85)	-0.28 (-0.33--0.23)	220660 (36626-511187)	460.45 (81.37-1043.8)	256226 (55454-555437)	444.1 (81.27-1003.56)	-0.24 (-0.28--0.2)
Republic of Moldova	45380 (38468-53005)	1018.76 (862.94-1174.84)	33026 (28001-38566)	1008.23 (854.09-1162.23)	-0.04 (-0.04--0.03)	594525 (511136-689173)	13131.86 (11283.54-15223.5)	527387 (457303-607970)	12971.64 (11140.99-15039.45)	-0.04 (-0.04--0.04)	24151 (6654-51482)	532.24 (144.89-1138.65)	21847 (6673-44584)	526.37 (144.95-1120.24)	-0.02 (-0.03--0.02)
Romania	251777 (214575-290117)	1092.36 (928.51-1262.81)	178064 (153122-206093)	1087.77 (924.29-1257.83)	-0.02 (-0.02--0.01)	3354314 (2857983-3879653)	13679.57 (11579.83-15832.29)	2766761 (2398064-3185737)	13609.33 (11518.75-15752.39)	-0.02 (-0.02--0.02)	127606 (27010-282196)	517.54 (104.55-1158.87)	106719 (25311-228724)	517.26 (105.21-1151.94)	0.01 (0-0.01)
Russian Federation	1608140 (1419750-1816351)	1089.5 (960.71-1224.03)	1433751 (1272612-1615350)	1088.3 (959.74-1222.56)	0 (-0.01-0.01)	23143655 (20066885-26450398)	14421.34 (12495.15-16469.75)	22799068 (19758994-25864414)	14379.09 (12456.59-16421.26)	0.02 (-0.01-0.05)	957121 (292187-1945141)	591.25 (172.45-1225.11)	955154 (306141-1915842)	589.48 (172.15-1220.02)	0.05 (0-0.1)
Rwanda	56079 (46834-66617)	759.29 (645.67-889.11)	108159 (91152-127419)	758.73 (645.22-888.94)	-0.01 (-0.03-0)	537844 (451051-636877)	9173.24 (7818.69-10697.43)	1140901 (957536-1346121)	9178.77 (7823.44-10701.57)	-0.01 (-0.02-0)	20305 (3985-46592)	352.29 (81.46-768.64)	43387 (8833-97525)	353.02 (81.18-772.64)	0.02 (0.02-0.03)
Saint Kitts and Nevis	519 (435-608)	1176.14 (1003.07-1349.4)	630 (537-725)	1171.39 (998.61-1344.92)	-0.01 (-0.01--0.01)	5726 (4783-6746)	14288.07 (12049.74-16589.12)	9083 (7693-10535)	14177.84 (11957.03-16463.16)	-0.02 (-0.03--0.02)	212 (26-469)	531.29 (71.7-1159.55)	340 (52-744)	527.92 (74.09-1149.37)	-0.01 (-0.02--0.01)
Saint Lucia	1783 (1480-2106)	1181.32 (1007.49-1354.88)	1882 (1607-2156)	1168.76 (996.67-1341.85)	-0.04 (-0.04--0.04)	18862 (15618-22358)	14351.03 (12103.73-16664.92)	27077 (22958-31433)	14161.11 (11938.88-16439.21)	-0.05 (-0.05--0.04)	697 (81-1560)	533.27 (71.31-1169.49)	1010 (156-2191)	525.64 (72.45-1141.37)	-0.04 (-0.05--0.04)
Saint Vincent and the Grenadines	1452 (1203-1718)	1172.04 (999.5-1345.66)	1272 (1084-1459)	1168.29 (995.95-1341.62)	-0.01 (-0.01-0)	15142 (12545-17985)	14223.32 (11992.77-16515.56)	16770 (14211-19359)	14107.49 (11893.93-16375.82)	-0.02 (-0.03--0.02)	561 (66-1252)	530.96 (72.82-1159.57)	625 (94-1363)	524.41 (73.02-1138.54)	-0.03 (-0.04--0.03)
Samoa	2117 (1759-2502)	1162.04 (993.42-1345.39)	2582 (2181-3014)	1166.92 (997.23-1351.29)	0.01 (0.01-0.01)	20924 (17426-24891)	14009.96 (11911.18-16384.13)	27775 (23531-32905)	14039.23 (11929.11-16423.09)	0.01 (0.01-0.01)	775 (85-1779)	519.92 (64.55-1160.22)	1027 (119-2308)	518.98 (64.47-1159.56)	0 (-0.01-0)
San Marino	307 (263-356)	1374.27 (1168.18-1583.7)	352 (300-404)	1374.79 (1169.05-1583.39)	0 (-0.01-0)	4500 (3860-5269)	17609.99 (14974.45-20759.82)	5878 (5078-6881)	17738.7 (15098-20908.45)	0.02 (0.02-0.03)	169 (25-368)	657.94 (91.78-1450.11)	222 (39-478)	660 (94-1449.18)	0.02 (0.01-0.03)
Sao Tome and	1534	1171.38	2810 (2369-	1166.22	-0.02 (-	14811 (12311-	14553.95	30685	14456.37	-0.03 (-	550 (71-	545.61	1145 (153-	542.03	-0.02 (-

Principe	(1272-1819)	(996.61-1350.14)	3287)	(992.32-1343.77)	0.02--0.02)	17829)	(12342.4-17190.51)	(25661-37054)	(12248.45-17076.1)	0.03--0.02)	1260)	(82.23-1215.68)	2594)	(83.97-1200.84)	0.02--0.02)
Saudi Arabia	206079 (172426-243057)	1160.85 (988.23-1339.65)	463330 (389641-548894)	1162.99 (990.3-1342.8)	-0.01 (-0.02-0)	2092839 (1745746-2504269)	14067.54 (11978.05-16528.09)	6167269 (5209389-7309686)	14115.56 (12026.1-16592.17)	-0.01 (-0.02--0.01)	82139 (13308-182683)	554.57 (102.46-1194.68)	243297 (44017-519225)	555.39 (101.75-1209.55)	-0.01 (-0.02--0.01)
Senegal	95288 (79403-112412)	1175.17 (999.97-1354.24)	202256 (170806-236624)	1166.97 (993.03-1343.88)	-0.02 (-0.03--0.02)	923324 (768387-1115483)	14563.94 (12347.12-17207.36)	2124776 (1778975-2557699)	14508.51 (12298.2-17139.35)	-0.01 (-0.01--0.01)	33875 (4295-77346)	539.7 (82.35-1192.29)	78588 (10290-178784)	540.26 (83.42-1194.15)	0.01 (0.01-0.01)
Serbia	103046 (87647-119144)	1092.09 (928.31-1263.02)	86815 (74218-100956)	1080.23 (917.37-1248.49)	-0.03 (-0.04--0.02)	1403078 (1201275-1623871)	13646.06 (11574.3-15804.9)	1325082 (1147909-1527772)	13545.29 (11487.32-15681.86)	-0.02 (-0.03--0.01)	53713 (12312-118362)	518.8 (112.05-1162.11)	50906 (12098-109628)	514.68 (107.95-1136.79)	-0.01 (-0.02-0)
Seychelles	995 (843-1164)	1283.14 (1092.07-1483.24)	1298 (1114-1504)	1270.45 (1080.14-1469.78)	-0.04 (-0.05--0.04)	11199 (9418-13333)	15753.17 (13391.09-18543.92)	17551 (14923-20745)	15527.62 (13203.68-18258.36)	-0.06 (-0.07--0.05)	420 (43-969)	591.77 (65.82-1351.52)	657 (79-1469)	580.57 (65.89-1318.21)	-0.06 (-0.07--0.06)
Sierra Leone	50317 (42409-58842)	1172.23 (997.51-1349.18)	113302 (95908-132380)	1169.24 (994.89-1347.25)	-0.02 (-0.02--0.01)	522798 (439177-629720)	14525.13 (12315.56-17157.74)	1188125 (996359-1432068)	14478.41 (12271.57-17107.17)	-0.02 (-0.03--0.02)	19200 (2578-43534)	537.72 (83.22-1190.03)	43907 (5566-100909)	539.02 (81.61-1201.44)	0 (0-0.01)
Singapore	24062 (20554-28820)	719.67 (614.38-849.34)	40277 (33891-48620)	803.97 (670.81-946.86)	0.41 (0.3-0.52)	288098 (247365-340959)	8316.27 (7170.33-9725.85)	603543 (519625-703072)	9471.09 (8033.57-11042.2)	0.48 (0.35-0.62)	11424 (2667-25233)	332.18 (81.51-725.17)	24170 (6063-52096)	370.02 (81.41-823.31)	0.4 (0.29-0.51)
Slovakia	58083 (49697-67295)	1093.22 (929.32-1263.58)	52397 (44650-61229)	1088.42 (924.96-1258.68)	-0.02 (-0.02--0.02)	753612 (640349-870490)	13717.8 (11617.62-15875.1)	810276 (699863-935456)	13618.05 (11526.24-15761.99)	-0.03 (-0.03--0.02)	28583 (5960-63012)	519.33 (106.64-1154.13)	31140 (7227-67224)	516.94 (105.43-1156.64)	-0.01 (-0.01--0.01)
Slovenia	21242 (18075-24747)	1090.55 (926.9-1261.33)	18844 (16116-21878)	1084.46 (921.24-1254.18)	-0.02 (-0.03--0.02)	291567 (248754-337082)	13685.69 (11587.75-15841.92)	298681 (258728-343964)	13531.47 (11447.95-15665.17)	-0.04 (-0.04--0.04)	11091 (2412-24307)	518.35 (107.91-1156.48)	11517 (2766-24686)	513.87 (104.38-1148.52)	-0.03 (-0.03--0.02)
Solomon Islands	4214 (3495-5004)	1165.92 (996.21-1350.3)	8534 (7185-9964)	1170.11 (999.97-1354.24)	0.01 (0.01-0.01)	40323 (33445-48057)	13993.27 (11884.4-16366.41)	90519 (76291-107912)	14096 (11980.4-16491.08)	0.03 (0.03-0.03)	1492 (158-3422)	519.27 (65.29-1161.45)	3352 (377-7611)	522.13 (64.03-1164.41)	0.02 (0.02-0.03)
Somalia	61478 (51149-73409)	748.23 (635.95-876.71)	168967 (140813-201457)	751.12 (638.48-879.61)	0 (0-0.01)	581887 (487453-685808)	9053.13 (7716.49-10554.75)	1599393 (1335019-1895442)	9098.36 (7757.78-10608.17)	0 (0-0.01)	21864 (4283-50117)	346.73 (80.57-754.9)	60302 (11477-138449)	349.03 (79.38-760.45)	0.02 (0.02-0.03)
South Africa	418958 (367269-477858)	1056.21 (939.73-1190.52)	625048 (553112-708205)	1051.35 (935.49-1185.01)	-0.02 (-0.02--0.02)	4535292 (3868035-5265095)	13043.67 (11258.85-14997.35)	7754002 (6677176-8973176)	12990.88 (11216.6-14931.63)	-0.02 (-0.02--0.01)	168624 (27526-372291)	488.89 (88.91-1059.99)	286549 (49986-622369)	480.97 (86.86-1039.31)	-0.05 (-0.06--0.05)
South Sudan	45803 (38255-54545)	744.32 (633.04-872.5)	77326 (64146-92009)	757.61 (644.35-888.81)	0.05 (0.05-0.06)	444475 (371396-527651)	8911.89 (7586.5-10397.79)	759706 (635942-892835)	9129.91 (7771.01-10644.15)	0.08 (0.07-0.08)	16689 (3374-38478)	339.27 (80.26-740.01)	28640 (5591-65408)	347.88 (78.23-759.56)	0.08 (0.08-0.09)
Spain	521059 (445381-604194)	1395.06 (1188.88-1609.1)	502945 (431506-580753)	1393.83 (1188.05-1607.19)	-0.01 (-0.02-0)	7469970 (6461183-8764842)	18290 (15772.71-21523.55)	8537878 (7350314-10025982)	18266.01 (15746.95-21477)	0 (-0.01-0.01)	282710 (45197-606535)	689.4 (104.11-1484.11)	327243 (62354-698496)	688.24 (105.5-1469.69)	0.03 (0.01-0.04)
Sri Lanka	240109 (203710-279978)	1285.6 (1094.36-1485.74)	280660 (240570-323340)	1290.9 (1099.14-1491)	0.01 (0.01-0.02)	2715644 (2281879-3219901)	15768.45 (13398.97-18578.29)	3716037 (3161618-4382235)	15890.54 (13502.71-18725.41)	0.03 (0.02-0.03)	101276 (10372-232559)	588.28 (65.43-1340.67)	138579 (16225-312066)	592.23 (65.81-1349.2)	0.03 (0.03-0.04)
Sudan	267086 (223161-316310)	1231.3 (1048.79-1423.34)	597056 (502304-696718)	1226.29 (1044.55-1417.2)	-0.01 (-0.01-0)	2693502 (2239447-3207687)	15255.18 (12922.03-17915.73)	6389370 (5319941-7585089)	15216.83 (12893.39-17871.85)	0 (-0.01-0)	104607 (16528-234592)	595.84 (107.48-1294.78)	248998 (39573-559046)	595.27 (107.58-1305.82)	0 (0-0.01)
Suriname	4904 (4121-5726)	1172.68 (999.96-1346.69)	6680 (5706-7675)	1172.83 (1000.37-1345.29)	0 (-0.01-0)	54684 (45587-64360)	14190.74 (11962.16-16476.73)	85372 (72233-98713)	14248.98 (12015.96-16537.07)	0.01 (0.01-0.02)	2029 (245-4471)	529.04 (70.9-1159.49)	3161 (465-6928)	526.65 (73.94-1147.9)	0 (-0.01-0)
Sweden	105124 (93626-	1411.09 (1255.74-	121036 (107678-	1409.7 (1249.25-	0 (0-0)	1578560 (1377444-	17833.45 (15392.41-	1846704 (1613905-	17826.53 (15449.25-	0 (0-0.01)	58680 (8549-	659.2 (84.95-	68595 (10307-	657.82 (84.47-	-0.01 (-0.02-0)

	117994)	1585.76)	135702)	1585.26)		1808491)	20357.43)	2112038)	20442.69)		127851)	1448.06)	147742)	1464.24)	
Switzerland	79697 (68330-92841)	1279.54 (1091.83-1472.84)	93026 (79679-107401)	1284.81 (1090.41-1475.61)	0.01 (0.01-0.02)	1155889 (994451-1329069)	15679.92 (13327.77-18192.16)	1444817 (1250524-1670107)	15818.27 (13505.05-18301.38)	0.03 (0.03-0.04)	43357 (7331-93089)	584.52 (90.5-1269.5)	54637 (10398-117888)	590.17 (93.02-1295.68)	0.04 (0.04-0.05)
Syrian Arab Republic	173789 (143697-207298)	1220.85 (1039.12-1410.83)	178974 (152659-208485)	1260.82 (1075.22-1456.37)	0.09 (0.06-0.13)	1672346 (1388698-1999259)	15128.19 (12806.89-17772.16)	2274061 (1928132-2665064)	15609.45 (13220.45-18351.38)	0.09 (0.05-0.12)	65229 (10019-148109)	593.86 (108.01-1293.84)	88757 (15805-193616)	606.42 (108.11-1315.1)	0.06 (0.04-0.08)
Taiwan (Province of China)	227057 (194959-261371)	1037.22 (900.94-1188.4)	220839 (188684-255844)	1055.29 (897.89-1221.93)	0.07 (0.05-0.09)	2678522 (2333421-3072927)	12469.03 (10901.69-14283.52)	3485294 (2990346-4096089)	13042.93 (11125.84-15421.62)	0.18 (0.15-0.22)	100111 (12283-225612)	466.11 (59.99-1041.32)	129941 (18549-286886)	485.07 (61.65-1068.88)	0.17 (0.14-0.2)
Tajikistan	60922 (51026-71345)	1101.24 (936.06-1281.17)	116549 (98111-136525)	1097 (931.95-1276.72)	-0.02 (-0.02--0.01)	623925 (523153-746875)	13574.15 (11531.5-15913.33)	1322079 (1116233-1560178)	13521.62 (11483.59-15856.65)	-0.01 (-0.01--0.01)	23107 (3655-54026)	508.38 (92.64-1154.97)	49217 (8048-112918)	506.14 (88.03-1135.96)	0 (-0.01-0)
Thailand	882296 (757261-1015660)	1403.69 (1212.6-1597.8)	799046 (685712-915153)	1356.7 (1161.66-1559.86)	-0.12 (-0.15--0.08)	11255758 (9665076-13118841)	19178.99 (16663.98-22035.83)	12945213 (11160064-15215918)	17536.75 (15060.31-20577.86)	-0.31 (-0.4--0.23)	417296 (34860-946085)	709.72 (63.33-1627.69)	481835 (53844-1065035)	652.45 (63.95-1488.6)	-0.29 (-0.38--0.21)
Timor-Leste	10465 (8807-12285)	1276.44 (1086.07-1474.68)	19309 (16267-22683)	1284.38 (1093.02-1483.81)	0.02 (0.01-0.02)	108610 (90806-129692)	15674.67 (13320.24-18460.21)	205543 (172280-245710)	15767.47 (13396.52-18577.19)	0.02 (0.01-0.02)	4020 (392-9106)	581.36 (65.76-1313.41)	7678 (758-17586)	588.7 (65.13-1339.22)	0.06 (0.05-0.07)
Togo	46526 (38707-55002)	1175.6 (1000.32-1354.39)	107119 (90513-124820)	1171.61 (997.19-1349.6)	-0.01 (-0.01--0.01)	445168 (370070-538596)	14622.85 (12406.61-17271.31)	1141617 (955308-1378419)	14598.14 (12386.06-17232.59)	0 (0-0)	16340 (2022-37488)	542.65 (82.42-1203.08)	42301 (5772-96101)	544.41 (84.26-1213.7)	0.01 (0.01-0.02)
Tokelau	19 (16-22)	1175.54 (1004.92-1361.06)	17 (14-19)	1166.71 (997.5-1350.52)	-0.04 (-0.04--0.03)	203 (172-240)	14198.03 (12076.4-16605.24)	194 (165-226)	14069.17 (11959.79-16458.19)	-0.04 (-0.05--0.03)	8 (1-17)	527.36 (65.64-1174.8)	7 (1-16)	521.32 (64.93-1164.07)	-0.05 (-0.06--0.04)
Tonga	1226 (1019-1451)	1175.03 (1004.21-1359.34)	1276 (1076-1485)	1175.77 (1005.05-1359.77)	0 (0-0.01)	12263 (10253-14570)	14183.74 (12062.15-16590.92)	13923 (11801-16407)	14199.04 (12074.06-16612.03)	0 (0-0.01)	457 (50-1041)	529.03 (66.18-1171.5)	517 (61-1163)	527.09 (65.62-1174.81)	0 (-0.01-0)
Trinidad and Tobago	15356 (12916-17883)	1172.18 (999.56-1345.59)	14995 (12751-17197)	1169.01 (996.73-1342.18)	-0.01 (-0.01--0.01)	169824 (142290-198986)	14198.54 (11972.57-16485.5)	206096 (174679-238283)	14155.63 (11936.02-16433.72)	-0.01 (-0.01--0.01)	6308 (782-13947)	529.42 (72.07-1156.29)	7681 (1186-16799)	525.41 (74.23-1139.57)	-0.02 (-0.02--0.01)
Tunisia	111734 (94457-130587)	1224.37 (1042.35-1414.65)	141064 (120710-163219)	1226.93 (1045.05-1417.98)	0.01 (0.01-0.01)	1211754 (1009295-1438646)	15197.44 (12871.65-17845.06)	1897431 (1617767-2219023)	15266.51 (12929.02-17921.64)	0.02 (0.02-0.02)	47504 (7713-106566)	597.87 (105.96-1305.53)	74124 (14143-158744)	595.23 (109.26-1294.17)	0.01 (0-0.01)
Turkmenistan	42820 (35839-50137)	1103.45 (938.08-1283.9)	57727 (48921-67327)	1087.95 (923.86-1266.27)	-0.05 (-0.05--0.05)	449943 (377903-536834)	13637.75 (11584.54-15977.16)	702054 (593584-826206)	13422.84 (11400.34-15731.63)	-0.06 (-0.06--0.05)	16621 (2593-39006)	509.19 (91.19-1153.22)	26287 (4640-59523)	503.16 (90.63-1136.04)	-0.04 (-0.04--0.03)
Tuvalu	111 (94-129)	1184.08 (1013.03-1369.24)	148 (126-172)	1160.44 (992.81-1344.31)	-0.07 (-0.07--0.07)	1261 (1068-1485)	14362.41 (12220.14-16802.61)	1727 (1464-2031)	13988.66 (11887.12-16360.53)	-0.09 (-0.09--0.09)	47 (6-105)	533.81 (66.82-1190.5)	64 (8-143)	519.83 (65.01-1154.83)	-0.06 (-0.07--0.05)
Türkiye	751474 (636940-881249)	1198.78 (1030.46-1389.43)	987674 (844505-1137498)	1202.63 (1025.98-1387.67)	0.03 (0.01-0.04)	8346603 (7095524-9842935)	14919.25 (12776.1-17447.29)	13291384 (11475385-15372361)	14934.82 (12811.11-17319.6)	0.01 (-0.02-0.04)	329859 (59278-738375)	592.01 (114.76-1291.39)	527555 (107507-1116477)	591.36 (115.75-1272.54)	0.01 (-0.01-0.03)
Uganda	132281 (110381-158308)	757.4 (644.52-887.74)	350039 (291768-417944)	758.64 (645.36-889.46)	0 (0-0.01)	1251782 (1043940-1483678)	9117.58 (7762.41-10634)	3340425 (2789408-3959229)	9175.87 (7819.32-10696.81)	0.02 (0.02-0.02)	46730 (9201-106548)	346.3 (79.89-754.39)	126089 (24580-293841)	352.24 (81.09-773.96)	0.07 (0.07-0.07)
Ukraine	528507 (467982-594803)	1052.1 (927.05-1187.88)	408130 (360557-458776)	1046.04 (921.4-1181.66)	-0.02 (-0.02--0.02)	7685974 (6692141-8762196)	13687.24 (11914.26-15609.42)	6594152 (5746870-7566954)	13592.26 (11831.93-15502.08)	-0.02 (-0.02--0.02)	313915 (92002-642348)	552.3 (149.37-1151.34)	271531 (83932-543384)	548.06 (152.25-1136)	0 (-0.01-0)
United Arab Emirates	23209 (19522-27331)	1133.27 (961.24-1314.51)	102515 (83815-123865)	1147.21 (975-1327.31)	-0.01 (-0.05-0.02)	266234 (222833-317030)	13646.78 (11533.19-16025.59)	1535297 (1288571-1850849)	13611.91 (11512.97-16000.9)	-0.05 (-0.07--0.02)	10487 (1792-22908)	538.34 (99.9-1176.79)	61171 (11980-128408)	536.15 (98.84-1166.49)	-0.04 (-0.06--0.02)

United Kingdom	711416 (631213-802669)	1377.69 (1217.97-1549.6)	797470 (708497-898063)	1387.52 (1222.47-1566.53)	0.02 (0.01-0.03)	10306430 (8929749-11791610)	17325.48 (15027.09-19836.92)	12064770 (10517926-13963773)	17467.16 (15069.84-20164.22)	0.02 (0.02-0.03)	385750 (62976-840407)	644.96 (95.43-1416.58)	452047 (77046-968292)	648.34 (93.61-1413.04)	0.02 (0.01-0.03)
United Republic of Tanzania	198982 (165568-235095)	749.2 (635.43-875.51)	479362 (396929-568094)	770.74 (646.74-904.36)	0.19 (0.14-0.24)	1908492 (1611516-2242671)	8987.91 (7737.5-10352.2)	4860793 (4038449-5780152)	9368.6 (7948.69-10987.9)	0.24 (0.19-0.3)	71641 (14442-163947)	343.37 (79.98-745.17)	184988 (36397-421096)	361.14 (79.96-786.62)	0.27 (0.22-0.32)
United States of America	3301426 (2916927-3705168)	1346.58 (1182.14-1509.83)	3851991 (3433328-4366539)	1322.69 (1174.24-1502.6)	0 (-0.08-0.08)	45796590 (39783191-52334521)	17098.09 (14778.18-19549.94)	57652573 (50084784-66079074)	16750.24 (14529.77-19303.45)	-0.01 (-0.11-0.09)	1704148 (245770-3727734)	635.39 (89.68-1394.86)	2129026 (350575-4628603)	614.68 (89.74-1356.04)	-0.04 (-0.12-0.05)
United States Virgin Islands	1302 (1107-1500)	1183.85 (1009.72-1356.65)	802 (690-918)	1173.33 (1000.56-1345.36)	-0.03 (-0.04--0.02)	15535 (13082-18026)	14380.06 (12129.06-16693.67)	12145 (10427-14112)	14272.6 (12036.35-16568.05)	-0.02 (-0.03--0.01)	582 (78-1272)	538.36 (74.68-1176.8)	455 (78-977)	531.68 (72.22-1144.87)	-0.03 (-0.04--0.02)
Uruguay	29250 (24802-34114)	959.88 (811.03-1121.15)	30321 (25845-35423)	959.47 (810.79-1120.6)	0 (0-0)	369767 (315371-431625)	11749.89 (9947.38-13782.22)	418262 (357930-487885)	11758.22 (9955.59-13792.56)	0 (0-0.01)	14082 (2869-30690)	446.27 (87.6-979.17)	15915 (3412-34532)	444.07 (87.36-977.05)	0 (-0.01-0)
Uzbekistan	240092 (201336-281190)	1102.27 (936.72-1282.32)	381322 (322860-440932)	1098.88 (933.7-1278.7)	-0.01 (-0.01--0.01)	2521487 (2117641-3008444)	13613.24 (11564.5-15951.42)	4725170 (3998863-5559334)	13577.32 (11533.19-15913.33)	-0.01 (-0.01--0.01)	93049 (14356-217717)	507.77 (89.94-1145.06)	176360 (31421-397878)	507.07 (91.96-1139.16)	0 (0-0.01)
Vanuatu	1868 (1563-2203)	1167.9 (997.97-1352.16)	3884 (3287-4532)	1172.25 (1002.11-1356.65)	0.01 (0.01-0.01)	18424 (15341-21996)	14028.09 (11914.74-16410.01)	41663 (35127-49513)	14129.1 (12008.52-16531.16)	0.02 (0.02-0.02)	681 (73-1555)	520.39 (64.35-1156.63)	1542 (179-3489)	523.44 (65.42-1170.31)	0.02 (0.02-0.03)
Venezuela (Bolivarian Republic of)	235727 (200119-275509)	1131.53 (967.87-1303.84)	299182 (254047-344704)	1162.16 (990.95-1341.15)	0.08 (0.07-0.1)	2488176 (2103883-2928086)	13648.9 (11668.19-15821.47)	3871592 (3281413-4478143)	14161.13 (12033.1-16494.83)	0.12 (0.1-0.14)	92994 (11556-208513)	513.14 (73.92-1121.89)	145521 (21341-317774)	530.58 (74.01-1156)	0.11 (0.1-0.13)
Viet Nam	948414 (800717-1112696)	1294.01 (1102.03-1493.73)	1280881 (1093873-1493409)	1281.16 (1090.74-1479.61)	-0.03 (-0.03--0.03)	9976872 (8376281-11930820)	15973.61 (13572.6-18825.76)	16741403 (14213554-19746451)	15775.92 (13409.35-18571.22)	-0.04 (-0.04--0.04)	373420 (36226-859908)	599.5 (66.34-1366.29)	632132 (72787-1418902)	594.74 (66.79-1354.26)	-0.02 (-0.02--0.02)
Yemen	183663 (150475-218895)	1225.9 (1044.5-1417.98)	465622 (387898-545375)	1224.98 (1042.97-1415.56)	0 (0-0.01)	1676377 (1396310-2007459)	15210.01 (12877.08-17861.93)	4824122 (4022568-5730647)	15221.95 (12890.29-17872.99)	0.01 (0-0.01)	64290 (9684-146205)	588.08 (106.31-1289.31)	185885 (28723-411118)	589.63 (104.87-1282.93)	0.02 (0.01-0.02)
Zambia	67114 (55753-80406)	806.95 (679.64-935.52)	171414 (142064-202716)	804.34 (677.58-932.01)	-0.05 (-0.07--0.03)	653848 (544647-775549)	10069.68 (8497.52-11752.92)	1749234 (1450045-2087026)	10080.75 (8513.1-11789.21)	-0.05 (-0.07--0.03)	24711 (5099-56501)	388.35 (95.56-850.39)	66588 (14070-150831)	390.81 (94.92-845.6)	0.01 (-0.01-0.03)
Zimbabwe	113894 (94508-135292)	1018.16 (862.2-1182.27)	173185 (143955-202620)	1020.53 (864.36-1184.7)	0.01 (0.01-0.01)	1085732 (900661-1297850)	12439.12 (10525.52-14566.77)	1793179 (1493090-2134515)	12527.6 (10603.94-14662.91)	0.02 (0.02-0.03)	40195 (5866-91237)	466.27 (82.4-1014.88)	66492 (10159-151665)	467.74 (82.34-1021.64)	0.02 (0.02-0.03)

Supplementary Table S5 Frontier incidence, prevalence, DALYs, and effective differences by countries and regions, 2021.

Incidence					Prevalence					DALYs				
Location	SDI	ASIR2021	Frontier Incidence	Effective difference	Location	SDI	ASIR2021	Frontier Incidence	Effective difference	Location	SDI	ASIR2021	Frontier Incidence	Effective difference
		pre-100,000	pre-100,000	pre-100,000			pre-100,000	pre-100,000	pre-100,000			pre-100,000	pre-100,000	pre-100,000
Brazil	0.65	1510.77	691.12	819.65	Belgium	0.85	21751.47	8218.23	13533.24	Belgium	0.85	800.36	312.86	487.50
Belgium	0.85	1506.95	691.05	815.90	Italy	0.81	19244.27	8252.91	10991.35	Italy	0.81	717.20	313.37	403.83

Italy	0.81	1494.64	691.10	803.54	Germany	0.90	19203.72	8238.19	10965.53	Germany	0.90	707.40	313.31	394.09
Paraguay	0.64	1440.78	691.11	749.67	Brazil	0.65	18631.33	8298.14	10333.19	Spain	0.77	688.24	313.34	374.90
Norway	0.92	1425.49	690.91	734.58	Spain	0.77	18266.01	8252.68	10013.33	Brazil	0.65	680.61	312.83	367.78
Germany	0.90	1421.37	691.08	730.30	Greece	0.79	18254.82	8248.29	10006.52	Greece	0.79	674.80	313.37	361.43
Sweden	0.89	1409.70	691.13	718.57	Sweden	0.89	17826.53	8238.73	9587.80	France	0.84	661.31	313.29	348.01
Greece	0.79	1401.01	691.02	709.99	San Marino	0.89	17738.70	8236.31	9502.39	San Marino	0.89	660.00	313.29	346.71
Spain	0.77	1393.83	691.10	702.73	Norway	0.92	17700.09	8228.35	9471.74	Sweden	0.89	657.82	313.22	344.59
United Kingdom	0.86	1387.52	691.10	696.42	Portugal	0.74	17692.88	8278.04	9414.85	Cyprus	0.84	657.50	312.95	344.55
Monaco	0.91	1375.74	691.20	684.55	Ireland	0.87	17632.30	8229.40	9402.90	Israel	0.81	656.91	313.42	343.49
Portugal	0.74	1375.15	691.09	684.06	Monaco	0.91	17639.08	8236.21	9402.87	Portugal	0.74	656.01	313.34	342.67
San Marino	0.89	1374.79	691.14	683.65	Cyprus	0.84	17622.86	8236.75	9386.11	Monaco	0.91	655.88	313.22	342.65
Ireland	0.87	1374.16	691.15	683.01	Israel	0.81	17572.11	8253.94	9318.16	Ireland	0.87	654.12	312.95	341.18
Israel	0.81	1370.09	690.95	679.14	Finland	0.86	17477.69	8202.84	9274.86	Norway	0.92	653.12	313.22	339.90
Cyprus	0.84	1370.05	691.10	678.95	France	0.84	17478.90	8222.92	9255.98	Thailand	0.68	652.45	313.23	339.22
Finland	0.86	1366.62	690.92	675.70	Thailand	0.68	17536.75	8297.08	9239.67	Iceland	0.88	651.01	313.22	337.79
Iceland	0.88	1366.05	691.06	674.99	United Kingdom	0.86	17467.16	8227.94	9239.22	Malta	0.80	650.36	313.32	337.03
Andorra	0.87	1364.67	691.07	673.60	Iceland	0.88	17439.61	8236.35	9203.26	Finland	0.86	649.91	313.08	336.83
Malta	0.80	1363.74	691.05	672.69	Malta	0.80	17448.60	8249.51	9199.09	United Kingdom	0.86	648.34	313.34	335.00
France	0.84	1360.59	691.14	669.45	Paraguay	0.64	17467.73	8296.77	9170.97	Andorra	0.87	646.76	312.83	333.93
Thailand	0.68	1356.70	691.11	665.59	Andorra	0.87	17391.72	8246.72	9145.00	Luxembourg	0.88	641.87	313.34	328.53
Netherlands	0.89	1351.72	690.96	660.76	Netherlands	0.89	17015.11	8241.74	8773.38	Iran (Islamic Republic of)	0.70	641.56	313.30	328.25
Luxembour g	0.88	1333.63	691.01	642.62	Luxembourg	0.88	16915.46	8243.51	8671.96	Paraguay	0.64	640.61	313.23	327.38
Indonesia	0.66	1331.48	691.02	640.46	United States of America	0.86	16750.24	8237.97	8512.27	Netherlands	0.89	628.54	312.85	315.69
Philippines	0.65	1330.93	691.15	639.78	Austria	0.85	16466.12	8242.33	8223.79	Austria	0.85	615.73	313.33	302.39
United States of America	0.86	1322.69	691.11	631.58	Iran (Islamic Republic of)	0.70	16492.36	8297.62	8194.74	Egypt	0.61	615.17	313.34	301.83
Austria	0.85	1314.38	691.11	623.27	Canada	0.87	16333.55	8242.52	8091.04	United States of America	0.86	614.68	313.23	301.45
Iran (Islamic Republic of)	0.70	1297.77	691.17	606.60	Philippines	0.65	16273.79	8295.59	7978.20	Indonesia	0.66	612.37	312.84	299.53

Myanmar	0.53	1291.88	691.08	600.81	Indonesia	0.66	16269.49	8296.59	7972.91	Philippines	0.65	609.09	313.35	295.74
Sri Lanka	0.70	1290.90	690.86	600.03	Greenland	0.83	16078.52	8233.11	7845.41	Canada	0.87	607.71	312.84	294.87
Cambodia	0.47	1287.01	691.07	595.94	Myanmar	0.53	15924.77	8297.14	7627.63	Syrian Arab Republic	0.62	606.42	313.34	293.08
Mauritius	0.72	1284.94	690.89	594.05	Sri Lanka	0.70	15890.54	8294.97	7595.57	Algeria	0.66	595.45	312.92	282.53
Switzerland	0.93	1284.81	690.91	593.90	Switzerland	0.93	15818.27	8228.26	7590.01	Sudan	0.54	595.27	312.85	282.42
Lao People's Democratic Republic	0.49	1284.51	690.89	593.62	Cambodia	0.47	15875.26	8295.15	7580.11	Tunisia	0.68	595.23	313.21	282.02
Timor-Leste	0.44	1284.38	691.12	593.26	Mauritius	0.72	15779.33	8295.91	7483.43	Myanmar	0.53	595.18	313.44	281.74
Viet Nam	0.63	1281.16	690.88	590.28	Viet Nam	0.63	15775.92	8297.04	7478.88	Viet Nam	0.63	594.74	313.37	281.37
Egypt	0.61	1273.10	690.91	582.19	Lao People's Democratic Republic	0.49	15770.99	8300.42	7470.57	Greenland	0.83	593.26	313.32	279.94
Nepal	0.43	1272.83	691.12	581.71	Timor-Leste	0.44	15767.47	8299.02	7468.44	Cambodia	0.47	592.98	313.12	279.86
Seychelles	0.73	1270.45	691.08	579.37	Nepal	0.43	15717.90	8295.47	7422.44	Lebanon	0.74	592.73	313.20	279.52
Denmark	0.90	1266.41	691.07	575.34	Egypt	0.61	15670.29	8299.29	7370.99	Sri Lanka	0.70	592.23	313.23	279.00
Syrian Arab Republic	0.62	1260.82	691.21	569.62	Syrian Arab Republic	0.62	15609.45	8295.91	7313.53	Morocco	0.56	592.13	313.40	278.73
Greenland	0.83	1248.06	690.89	557.16	Seychelles	0.73	15527.62	8295.04	7232.58	Lao People's Democratic Republic	0.49	591.40	312.94	278.46
Canada	0.87	1247.29	691.08	556.21	Denmark	0.90	15422.39	8238.39	7184.00	Switzerland	0.93	590.17	312.92	277.26
Maldives	0.65	1238.55	691.13	547.42	Tunisia	0.68	15266.51	8297.15	6969.36	Palestine	0.63	590.22	313.23	276.99
India	0.58	1232.92	691.30	541.63	Lebanon	0.74	15238.44	8269.53	6968.90	Russian Federation	0.81	589.48	312.84	276.64
Tunisia	0.68	1226.93	691.20	535.73	Nigeria	0.50	15259.08	8296.46	6962.62	Yemen	0.45	589.63	313.41	276.22
Sudan	0.54	1226.29	691.11	535.17	Yemen	0.45	15221.95	8295.97	6925.98	Libya	0.73	589.10	313.21	275.88
Nigeria	0.50	1225.57	691.06	534.51	Afghanistan	0.34	15219.55	8296.29	6923.26	Timor-Leste	0.44	588.70	313.34	275.36
Yemen	0.45	1224.98	691.21	533.78	Sudan	0.54	15216.83	8296.79	6920.05	Mauritius	0.72	586.53	313.41	273.13
Morocco	0.56	1223.77	691.10	532.67	Morocco	0.56	15201.02	8294.39	6906.63	Afghanistan	0.34	585.63	313.44	272.19
Kuwait	0.85	1223.67	691.11	532.56	Algeria	0.66	15170.66	8296.81	6873.85	Iraq	0.66	584.75	313.41	271.34
Pakistan	0.50	1223.13	690.89	532.24	Palestine	0.63	15147.20	8297.85	6849.35	Jordan	0.73	582.47	313.34	269.13
Algeria	0.66	1221.98	691.24	530.74	Libya	0.73	15082.94	8296.74	6786.20	Kuwait	0.85	581.90	312.94	268.95
Palestine	0.63	1219.89	691.16	528.73	Kuwait	0.85	14984.55	8239.66	6744.90	Denmark	0.90	581.19	312.87	268.32
Bangladesh	0.49	1219.24	691.03	528.21	Iraq	0.66	15038.58	8295.28	6743.30	Seychelles	0.73	580.57	313.44	267.12

Afghanistan	0.34	1219.25	691.13	528.12	Maldives	0.65	14991.69	8300.11	6691.57	Nigeria	0.50	569.67	313.36	256.31
Lebanon	0.74	1217.65	691.06	526.59	India	0.58	14909.22	8298.64	6610.57	Nepal	0.43	568.37	312.82	255.55
Libya	0.73	1217.46	690.92	526.54	Jordan	0.73	14843.49	8296.12	6547.37	Maldives	0.65	562.08	313.10	248.98
Iraq	0.66	1213.19	690.98	522.21	Guinea	0.34	14621.66	8296.04	6325.62	Oman	0.77	557.10	313.23	243.87
Bhutan	0.47	1208.09	691.13	516.96	Bangladesh	0.49	14622.91	8301.45	6321.47	Saudi Arabia	0.82	555.39	313.42	241.96
Malaysia	0.74	1206.73	691.27	515.46	Guinea-Bissau	0.35	14614.35	8297.42	6316.93	Bahrain	0.75	551.51	313.22	238.30
Jordan	0.73	1201.47	691.13	510.34	Burkina Faso	0.29	14610.82	8297.20	6313.62	Ukraine	0.76	548.06	313.27	234.79
Cook Islands	0.78	1181.86	691.02	490.85	Ghana	0.56	14609.79	8297.68	6312.12	Burkina Faso	0.29	546.10	313.27	232.83
Haiti	0.45	1181.07	691.11	489.96	Togo	0.41	14598.14	8296.87	6301.27	Ghana	0.56	545.15	313.21	231.93
Bahamas	0.81	1181.01	691.15	489.85	Pakistan	0.50	14570.79	8292.63	6278.15	Mauritania	0.50	545.08	313.24	231.84
Kiribati	0.53	1178.73	690.87	487.85	Benin	0.37	14559.28	8296.46	6262.82	Togo	0.41	544.41	312.83	231.58
Guinea	0.34	1177.00	691.02	485.98	Mauritania	0.50	14556.42	8297.88	6258.54	Guinea	0.34	544.31	313.34	230.97
Puerto Rico	0.83	1177.03	691.11	485.93	Gambia	0.41	14530.02	8295.41	6234.60	Guinea-Bissau	0.35	542.99	312.82	230.17
Belize	0.61	1177.15	691.24	485.90	Mali	0.27	14516.45	8297.07	6219.38	Poland	0.81	543.31	313.21	230.10
Guyana	0.65	1176.93	691.10	485.83	Senegal	0.41	14508.51	8297.70	6210.82	Niger	0.17	543.59	314.23	229.36
Antigua and Barbuda	0.75	1176.32	690.87	485.45	Niger	0.17	14539.47	8333.40	6206.07	Benin	0.37	542.17	312.94	229.22
Burkina Faso	0.29	1176.22	690.86	485.36	Chad	0.24	14502.42	8297.52	6204.90	Sao Tome and Principe	0.51	542.03	312.92	229.11
Tonga	0.63	1175.77	691.04	484.73	Cameroon	0.48	14498.08	8297.86	6200.22	India	0.58	540.99	313.21	227.78
Barbados	0.75	1175.27	690.88	484.39	Sierra Leone	0.36	14478.41	8299.14	6179.27	Senegal	0.41	540.26	313.34	226.91
Guinea-Bissau	0.35	1174.38	691.13	483.25	Sao Tome and Principe	0.51	14456.37	8296.98	6159.39	Cameroon	0.48	539.80	312.90	226.90
Bermuda	0.82	1174.28	691.12	483.16	Russian Federation	0.81	14379.09	8245.09	6134.01	Cabo Verde	0.53	539.81	313.22	226.59
Jamaica	0.68	1174.29	691.27	483.02	Bhutan	0.47	14430.27	8297.10	6133.17	Colombia	0.66	539.64	313.35	226.29
United States Virgin Islands	0.82	1173.33	690.85	482.48	Cabo Verde	0.53	14422.31	8297.15	6125.16	Sierra Leone	0.36	539.02	312.85	226.17
Ghana	0.56	1172.96	690.99	481.97	Liberia	0.35	14413.91	8297.55	6116.35	Gambia	0.41	538.73	312.88	225.85
Suriname	0.63	1172.83	691.14	481.69	Bahamas	0.81	14343.18	8248.41	6094.77	Mali	0.27	538.85	313.41	225.44
Mauritania	0.50	1172.63	691.13	481.50	Colombia	0.66	14382.09	8295.60	6086.49	Chad	0.24	538.42	313.43	225.00
Vanuatu	0.47	1172.25	690.90	481.35	Puerto Rico	0.83	14317.03	8231.09	6085.95	Malaysia	0.74	537.85	313.37	224.48

Benin	0.37	1171.89	690.90	481.00	El Salvador	0.56	14345.72	8295.71	6050.00	El Salvador	0.56	537.03	313.21	223.83
Togo	0.41	1171.61	690.92	480.70	United States Virgin Islands	0.82	14272.60	8227.48	6045.12	United Arab Emirates	0.85	536.15	313.24	222.91
Nauru	0.63	1171.75	691.09	480.67	Haiti	0.45	14339.78	8297.01	6042.76	Puerto Rico	0.83	535.12	313.21	221.91
Gambia	0.41	1171.37	690.92	480.45	Barbados	0.75	14292.50	8271.53	6020.98	Bermuda	0.82	534.40	312.89	221.51
Saint Kitts and Nevis	0.75	1171.39	691.02	480.37	Poland	0.81	14258.61	8248.25	6010.37	Bahamas	0.81	533.91	313.22	220.68
Chad	0.24	1171.29	691.05	480.24	Cook Islands	0.78	14255.07	8248.41	6006.66	Barbados	0.75	533.58	313.28	220.30
Mali	0.27	1170.64	691.16	479.48	Bermuda	0.82	14239.41	8239.39	6000.03	Qatar	0.85	532.80	312.87	219.93
Solomon Islands	0.43	1170.11	690.97	479.15	Antigua and Barbuda	0.75	14278.95	8287.10	5991.85	Antigua and Barbuda	0.75	532.29	312.90	219.39
Niger	0.17	1170.77	692.09	478.68	Malaysia	0.74	14260.88	8272.47	5988.41	United States Virgin Islands	0.82	531.68	312.88	218.80
Dominican Republic	0.62	1169.62	691.03	478.58	Guyana	0.65	14282.58	8296.86	5985.73	Jamaica	0.68	531.53	313.23	218.30
Fiji	0.68	1169.52	691.03	478.49	Kiribati	0.53	14255.59	8297.19	5958.40	Mexico	0.66	530.62	313.35	217.27
Sierra Leone	0.36	1169.24	690.93	478.31	Suriname	0.63	14248.98	8298.72	5950.26	Venezuela (Bolivarian Republic of)	0.60	530.58	313.34	217.24
Trinidad and Tobago	0.77	1169.01	691.42	477.59	Belize	0.61	14249.40	8302.68	5946.72	Belize	0.61	530.30	313.21	217.09
Saint Lucia	0.67	1168.76	691.26	477.50	Saint Kitts and Nevis	0.75	14177.84	8245.53	5932.31	Bangladesh	0.49	529.83	312.93	216.89
American Samoa	0.72	1168.44	691.19	477.25	Jamaica	0.68	14226.31	8299.16	5927.15	Cuba	0.67	529.47	312.97	216.50
Micronesia (Federated States of)	0.59	1168.36	691.12	477.24	Trinidad and Tobago	0.77	14155.63	8253.54	5902.09	Honduras	0.51	527.94	312.80	215.15
Marshall Islands	0.57	1168.57	691.40	477.17	Tonga	0.63	14199.04	8297.18	5901.87	Dominican Republic	0.62	527.84	313.25	214.59
Saint Vincent and the Grenadines	0.64	1168.29	691.19	477.10	Saudi Arabia	0.82	14115.56	8240.28	5875.27	Saint Kitts and Nevis	0.75	527.92	313.33	214.58
Cameroon	0.48	1168.14	691.15	476.99	Dominican Republic	0.62	14169.24	8297.52	5871.72	Costa Rica	0.70	527.91	313.40	214.51
Niue	0.73	1168.03	691.15	476.88	Saint Lucia	0.67	14161.11	8297.00	5864.11	Cook Islands	0.78	527.28	313.26	214.02
Guam	0.80	1167.14	691.13	476.01	Venezuela (Bolivarian Republic of)	0.60	14161.13	8298.17	5862.96	Tonga	0.63	527.09	313.22	213.87
Senegal	0.41	1166.97	691.07	475.90	Oman	0.77	14115.28	8252.70	5862.58	Suriname	0.63	526.65	312.90	213.75

Samoa	0.59	1166.92	691.15	475.77	Nauru	0.63	14158.58	8298.04	5860.53	Haiti	0.45	527.01	313.35	213.66
Tokelau	0.69	1166.71	691.14	475.58	Honduras	0.51	14156.32	8297.44	5858.87	Belarus	0.78	526.93	313.44	213.49
Colombia	0.66	1166.75	691.19	475.56	Guatemala	0.54	14154.35	8297.70	5856.65	Republic of Moldova	0.73	526.37	312.95	213.42
Sao Tome and Principe	0.51	1166.22	691.02	475.21	Cuba	0.67	14147.38	8297.47	5849.90	Latvia	0.83	526.11	312.87	213.24
Dominica	0.75	1166.34	691.23	475.11	Vanuatu	0.47	14129.10	8297.56	5831.54	Liberia	0.35	526.60	313.40	213.20
Cuba	0.67	1166.08	691.13	474.95	Mexico	0.66	14127.41	8297.23	5830.18	Bhutan	0.47	526.35	313.24	213.11
Papua New Guinea	0.42	1165.81	691.03	474.78	Dominica	0.75	14091.22	8264.31	5826.90	Saint Lucia	0.67	525.64	312.84	212.80
El Salvador	0.56	1165.35	691.09	474.26	Guam	0.80	14056.48	8246.50	5809.97	Kiribati	0.53	525.75	313.40	212.35
Liberia	0.35	1164.51	690.89	473.63	Saint Vincent and the Grenadines	0.64	14107.49	8300.90	5806.59	Trinidad and Tobago	0.77	525.41	313.42	211.98
Saudi Arabia	0.82	1162.99	691.06	471.94	Costa Rica	0.70	14094.56	8295.75	5798.81	Nicaragua	0.52	524.90	313.22	211.69
Grenada	0.67	1162.45	691.04	471.41	Solomon Islands	0.43	14096.00	8298.68	5797.32	Guatemala	0.54	524.95	313.26	211.68
Cabo Verde	0.53	1162.30	691.09	471.21	Fiji	0.68	14089.00	8296.01	5792.99	Guyana	0.65	524.86	313.20	211.66
Venezuela (Bolivarian Republic of)	0.60	1162.16	691.10	471.06	Niue	0.73	14087.91	8297.07	5790.84	Dominica	0.75	524.43	313.24	211.19
Northern Mariana Islands	0.77	1160.96	691.02	469.93	Micronesia (Federated States of)	0.59	14078.19	8297.75	5780.44	Saint Vincent and the Grenadines	0.64	524.41	313.34	211.08
Tuvalu	0.58	1160.44	690.89	469.55	Tokelau	0.69	14069.17	8295.29	5773.88	Grenada	0.67	523.74	313.22	210.52
Guatemala	0.54	1159.38	691.07	468.31	Grenada	0.67	14061.13	8296.55	5764.58	Guam	0.80	523.53	313.22	210.31
Honduras	0.51	1159.23	691.08	468.15	American Samoa	0.72	14058.93	8296.35	5762.58	Vanuatu	0.47	523.44	313.27	210.17
Oman	0.77	1157.15	690.85	466.31	Marshall Islands	0.57	14055.76	8297.36	5758.40	Estonia	0.84	523.11	313.30	209.81
Mexico	0.66	1155.35	691.25	464.10	Bahrain	0.75	14027.17	8272.36	5754.81	Nauru	0.63	522.68	313.37	209.31
Costa Rica	0.70	1154.78	691.02	463.76	Samoa	0.59	14039.23	8297.78	5741.45	Solomon Islands	0.43	522.13	313.27	208.86
Bahrain	0.75	1150.76	691.06	459.70	Nicaragua	0.52	14032.73	8296.96	5735.77	Tokelau	0.69	521.32	313.27	208.05
Nicaragua	0.52	1148.34	691.27	457.08	Papua New Guinea	0.42	14021.85	8300.67	5721.19	Micronesia (Federated States of)	0.59	520.87	312.94	207.93
United Arab Emirates	0.85	1147.21	691.17	456.04	Northern Mariana Islands	0.77	13954.79	8239.71	5715.08	Niue	0.73	520.70	312.85	207.86
Palau	0.75	1146.66	690.93	455.74	Tuvalu	0.58	13988.66	8295.85	5692.82	Panama	0.71	520.94	313.21	207.73

Panama	0.71	1141.54	691.10	450.44	New Zealand	0.85	13912.65	8244.51	5668.14	Fiji	0.68	520.45	313.25	207.20
Poland	0.81	1131.81	691.21	440.60	Panama	0.71	13876.05	8297.38	5578.67	Tuvalu	0.58	519.83	312.88	206.94
New Zealand	0.85	1125.09	691.02	434.07	Palau	0.75	13739.06	8256.17	5482.89	Samoa	0.59	518.98	312.83	206.15
Qatar	0.85	1120.11	690.96	429.15	Hungary	0.79	13659.57	8249.88	5409.70	Northern Mariana Islands	0.77	519.07	313.24	205.82
Kazakhstan	0.73	1102.85	690.90	411.96	United Arab Emirates	0.85	13611.91	8219.21	5392.70	American Samoa	0.72	518.16	312.89	205.27
Kyrgyzstan	0.60	1101.59	691.15	410.43	Montenegro	0.80	13632.03	8242.41	5389.61	Papua New Guinea	0.42	518.10	313.22	204.88
Mongolia	0.62	1101.20	691.08	410.12	Slovakia	0.81	13618.05	8239.13	5378.92	Hungary	0.79	517.97	313.47	204.51
Uzbekistan	0.66	1098.88	691.03	407.86	Kazakhstan	0.73	13656.47	8297.18	5359.29	Marshall Islands	0.57	517.35	313.22	204.14
Armenia	0.70	1097.98	691.10	406.88	Romania	0.77	13609.33	8251.28	5358.05	Romania	0.77	517.26	313.24	204.02
Tajikistan	0.54	1097.00	691.16	405.84	Bulgaria	0.77	13595.28	8241.19	5354.08	Pakistan	0.50	517.34	313.40	203.94
Georgia	0.85	1093.89	691.04	402.85	Ukraine	0.76	13592.26	8244.62	5347.64	New Zealand	0.85	517.01	313.30	203.71
Georgia	0.73	1093.89	691.11	402.77	Czechia	0.83	13581.76	8240.27	5341.49	Slovakia	0.81	516.94	313.40	203.54
Azerbaijan	0.69	1092.43	691.15	401.28	Bosnia and Herzegovina	0.72	13632.57	8292.81	5339.76	Albania	0.71	516.38	312.85	203.53
Hungary	0.79	1090.02	691.21	398.80	Croatia	0.80	13584.21	8252.06	5332.15	Croatia	0.80	516.50	313.42	203.07
Bosnia and Herzegovina	0.72	1089.27	691.21	398.06	Armenia	0.70	13628.90	8297.51	5331.39	Montenegro	0.80	516.27	313.43	202.84
Australia	0.84	1088.74	690.89	397.86	Mongolia	0.62	13624.59	8296.40	5328.18	Bosnia and Herzegovina	0.72	515.27	313.21	202.06
Slovakia	0.81	1088.42	691.01	397.41	Georgia	0.85	13553.71	8243.22	5310.49	Serbia	0.79	514.68	313.33	201.35
Russian Federation	0.81	1088.30	691.03	397.27	Kyrgyzstan	0.60	13622.21	8315.74	5306.46	Bulgaria	0.77	514.42	313.23	201.19
Turkmenistan	0.68	1087.95	690.85	397.10	Albania	0.71	13598.05	8296.43	5301.62	Czechia	0.83	514.44	313.33	201.11
Romania	0.77	1087.77	690.92	396.86	Serbia	0.79	13545.29	8244.17	5301.13	Slovenia	0.84	513.87	313.46	200.41
Montenegro	0.80	1087.93	691.13	396.79	Slovenia	0.84	13531.47	8238.77	5292.70	North Macedonia	0.75	512.39	313.23	199.17
Croatia	0.80	1087.90	691.13	396.77	Uzbekistan	0.66	13577.32	8299.74	5277.58	Armenia	0.70	510.08	313.40	196.67
Czechia	0.83	1087.51	690.98	396.52	North Macedonia	0.75	13534.57	8257.73	5276.84	Kyrgyzstan	0.60	509.39	313.37	196.02
Bulgaria	0.77	1086.68	691.13	395.55	Qatar	0.85	13503.71	8227.31	5276.40	Kazakhstan	0.73	508.64	312.86	195.77
Slovenia	0.84	1084.46	691.14	393.32	Georgia	0.73	13553.71	8293.48	5260.22	Palau	0.75	508.56	312.83	195.73
Albania	0.71	1084.70	691.40	393.30	Azerbaijan	0.69	13532.87	8297.59	5235.29	Mongolia	0.62	507.53	313.08	194.45
North Macedonia	0.75	1084.44	691.31	393.13	Tajikistan	0.54	13521.62	8295.40	5226.22	Uzbekistan	0.66	507.07	313.35	193.72

Serbia	0.79	1080.23	691.07	389.16	Australia	0.84	13338.06	8203.54	5134.53	Azerbaijan	0.69	506.66	313.34	193.32
Taiwan (Province of China)	0.87	1055.29	691.07	364.22	Turkmenista n	0.68	13422.84	8297.06	5125.77	Tajikistan	0.54	506.14	313.21	192.93
South Africa	0.68	1051.35	691.08	360.27	Taiwan (Province of China)	0.87	13042.93	8235.84	4807.09	Georgia	0.85	505.78	312.89	192.89
Ukraine	0.76	1046.04	691.24	354.80	Belarus	0.78	13015.65	8250.79	4764.86	Georgia	0.73	505.78	312.91	192.87
Gabon	0.63	1021.22	691.10	330.12	Latvia	0.83	12978.48	8239.89	4738.59	Turkmenista n	0.68	503.16	312.95	190.21
Zimbabwe	0.47	1020.53	691.04	329.49	South Africa	0.68	12990.88	8297.55	4693.33	Australia	0.84	494.98	313.25	181.72
Angola	0.45	1020.29	690.91	329.38	Estonia	0.84	12900.48	8219.98	4680.50	Lithuania	0.86	487.22	313.36	173.86
Central African Republic	0.31	1017.46	691.04	326.42	Republic of Moldova	0.73	12971.64	8294.61	4677.04	Taiwan (Province of China)	0.87	485.07	313.33	171.73
Namibia	0.62	1017.47	691.24	326.23	Angola	0.45	12500.79	8297.68	4203.11	South Africa	0.68	480.97	312.91	168.07
Lesotho	0.51	1016.08	691.12	324.96	Lesotho	0.51	12483.69	8298.05	4185.64	Angola	0.45	465.75	312.83	152.92
Eswatini	0.59	1015.20	691.08	324.12	Eswatini	0.59	12480.77	8297.08	4183.69	Namibia	0.62	465.84	313.22	152.62
Congo	0.58	1013.98	691.09	322.88	Namibia	0.62	12478.70	8297.41	4181.29	Ecuador	0.66	464.61	312.96	151.65
Botswana	0.64	1013.25	691.03	322.22	Central African Republic	0.31	12462.33	8294.87	4167.46	Gabon	0.63	463.95	313.40	150.55
Democratic Republic of the Congo	0.38	1010.12	691.12	319.01	Gabon	0.63	12471.35	8304.78	4166.57	Congo	0.58	462.17	312.88	149.29
Belarus	0.78	1009.66	691.08	318.58	Botswana	0.64	12415.02	8298.88	4116.14	Central African Republic	0.31	461.89	312.89	149.00
Republic of Moldova	0.73	1008.23	691.04	317.19	Ecuador	0.66	12383.18	8297.91	4085.27	Botswana	0.64	461.18	312.90	148.28
Latvia	0.83	1008.08	691.02	317.06	Congo	0.58	12387.53	8322.25	4065.28	Eswatini	0.59	460.13	313.29	146.84
Estonia	0.84	1004.87	691.13	313.74	Democratic Republic of the Congo	0.38	12358.98	8297.58	4061.41	Democratic Republic of the Congo	0.38	459.87	313.40	146.46
Ecuador	0.66	997.34	690.88	306.46	Lithuania	0.86	12244.96	8242.16	4002.81	Lesotho	0.51	458.70	313.25	145.46
Equatorial Guinea	0.66	996.13	690.87	305.26	Equatorial Guinea	0.66	12281.69	8297.05	3984.63	Equatorial Guinea	0.66	457.66	313.23	144.43
Democratic People's Republic of Korea	0.57	990.52	691.13	299.39	Chile	0.77	11862.87	8248.49	3614.38	Chile	0.77	445.42	312.97	132.45
Lithuania	0.86	976.09	690.90	285.19	Republic of Korea	0.89	11812.12	8235.59	3576.53	Uruguay	0.72	444.07	312.84	131.23
China	0.72	975.61	690.95	284.65	Democratic People's	0.57	11798.27	8304.24	3494.03	Republic of Korea	0.89	444.10	312.91	131.19

					Republic of Korea									
Chile	0.77	960.08	691.31	268.77	China	0.72	11777.51	8294.66	3482.85	China	0.72	443.65	313.40	130.25
Uruguay	0.72	959.47	691.23	268.24	Uruguay	0.72	11758.22	8292.66	3465.56	Democratic People's Republic of Korea	0.57	441.94	313.24	128.69
Argentina	0.72	955.45	691.04	264.41	Argentina	0.72	11636.39	8296.98	3339.41	Argentina	0.72	441.01	313.24	127.78
Republic of Korea	0.89	937.81	691.04	246.77	Bolivia (Plurinational State of)	0.60	10894.77	8297.72	2597.05	Japan	0.87	415.34	313.23	102.11
Bolivia (Plurinational State of)	0.60	912.62	691.03	221.60	Japan	0.87	10818.40	8241.90	2576.50	Bolivia (Plurinational State of)	0.60	409.78	312.91	96.88
Japan	0.87	873.60	691.29	182.31	Brunei Darussalam	0.81	10292.50	8242.57	2049.93	Brunei Darussalam	0.81	393.16	312.83	80.33
Brunei Darussalam	0.81	849.90	690.86	159.04	Zambia	0.51	10080.75	8297.09	1783.67	Zambia	0.51	390.81	312.82	77.99
Peru	0.66	829.96	691.27	138.69	Kenya	0.52	9671.53	8295.31	1376.23	Kenya	0.52	373.46	313.24	60.22
Zambia	0.51	804.34	691.12	113.22	Peru	0.66	9645.45	8298.50	1346.96	Singapore	0.86	370.02	312.84	57.18
Singapore	0.86	803.97	690.98	113.00	Singapore	0.86	9471.09	8203.98	1267.11	Peru	0.66	368.08	312.85	55.23
Kenya	0.52	781.63	691.06	90.56	United Republic of Tanzania	0.45	9368.60	8297.18	1071.42	United Republic of Tanzania	0.45	361.14	313.41	47.73
United Republic of Tanzania	0.45	770.74	691.25	79.49	Mozambique	0.33	9206.13	8293.03	913.11	Rwanda	0.44	353.02	313.37	39.66
Mozambique	0.33	762.48	691.03	71.45	Rwanda	0.44	9178.77	8296.54	882.23	Uganda	0.42	352.24	312.85	39.40
Malawi	0.38	759.04	691.06	67.98	Uganda	0.42	9175.87	8299.00	876.87	Malawi	0.38	351.95	313.25	38.70
Rwanda	0.44	758.73	691.03	67.69	Malawi	0.38	9155.39	8295.68	859.71	Comoros	0.48	351.44	312.90	38.54
Uganda	0.42	758.64	691.05	67.60	South Sudan	0.28	9129.91	8294.63	835.28	Madagascar	0.40	351.38	313.24	38.14
South Sudan	0.28	757.61	691.01	66.60	Madagascar	0.40	9122.41	8297.59	824.82	Eritrea	0.40	349.48	313.25	36.23
Madagascar	0.40	757.12	691.10	66.03	Eritrea	0.40	9085.39	8295.93	789.46	South Sudan	0.28	347.88	312.86	35.02
Comoros	0.48	753.20	690.99	62.21	Comoros	0.48	9086.74	8299.50	787.24	Mozambique	0.33	347.92	313.26	34.66
Burundi	0.29	752.93	691.08	61.85	Burundi	0.29	9028.71	8298.82	729.88	Burundi	0.29	347.39	312.89	34.49
Eritrea	0.40	750.43	691.13	59.30	Djibouti	0.49	8909.93	8296.54	613.40	Djibouti	0.49	345.08	312.94	32.14
Djibouti	0.49	740.20	690.97	49.23	Ethiopia	0.36	8365.36	8297.52	67.85	Somalia	0.08	349.03	347.88	1.15
Ethiopia	0.36	701.09	691.12	9.97	Somalia	0.08	9098.36	9062.87	35.49	Ethiopia	0.36	313.55	312.84	0.71
Somalia	0.08	751.12	750.60	0.52										

Supplementary Table S6 Predictive analysis of incidence, prevalence, DALYs of migraine in males and females from 2022 to 2050.

Year	Sex	Incidence		Prevalence		DALYs	
		Cases	ASIR pre-100,000	Cases	ASPR pre-100,000	Cases	ASDR pre-100,000
2022	Male	35164012.77	877.91	438367073.10	10671.21	16706575.04	404.49
2023	Male	35624215.13	879.62	443773745.90	10731.79	16946122.68	405.11
2024	Male	36059842.66	881.31	449281963.00	10775.66	17173439.76	405.73
2025	Male	36427369.39	882.75	454770619.50	10795.57	17368751.42	406.34
2026	Male	36750885.73	883.88	460170061.50	10811.23	17548807.99	406.96
2027	Male	37089511.83	884.77	465470238.80	10843.61	17741711.85	407.58
2028	Male	37476802.63	885.55	470702957.40	10892.20	17955548.44	408.19
2029	Male	37893416.06	886.34	475914455.80	10939.16	18174633.03	408.81
2030	Male	38293661.84	887.21	481142371.80	10971.40	18381135.38	409.43
2031	Male	38652728.22	888.18	486404373.10	10993.82	18574087.46	410.04
2032	Male	38987019.30	889.23	491698599.20	11021.08	18766696.08	410.66
2033	Male	39332597.83	890.31	497011459.50	11060.15	18969562.20	411.28
2034	Male	39708417.59	891.40	502327119.20	11104.17	19180305.06	411.90
2035	Male	40101249.18	892.45	507634408.90	11142.09	19388913.52	412.51
2036	Male	40483157.20	893.47	512929431.70	11171.28	19589802.63	413.13
2037	Male	40840116.57	894.47	518214426.30	11198.92	19786613.87	413.75
2038	Male	41183157.19	895.46	523494679.70	11232.66	19986401.66	414.36
2039	Male	41534232.39	896.44	528775386.40	11272.31	20191576.49	414.98
2040	Male	41903968.19	897.44	534059682.60	11311.36	20398440.11	415.60
2041	Male	42283459.85	898.44	539348185.50	11345.04	20602367.10	416.21
2042	Male	42655681.26	899.46	544639668.80	11375.15	20802797.03	416.83
2043	Male	43012779.01	900.48	549932204.00	11407.02	21002912.86	417.45
2044	Male	43362089.55	901.49	555224156.90	11443.20	21205516.98	418.06
2045	Male	43716947.59	902.51	560514710.10	11481.18	21410215.39	418.68
2046	Male	44083256.40	903.52	565803881.60	11516.93	21614534.25	419.30
2047	Male	44455097.26	904.53	571092214.60	11549.51	21816941.05	419.91

2048	Male	44822124.00	905.54	576380378.20	11581.52	22018221.84	420.53
2049	Male	45179986.62	906.54	581668863.90	11615.75	22220153.62	421.15
2050	Male	45533519.46	907.55	586957861.10	11652.14	22423445.69	421.77
2022	Female	55934456.50	1440.68	733661986.00	17930.69	27138017.74	662.34
2023	Female	56477739.70	1443.32	742815415.30	17967.01	27414902.89	662.36
2024	Female	57021022.90	1445.56	751923003.70	17988.18	27712851.38	662.65
2025	Female	57564306.10	1446.86	760550650.20	17988.71	28026530.47	663.05
2026	Female	58107589.30	1447.21	768909197.70	17979.90	28349468.03	663.41
2027	Female	58650872.50	1446.93	777433010.60	17973.97	28675794.36	663.67
2028	Female	59194155.70	1446.39	786253370.40	17973.49	29001197.26	663.79
2029	Female	59737438.90	1445.88	795127117.30	17973.81	29323197.80	663.79
2030	Female	60280722.10	1445.56	803773376.60	17971.25	29640950.71	663.71
2031	Female	60824005.30	1445.44	812169417.30	17966.85	29954791.12	663.60
2032	Female	61367288.50	1445.48	820513997.30	17963.93	30265716.11	663.50
2033	Female	61910571.70	1445.59	828978783.80	17964.00	30574930.58	663.42
2034	Female	62453854.90	1445.70	837552163.60	17965.81	30883524.64	663.38
2035	Female	62997138.10	1445.78	846108025.90	17967.32	31192297.33	663.38
2036	Female	63540421.30	1445.81	854570671.50	17967.70	31501706.47	663.39
2037	Female	64083704.50	1445.81	862983630.20	17967.53	31811906.55	663.42
2038	Female	64626987.70	1445.79	871437647.90	17967.58	32122833.36	663.45
2039	Female	65170270.90	1445.76	879967538.70	17968.01	32434300.48	663.47
2040	Female	65713554.10	1445.74	888530863.50	17968.38	32746083.60	663.48
2041	Female	66256837.30	1445.73	897069365.20	17968.39	33057980.29	663.48
2042	Female	66800120.50	1445.73	905570044.40	17968.10	33369842.48	663.48
2043	Female	67343403.69	1445.74	914064554.50	17967.82	33681585.61	663.47
2044	Female	67886686.89	1445.74	922583789.80	17967.72	33993181.48	663.47
2045	Female	68429970.09	1445.75	931125404.70	17967.77	34304642.37	663.46
2046	Female	68973253.29	1445.75	939663852.70	17967.81	34616003.12	663.46
2047	Female	69516536.49	1445.75	948181755.20	17967.79	34927305.49	663.46
2048	Female	70059819.69	1445.75	956685252.60	17967.74	35238587.08	663.46
2049	Female	70603102.89	1445.75	965191848.00	17967.73	35549875.33	663.46

2050	Female	71146386.09	1445.75	973710067.80	17967.77	35861185.84	663.46

Supplementary Table S7 Predictive analysis of incidence, prevalence, and DALYs of migraine across age groups '<20,' '20-54,' and '>55' from 2022 to 2050

Age group	Year	Incidence		Prevalence		DALYs	
		Cases	ASIR pre-100,000	Cases	ASPR pre-100,000	Cases	ASDR pre-100,000
<20 years	2022	37183975.93	1405.07	207976360.7	7876.63	7515775.31	285.14
<20 years	2023	37558418.76	1414.44	210462870.7	7964.25	7515775.31	285.14
<20 years	2024	37919788.11	1423.47	213103110.3	8063.45	7515775.31	285.14
<20 years	2025	38269511.02	1431.88	215842073	8170.99	7515775.31	285.14
<20 years	2026	38608858.76	1439.55	218644434.3	8284.52	7515775.31	285.14
<20 years	2027	38938963.81	1446.48	221487509.4	8402.38	7515775.31	285.14
<20 years	2028	39260835.08	1452.67	224356730.4	8523.35	7515775.31	285.14
<20 years	2029	39575371.31	1458.18	227242741.9	8646.57	7515775.31	285.14
<20 years	2030	39883373.17	1463.08	230139536	8771.40	7515775.31	285.14
<20 years	2031	40185553.9	1467.41	233043254.7	8897.39	7515775.31	285.14
<20 years	2032	40482548.91	1471.25	235951420.1	9024.22	7515775.31	285.14
<20 years	2033	40774924.25	1474.64	238862441.2	9151.65	7515775.31	285.14
<20 years	2034	41063184.18	1477.63	241775296.2	9279.52	7515775.31	285.14
<20 years	2035	41347777.91	1480.27	244689328.9	9407.70	7515775.31	285.14
<20 years	2036	41629105.63	1482.61	247604117.9	9536.10	7515775.31	285.14
<20 years	2037	41907523.84	1484.67	250519392.5	9664.67	7515775.31	285.14
<20 years	2038	42183350.13	1486.49	253434979.1	9793.35	7515775.31	285.14
<20 years	2039	42456867.41	1488.09	256350766	9922.12	7515775.31	285.14
<20 years	2040	42728327.73	1489.51	259266681.5	10050.95	7515775.31	285.14
<20 years	2041	42997955.61	1490.76	262182679.6	10179.82	7515775.31	285.14
<20 years	2042	43265951.08	1491.86	265098730.7	10308.73	7515775.31	285.14
<20 years	2043	43532492.31	1492.83	268014816	10437.65	7515775.31	285.14
<20 years	2044	43797738.05	1493.69	270930923	10566.60	7515775.31	285.14
<20 years	2045	44061829.7	1494.45	273847044.2	10695.55	7515775.31	285.14

<20 years	2046	44324893.24	1495.12	276763174.4	10824.51	7515775.31	285.14
<20 years	2047	44587040.9	1495.71	279679310.3	10953.48	7515775.31	285.14
<20 years	2048	44848372.64	1496.23	282595450	11082.46	7515775.31	285.14
<20 years	2049	45108977.52	1496.69	285511592.1	11211.43	7515775.31	285.14
<20 years	2050	45368934.89	1497.09	288427735.7	11340.41	7515775.31	285.14
20-54 years	2022	47918859.34	1263.41	774356240.2	20407.86	29126773	763.55
20-54 years	2023	48236451.77	1263.92	780554577.7	20426.46	29471406	763.55
20-54 years	2024	48554826.87	1264.26	786752915.3	20431.40	29816040	763.55
20-54 years	2025	48873686.18	1264.39	792951252.9	20431.40	30160673	763.55
20-54 years	2026	49192845.03	1264.34	799149590.4	20431.40	30505307	763.55
20-54 years	2027	49512189.2	1264.20	805347928	20431.40	30849941	763.55
20-54 years	2028	49831648	1264.02	811546265.5	20431.40	31194574	763.55
20-54 years	2029	50151177.73	1263.88	817744603.1	20431.40	31539208	763.55
20-54 years	2030	50470751.33	1263.80	823942940.6	20431.40	31883841	763.55
20-54 years	2031	50790352.07	1263.77	830141278.2	20431.40	32228475	763.55
20-54 years	2032	51109969.61	1263.79	836339615.8	20431.40	32573108	763.55
20-54 years	2033	51429597.54	1263.84	842537953.3	20431.40	32917742	763.55
20-54 years	2034	51749231.89	1263.89	848736290.9	20431.40	33262375	763.55
20-54 years	2035	52068870.21	1263.93	854934628.4	20431.40	33607009	763.55
20-54 years	2036	52388511	1263.95	861132966	20431.40	33951643	763.55
20-54 years	2037	52708153.31	1263.95	867331303.5	20431.40	34296276	763.55
20-54 years	2038	53027796.56	1263.95	873529641.1	20431.40	34640910	763.55
20-54 years	2039	53347440.39	1263.93	879727978.6	20431.40	34985543	763.55
20-54 years	2040	53667084.58	1263.92	885926316.2	20431.40	35330177	763.55
20-54 years	2041	53986729	1263.91	892124653.8	20431.40	35674810	763.55
20-54 years	2042	54306373.55	1263.90	898322991.3	20431.40	36019444	763.55
20-54 years	2043	54626018.19	1263.90	904521328.9	20431.40	36364078	763.55
20-54 years	2044	54945662.88	1263.90	910719666.4	20431.40	36708711	763.55
20-54 years	2045	55265307.61	1263.91	916918004	20431.40	37053345	763.55
20-54 years	2046	55584952.35	1263.91	923116341.5	20431.40	37397978	763.55
20-54 years	2047	55904597.11	1263.92	929314679.1	20431.40	37742612	763.55

20-54 years	2048	56224241.87	1263.92	935513016.7	20431.40	38087245	763.55
20-54 years	2049	56543886.64	1263.92	941711354.2	20431.40	38431879	763.55
20-54 years	2050	56863531.41	1263.92	947909691.8	20431.40	38776512	763.55
>55 years	2022	5936644.67	390.20	189214347.5	12434.26	7186622.66	477.11
>55 years	2023	6081925.74	391.04	193808297.8	12452.66	7311615.22	477.51
>55 years	2024	6227206.81	391.66	198384618.6	12462.46	7431102.25	477.79
>55 years	2025	6372487.89	392.10	202941117.5	12464.92	7552156.11	477.97
>55 years	2026	6517768.96	392.42	207531885.8	12461.43	7672764.07	478.10
>55 years	2027	6663050.03	392.65	212131546.4	12453.44	7793498.93	478.18
>55 years	2028	6808331.11	392.82	216753567.7	12442.31	7914197.67	478.24
>55 years	2029	6953612.18	392.94	221376176.3	12429.33	8034906.70	478.28
>55 years	2030	7098893.25	393.03	226005076	12415.58	8155612.79	478.30
>55 years	2031	7244174.32	393.10	230629561.2	12402.00	8276319.72	478.32
>55 years	2032	7389455.40	393.14	235254874.2	12389.29	8397026.41	478.33
>55 years	2033	7534736.47	393.18	239876835.8	12377.97	8517733.17	478.34
>55 years	2034	7680017.54	393.20	244499339.9	12368.38	8638439.91	478.35
>55 years	2035	7825298.61	393.22	249120459.8	12360.68	8759146.66	478.35
>55 years	2036	7970579.69	393.23	253742342.3	12354.90	8879853.40	478.35
>55 years	2037	8115860.76	393.24	258363771.4	12350.96	9000560.15	478.35
>55 years	2038	8261141.83	393.25	262985766	12348.70	9121266.89	478.36
>55 years	2039	8406422.90	393.25	267607540.6	12347.87	9241973.64	478.36
>55 years	2040	8551703.98	393.25	272229600.7	12348.24	9362680.38	478.36
>55 years	2041	8696985.05	393.26	276851498.8	12349.53	9483387.13	478.36
>55 years	2042	8842266.12	393.26	281473521.5	12351.48	9604093.87	478.36
>55 years	2043	8987547.19	393.26	286095436.1	12353.84	9724800.62	478.36
>55 years	2044	9132828.27	393.26	290717412.3	12356.40	9845507.36	478.36
>55 years	2045	9278109.34	393.26	295339329.2	12358.97	9966214.11	478.36
>55 years	2046	9423390.41	393.26	299961283.1	12361.41	10086920.85	478.36
>55 years	2047	9568671.49	393.26	304583207.3	12363.62	10207627.59	478.36
>55 years	2048	9713952.56	393.26	309205154.2	12365.51	10328334.34	478.36
>55 years	2049	9859233.63	393.26	313827085.8	12367.06	10449041.08	478.36

>55 years	2050	10004514.70	393.26	318449030.2	12368.24	10569747.83	478.36
